# Modified nano magnetic Fe_2_O_3_-MgO as a high active multifunctional heterogeneous catalyst for environmentally beneficial carbon–carbon synthesis

**DOI:** 10.1186/s13065-024-01176-5

**Published:** 2024-04-20

**Authors:** Ehsan Kamali, Fahim Dreekvandy, Abolfazl Mohammadkhani, Akbar Heydari

**Affiliations:** Chemistry Department, Tarbiat Modares University, PO Box: 14155-4838, Tehran, Iran

**Keywords:** Choline formate, Choline cyanide, Multifunctional catalysts, Nanomagnetic heterogeneous catalyst, Knoevenagel and Benzoin condensations

## Abstract

In this study, novel nanomagnetic catalysts, namely Fe_2_O_3_-MgO@choline formate (Ch. F.) and Fe_2_O_3_-MgO@choline cyanide (Ch. CN), were synthesized through immobilizing choline-based ion liquids to magnetic support via a simple and cost-effective methodology. FT-IR, TGA, FE-SEM, VSM, EDS, BET, and XRD techniques were employed to assess and characterize these organic–inorganic compounds. Following the successful preparation of nanoparticles, the catalysts were utilized in Knoevenagel and benzoin condensations. Fe_2_O_3_-MgO@Ch.F. exhibited exceptional activity in Knoevenagel condensation under solvent-free conditions at room temperature, achieving high yields (91–98%) in a short timeframe. Similarly, Fe_2_O_3_-MgO@Ch.CN demonstrated remarkable activity in benzoin condensation under environmentally friendly solvent conditions, yielding higher isolated yields (76–88%). Furthermore, these magnetically recyclable multifunctional catalysts displayed the ability to be reused up to five times without a significant loss in efficiency. Additionally, the heterogeneity of this nanocatalyst was investigated using the hot filtration technique. The findings indicated that the reaction primarily occurs via a heterogeneous pathway.

**Supplementary Information:**

The online version contains supplementary material available at 10.1186/s13065-024-01176-5.

## Introduction

In recent years, significant emphasis has been placed on developing techniques to heterogenize homogeneous catalysts while maintaining their active sites. This approach aims to amalgamate the advantages of selective, homogeneous, reactive catalysis with the recyclability and facile removal of catalysts from the reaction solution [1–9]. The coupling of homogeneous catalysts with inorganic solids is a widely utilized method for achieving heterogeneous synthesis [1, 8, 10]. Numerous homogeneous catalysts for Knoevenagel and benzoin reactions have been explored, with these condensations being recognized for their ability to form carbon–carbon bonds [11–17]. However, homogeneous systems are associated with several drawbacks, including the high cost of catalysts, the challenge of catalyst recovery, catalyst decomposition under basic pH conditions, elevated reaction temperatures, product isolation difficulties, and the use of carcinogenic and environmentally harmful solvents, leading to substantial waste generation [18–20]. To address these challenges and preserve the catalytic active sites inherent in homogeneous counterparts, there is a growing focus on heterogenizing homogeneous catalysts [21]. This research direction has garnered significant attention in both industrial and academic sectors. Choline chloride-based ionic liquids, such as choline azide, choline hydroxide, choline cyanide, and Choline amide serve multifaceted roles in organic processes by acting as safe, cost-effective, and efficient reactants, solvents, and homogeneous catalysts [22–28]. On the other hand, magnetic supports possess various advantageous properties, including low toxicity, cost-effectiveness, extensive surface area, Lewis acid activity, facile production, surface functionalization, rapid dispersion in processes, high conductivity, and efficient recoverability through external magnets [22, 29–36]. In the context of multifunctional catalysts for heterogeneous modification applications, it is imperative to investigate the activity of coordinated cholines on the magnetic surface. Considering the magnetic attraction and potential for agglomeration, incorporating coating stabilizers becomes necessary [37, 38]. The MgO shell acts as a shield for magnetic nanoparticles, preventing further oxidation and aggregation of the magnetic core. Notably, MgO stands out as a superior support compared to alternatives due to possessing Lewis acidic and Lewis basic sites, enabling stabilization of reaction intermediates during catalysis [39–42]. The integration of choline-based ionic liquid as a homogeneous catalyst with iron-magnesium oxide as a super-magnetic support facilitates the synthesis of highly active, multifunctional, and recyclable catalysts. These versatile catalysts are not only cost-effective and environmentally friendly but also hold significant appeal across a wide spectrum of applications. Fe_2_O_3_@MgO effectively stabilizes choline-based ionic liquids as magnetic support in diverse applications. This study began with the novel and remarkably simple synthesis of choline formate, employing an inexpensive protocol. Subsequently, attention was directed towards investigating multifunctional heterogeneous catalysts, utilizing coordinated choline cyanides and choline formate on magnetic support. Essentially, an organo-catalyst is stabilized on the magnetic surface, exhibiting Lewis acid and basic characteristics [43, 44]. Initial research focused on the conditions and spectra surrounding the synthesis of choline formate and choline cyanide, followed by coordinating Fe_2_O_3_–MgO as catalysts in Knoevenagel and benzoin condensation reactions.

## Results and discussion

### Catalyst characterization

**Fig. 1:**
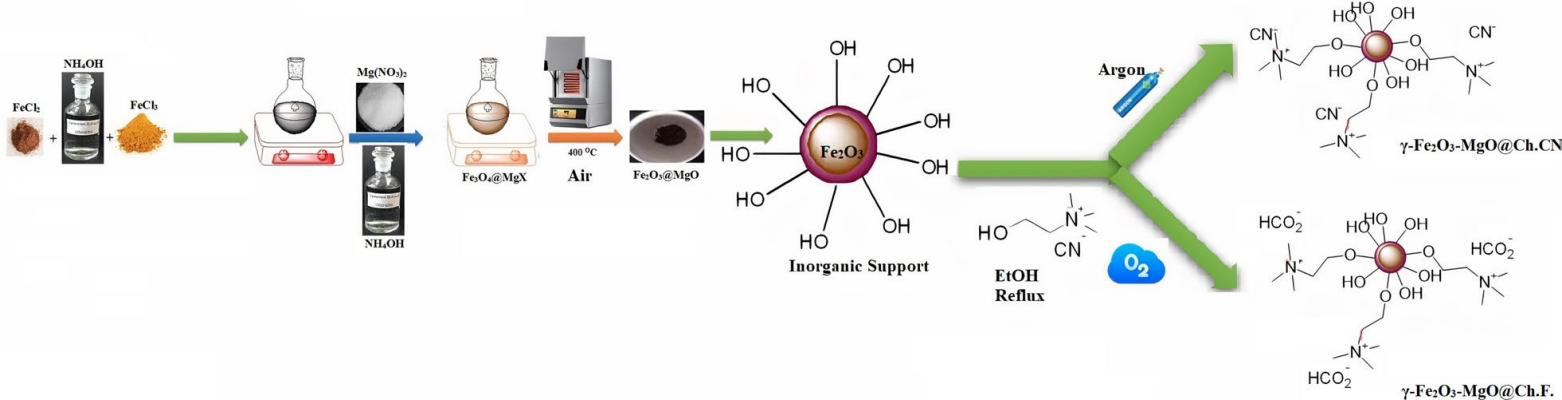
Schematic diagram of catalysts preparation. The source of this diagram is taken from https://www.nature.com/articles/s41598-023-44881-2/figures/3. The software tools employed to create this diagram were Chemdraw and Paint. The figure was designed by authors

Figure 1 depicts the synthesis pathway of γ-Fe_2_O_3_-MgO@Ch.F. and γ-Fe_2_O_3_-MgO@Ch.CN. γ-Fe_2_O_3_ is initially produced as per the literature [41, 44–47]. To fulfill this objective, Fe_3_O_4_ is subjected to a reaction with ammonium hydroxide (27 wt.%) and magnesium nitrate at 70 °C for 12 h. The magnetic powder is subsequently separated and subjected to heating in a furnace for 4 h at 400 °C to yield γ-Fe_2_O_3_-MgO. Moreover, Ch.F. and Ch.CN are synthesized, and their magnetic surfaces are stirred at 25 °C, followed by refluxing in EtOH for 12 h at 80 °C to produce γ-Fe_2_O_3_-MgO@Ch. F. and γ-Fe_2_O_3_-MgO@Ch.CN, respectively. Finally, the catalysts synthesized are characterized through TGA, FTIR, FE-SEM, VSM, EDS, BET, and XRD measurements.

**Fig. 2:**
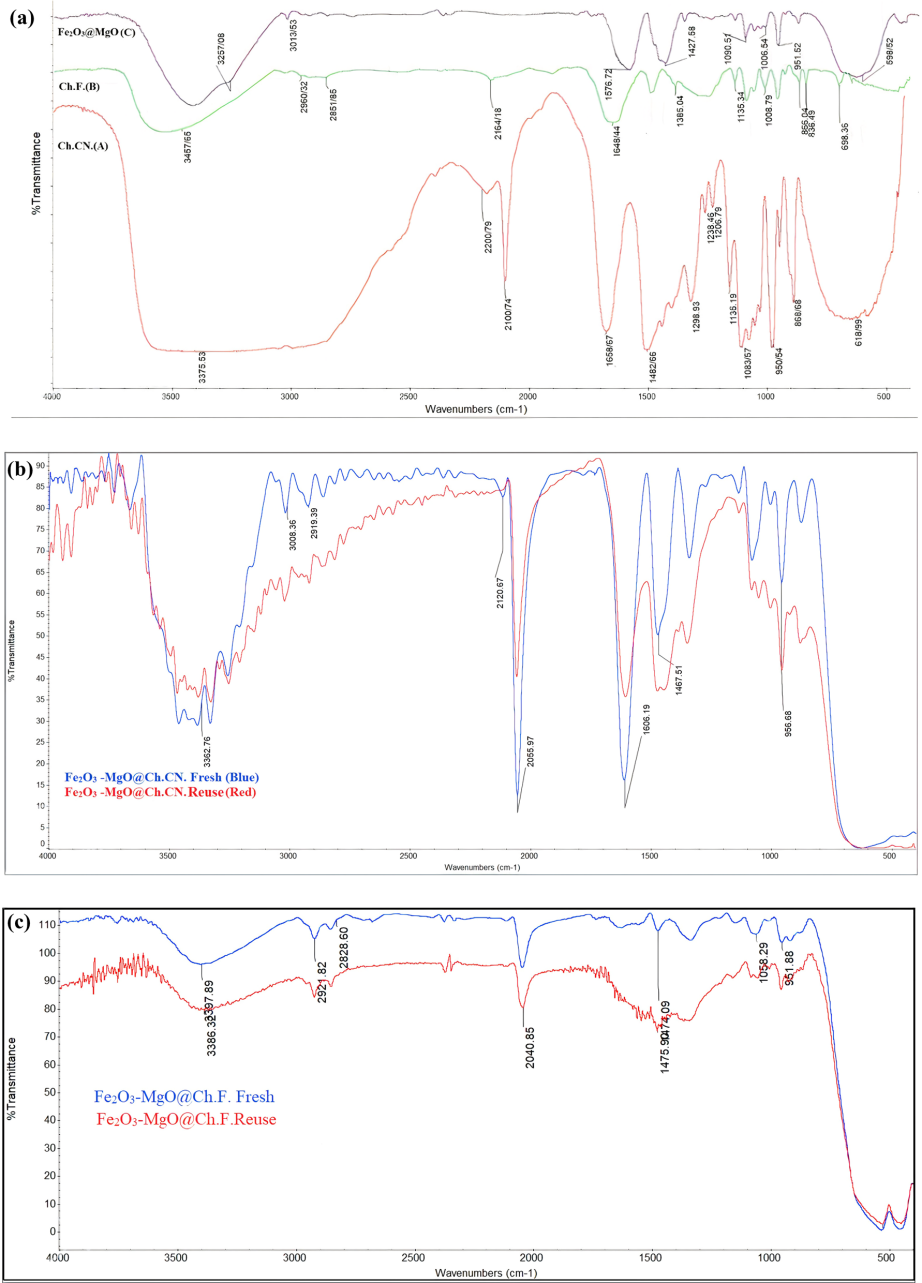
**A** FT-IR spectrum of preparation of Ch.CN (A) Ch.F. (B) γ-Fe_2_O_3_@MgO (C). **B** FT-IR spectrum of preparation of γ-Fe_2_O_3_-MgO@Ch.CN Fresh and Reuse. **C** FT-IR spectrum of preparation of γ-Fe_2_O_3_-MgO@Ch.F. Fresh and Reuse

Figure 2 shows the FT-IR spectrum of Ch.CN, Ch.F., and γ-Fe_2_O_3_-MgO. In Fig. 2A, the FT-IR spectrum of Ch.CN reveals bands at 3375, 2200, 2100, 1482, 1350, and 1083, potentially attributed to OH stretching (3375 cm^−1^), C-H bending (1482cm^−1^), and C–O stretching (1083cm^−1^). Notably, the FT-IR spectrum of Ch.CN exhibits two distinct bands, 2200cm^−1^ (C≡N) and 2100cm^−1^ (N–C), while the N–C peak intensities at Ch.F. are weak (Fig. 2A). Mass spectroscopy and NMR (Additional file 1: Figs. S52–S57) were employed to investigate these proposed structures, with confirmation from sedimentary tests. The FT-IR spectrum of fresh and reused catalysts reveals the Fe–O stretching vibration attributed to the iron oxide in the spinel form, observed around 569 to 584 cm^−1^ (Fig. 2A–C) [48]. In γ-Fe_2_O_3_-MgO@Ch.CN, the FT-IR peak of Ch.CN (2200 and 2100 cm^−1^) shifts to 2120 and 2055 cm^−1^, respectively (Fig. 2A and B). Additionally, the FT-IR peak of Ch.F. (2164 cm^−1^) undergoes a shift to 2040 cm^−1^ (Fig. 2A and C). This shift is attributed to the surface chelation of organic groups to γ-Fe_2_O_3_-MgO.

**Fig. 3:**
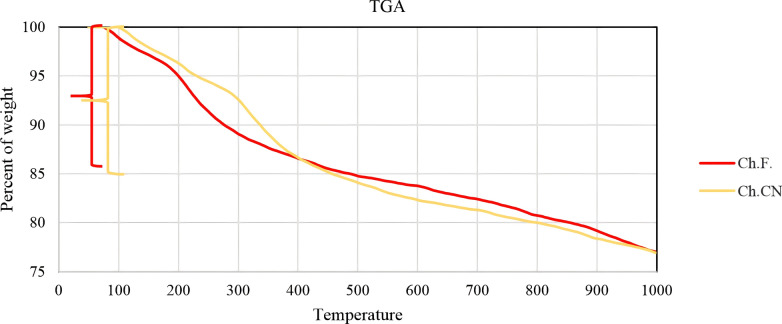
TGA of γ-Fe_2_O_3_-MgO@Ch.F. and γ-Fe_2_O_3_-MgO@Ch.CN

The TGA is employed for the analysis of catalyst composition and heat resistance (Fig. 3). In the cases of γ-Fe_2_O_3_-MgO@Ch.F. and γ-Fe_2_O_3_-MgO@Ch.CN, the TGA curve indicates a minor weight loss of approximately 1–2% at 100 °C, attributed to physically adsorbed water. The decomposition of organic compounds from γ-Fe_2_O_3_-MgO @Ch. F. and γ-Fe_2_O_3_-MgO@Ch.CN leads to a 10–12% weight loss in the temperature range of 200–400 °C. Ultimately, the residual weight of 77% corresponds to Fe_2_O_3_@MgO. Thermal analysis demonstrates that the catalysts exhibit thermal stability up to 200 °C (Fig. 3). Notably, the boiling points of Ch.F. and Ch.CN are 195.38 °C and 190.8 °C, respectively, confirming the non-sensitivity and non-energetic nature of these choline salts. DSC is employed to assess the safety of these innovative compounds in relation to chemical reactions and phase transitions as a function of temperature (Additional file 1: Figs. S1 and S2). Furthermore, no exothermic peak is observed in these experiments, with degradation occurring progressively at approximately 300 °C.

**Fig. 4:**
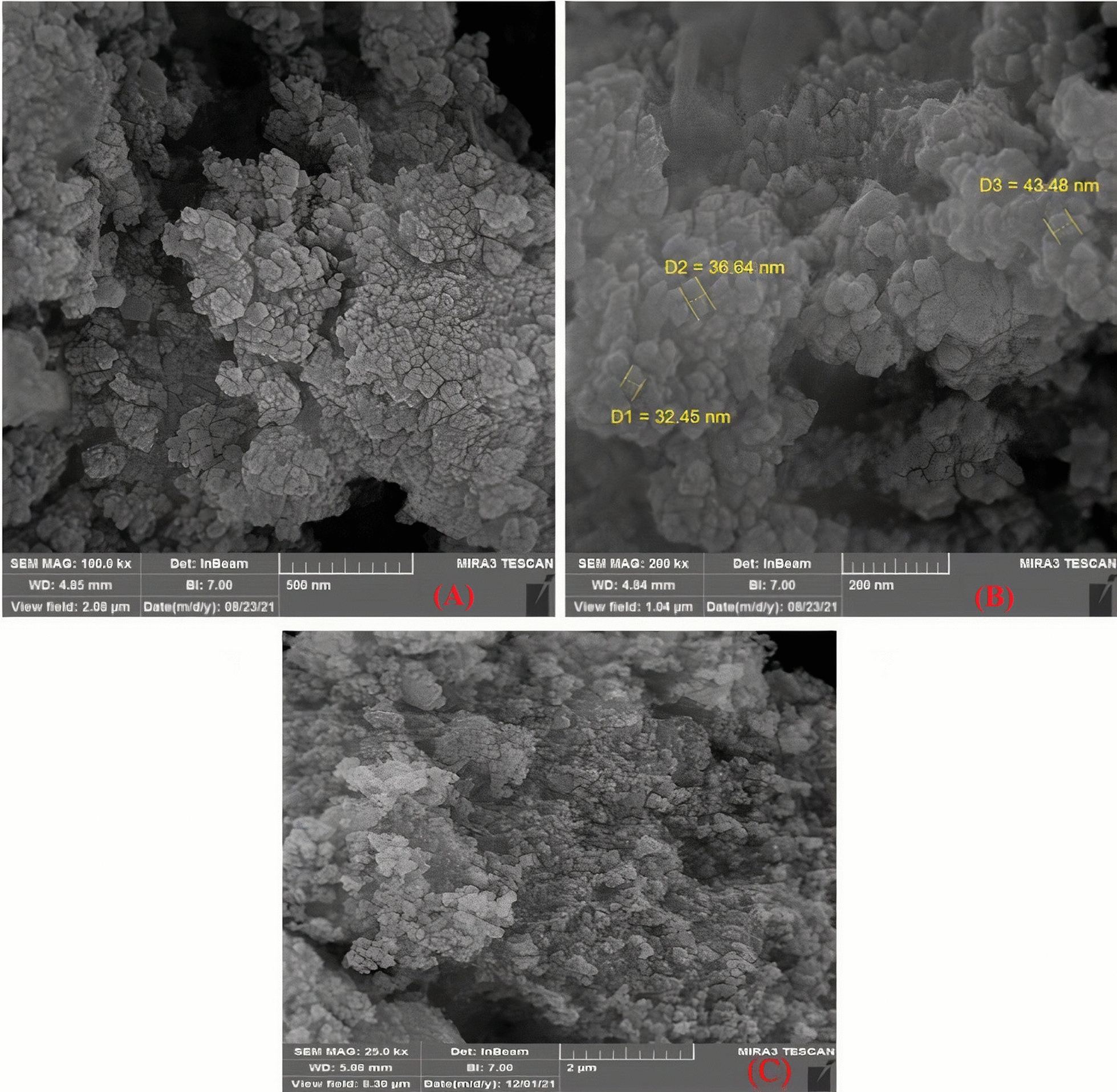
SEM images of γ-Fe_2_O_3_-MgO (**A**), γ- Fe_2_O_3_-MgO@Ch.F. (**B**) γ-Fe_2_O_3_-MgO@Ch.CN (**C**)

**Fig. 5:**
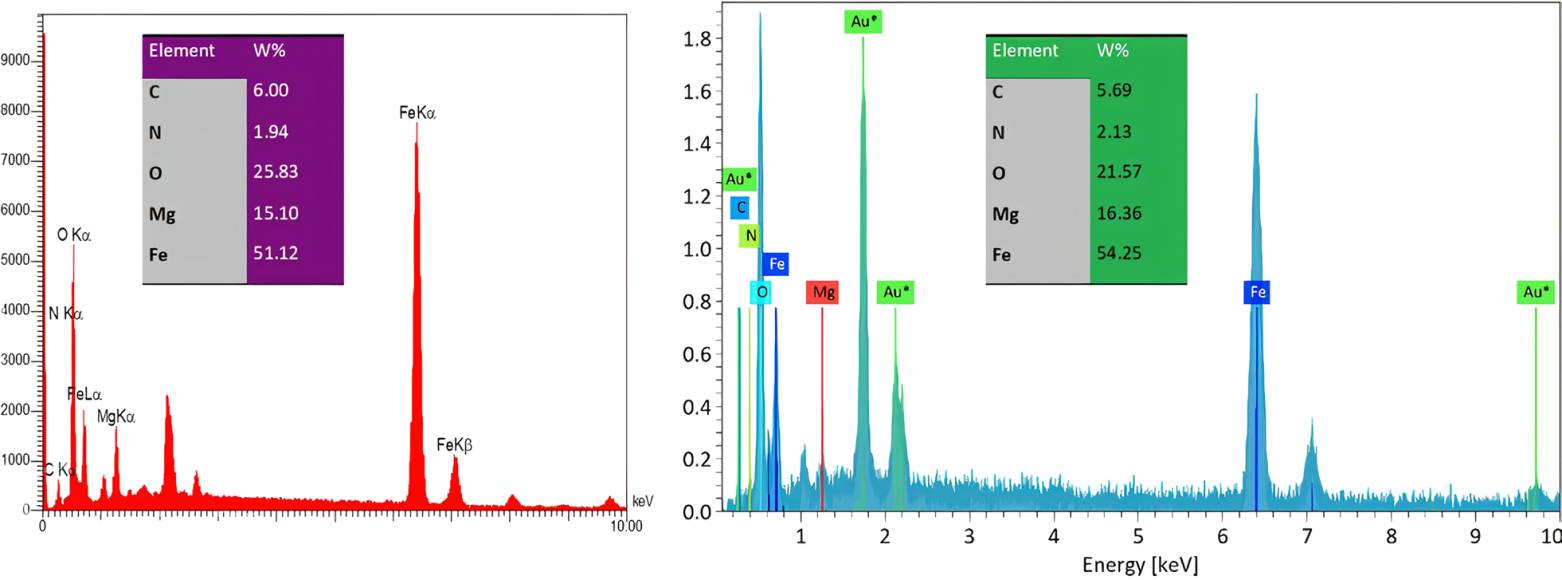
EDS results of Fe_2_O_3_-MgO@Ch.F.(Red) and Fe_2_O_3_-MgO@Ch.CN(Blue)

FE-SEM is employed to investigate the surface morphology of fresh catalysts (Fig. 4). The images reveal the formation of spherical particles with an average size ranging from 32 to 43 nm. EDS analysis is conducted to identify the elements present in the catalysts (Fig. 5). In the case of Fe_2_O_3_-MgO@Ch.X (X: F,CN), the EDS pattern confirms the presence of iron, magnesium, and oxygen, providing credible evidence of the modification of Fe_2_O_3_ by MgO. Alongside these elements, prominent peaks for carbon indicate the successful loading of choline onto Fe_2_O_3_@MgO. The Au peaks observed can be attributed to the sample holder [49].

**Fig. 6:**
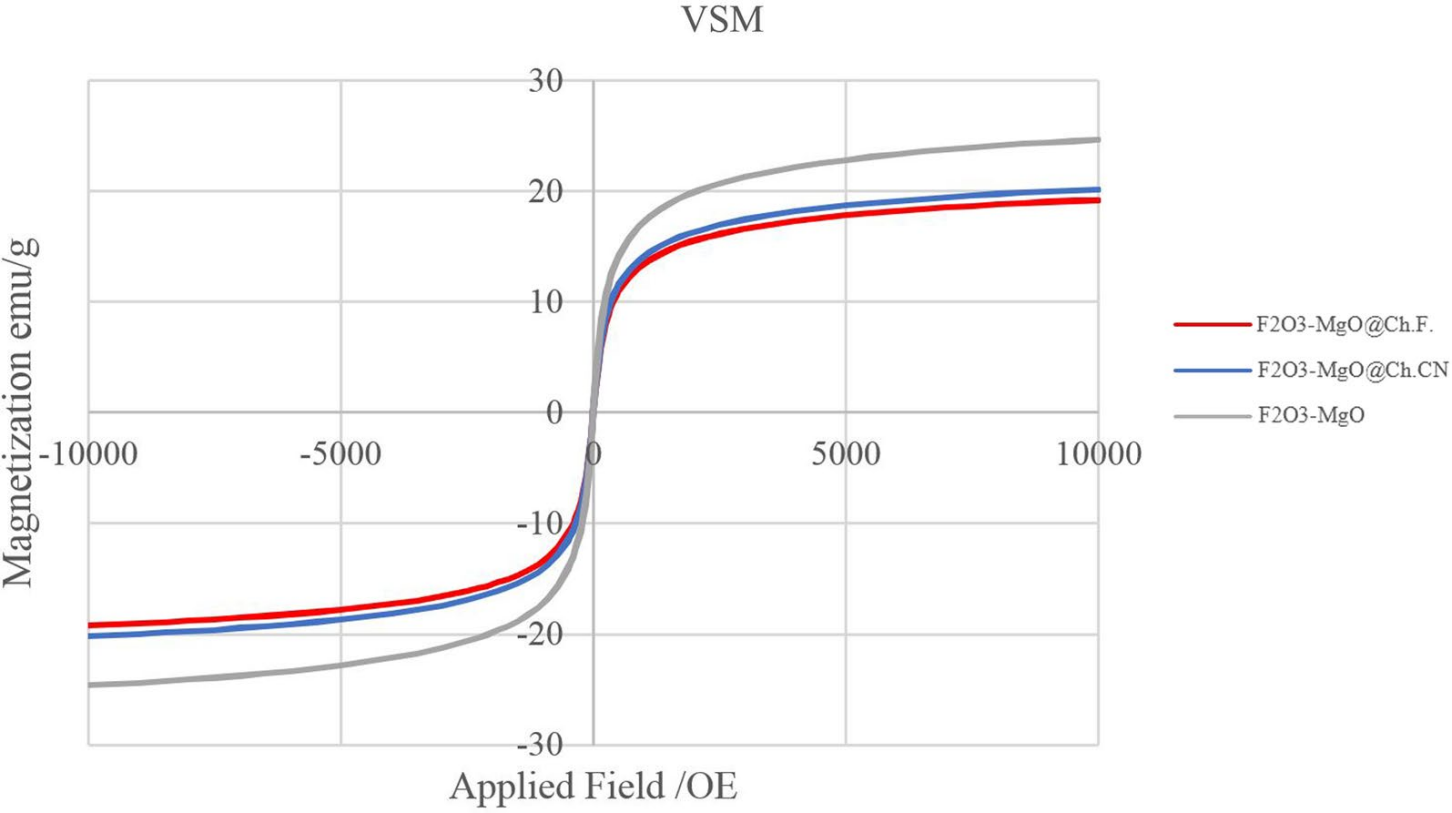
VSM curve of γ-Fe_2_O_3_-MgO(grey), γ-Fe_2_O_3_-MgO@Ch.F.(Red) and γ-Fe_2_O_3_-MgO@Ch.CN(Blue)

The magnetic hysteresis loop readings for γ-Fe_2_O_3_-MgO@Ch.F., γ-Fe_2_O_3_-MgO @Ch.CN and γ-Fe_2_O_3_-MgO are 19, 20, and 25 emu·g^−1^, respectively, indicating saturation magnetization. The observed decrease in saturation magnetization (MS value) may be attributed to the conversion of Fe_3_O_4_ into γ-Fe_2_O_3_ during heating and the coating of MgO on iron oxide [50]. The introduction of Ch.F. and Ch.CN onto the γ-Fe_2_O_3_-MgO surface further diminishes the saturation magnetization value (Fig. 6). The effortless attraction of the nanoparticles to an external magnet further showcased their strong magnetization.

**Fig. 7:**
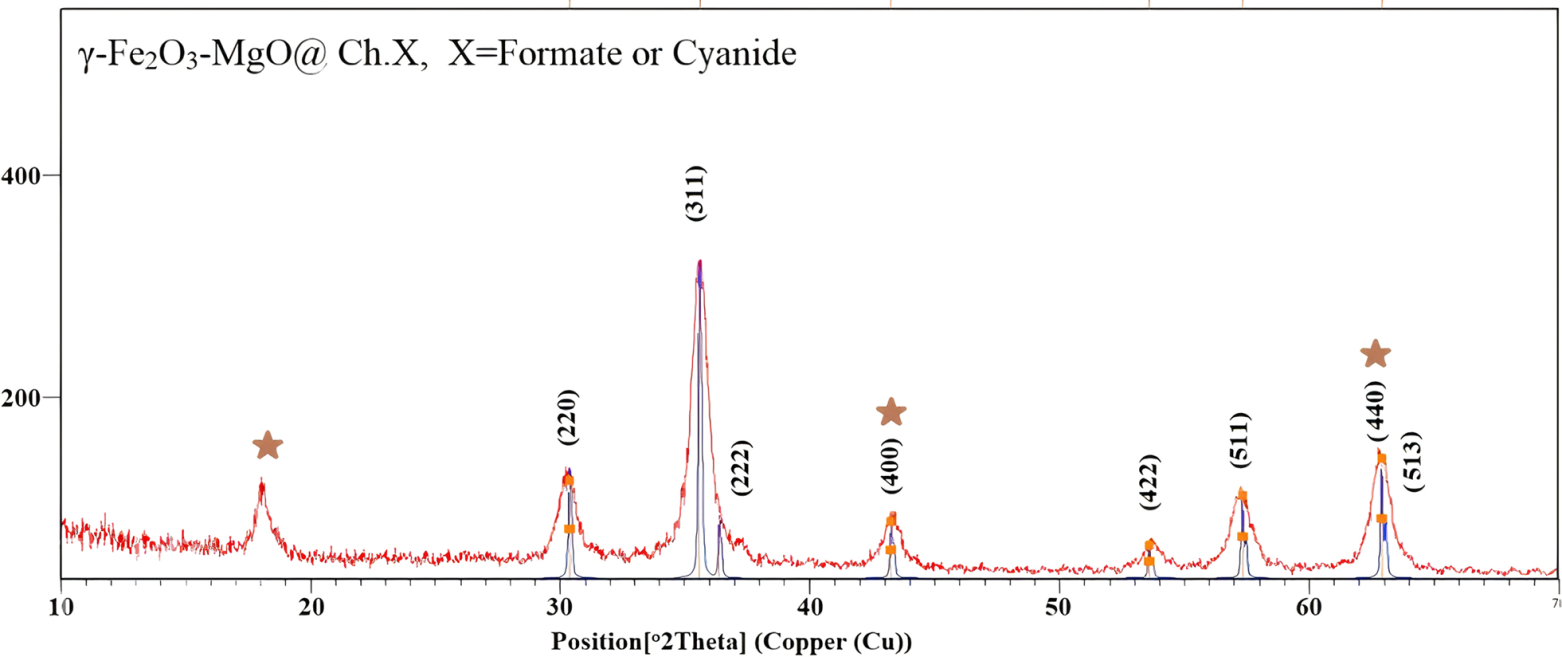
XRD pattern of γ-Fe_2_O_3_-MgO@Ch.F.(Red) and γ-Fe_2_O_3_-MgO@Ch.CN(grey)

The XRD patterns of γ-Fe_2_O_3_-MgO@Ch.F. and γ-Fe_2_O_3_-MgO@Ch.CN are examined within the 10°–80° range to discern their crystalline structures. The XRD patterns of γ-Fe_2_O_3_-MgO@ Ch. F. (red line) and γ-Fe_2_O_3_-MgO@Ch. CN (grey line) (Fig. 7) exhibit minimal variations. Notably, the diffraction peaks at 2Ɵ = 62.7, 62.2, 57.2, 53.8, 42.9, 36.8, 35.6, and 30 in Fig. 7, are consistent with the standard structure of γ-Fe_2_O_3_, as per JCPDS card No. 39-1346. Additionally, peaks at approximately 2Ɵ = 62.2, 50.34, and 18.1 are attributed to MgO (JCPDS 4-829) (Fig. 7) [46].

The specific surface area of the nanocatalysts were determined using the Brunauer–Emmett–Teller (BET) technique (Additional file 1: Fig. S6) [51]. The Fe_2_O_3_-MgO@Ch.CN and Fe_2_O_3_-MgO@ch.F. have BET surface areas of 27.05 and 26.74 m^2^/g, respectively. Incorporating Ch.F. and Ch.CN onto the Fe_2_O_3_-MgO surface leads to a decrease in surface area in comparison to Fe_2_O_3_-MgO [47].

### The catalytic activity

**Fig. 8:**
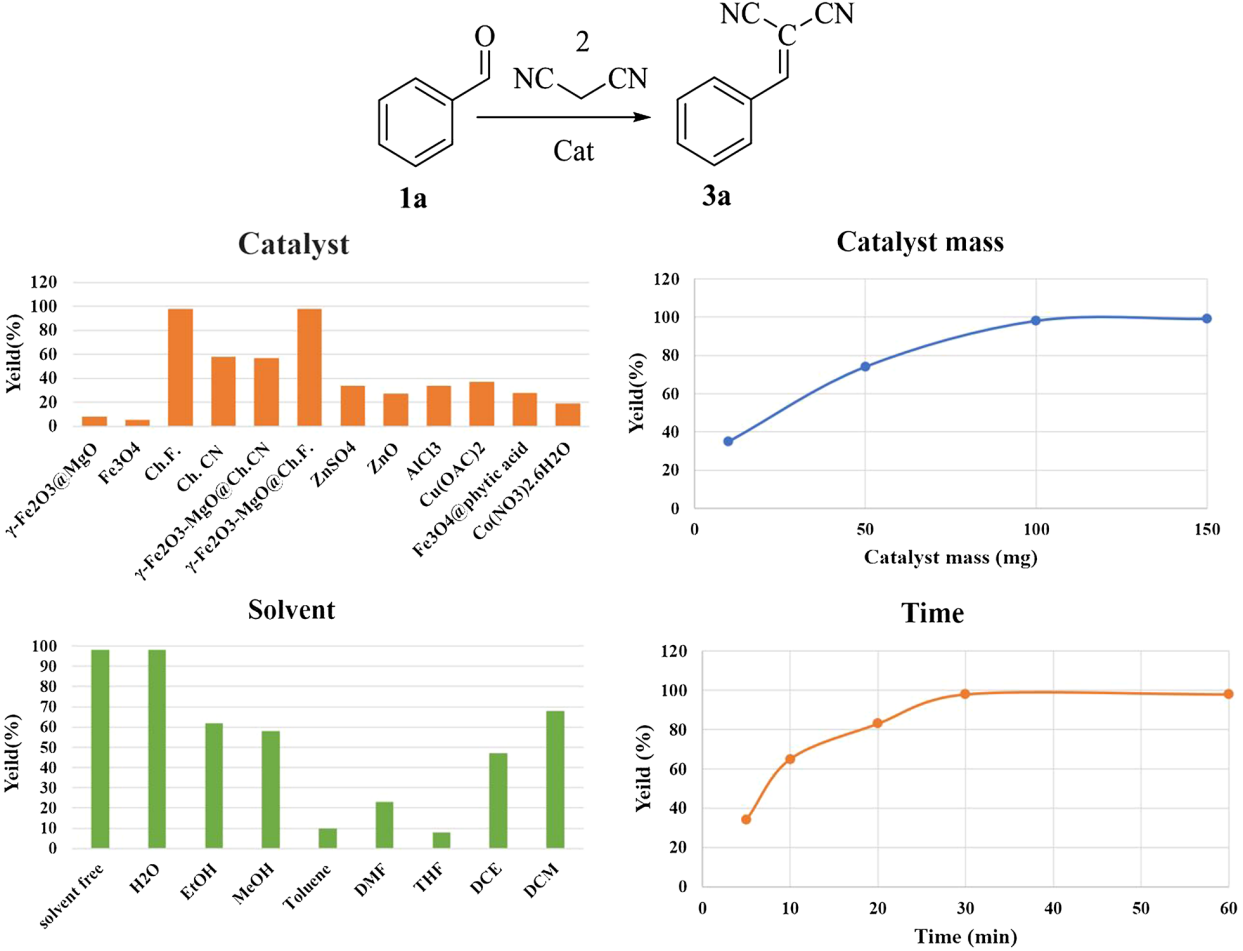
Optimization of Knoevenagel condensation

The catalytic activity of the synthesized and characterized catalysts was evaluated in the Knoevenagel condensation using benzaldehyde 1a and malononitrile 2 as a model reaction in room temperature. Various parameters, including catalyst, solvent, catalyst loading, and reaction time, were investigated [52]. Initially, to identify the primary catalytic center of γ-Fe_2_O_3_-MgO@Ch.X (X: F/CN), a model reaction was conducted in a solvent-free environment for 30 min, employing γ-Fe_2_O_3_-MgO, Ch.F., and Ch.CN as catalysts at room temperature. The results indicated the essential role of an organocatalyst (Ch.F. or Ch.CN) for optimal reaction efficiency. Figure 8 illustrates that the presence of γ-Fe_2_O_3_-MgO@Ch.F. as a heterogeneous catalyst and Ch.F. as a homogeneous catalyst resulted in higher efficiency (98%) in the model reaction. Further comparison with various Lewis acids in solvent-free and room temperature conditions revealed weak to moderate yields, with γ-Fe_2_O_3_-MgO@Ch.F. emerging as the preferred reusable catalyst with superior activity and the highest yield (98%) for future exploration. Exploring different solvents, such as EtOH, H_2_O, MeOH, DCM, DMF, THF, DCE, and toluene, with 100 mg γ-Fe_2_O_3_-MgO@Ch.F. revealed yields of 62, 98, 58, 68, 23, 8, and 47%, respectively. The solvent-free condition, due to its environmental friendliness and higher efficiency (yield 98%), was determined as the optimal choice. Adjusting the catalyst mass and reaction time showed that using 100 mg of γ-Fe_2_O_3_-MgO@Ch.F. and a reaction time of 30 min provided optimal conditions for higher efficiency of the target product (Fig. 8). Therefore, γ-Fe_2_O_3_-MgO@Ch.F., serving as an eco-friendly, reusable, and separable magnetic catalyst demonstrated superior efficiency under solvent-free conditions at room temperature for a short duration. Upon establishing optimal conditions, the scope of the reaction was broadened to include various aromatic and heteroaromatic aldehydes and ketones in both heterogeneous and homogeneous catalysis settings. Table 1 reveals that homogeneous systems often yield non-reusable excellent yields, while γ-Fe_2_O_3_-MgO@Ch.F. consistently produces a reusable, higher output. Notably, several substituted heterocyclic ketones and aldehydes could be efficiently reacted with malononitrile to yield benzylidene malononitrile with high isolated yields (91–98%).

**Table 1:** Screening of the derivatives Knoevenagel condensation

Entry	Structure	Yield(%)* γ-Fe_2_O_3_-MgO@Ch.F	Yield (%)* Ch.F	M.P. (°C)	References
**3a**		98%	98%	82–83	[53]
**3b**		97%	98%	160–162	[53]
**3c**		97%	99%	182	[54]
**3d**		96%	98%	131–134	[53]
**3e**		97%	99%	110–112	[53]
**3f**		99%	99%	100–101	[55]
**3g**		98%	99%	165–166	[56]
**3h**		95%	98%	183–184	[55]
**3i**		95%	98%	73–74	[57]
**3j**		91%	93%	281	[58]
**3k**		92%	95%	244–246	[59]
**3l**		94%	97%	215–217	[60]
**3m**		93%	95%	228–230	[60]
**3n**		93%	96%	245–246	[60]

*Isolated yield, Benzaldehyde (2.5 mmol), malononitrile (2.5 mmol), Cat: Ch.F.:(100 mg (0.67mmol) or γ-Fe_2_O_3_-MgO@Ch.F.:100mg: 0.067mmol)

**Fig. 9:**
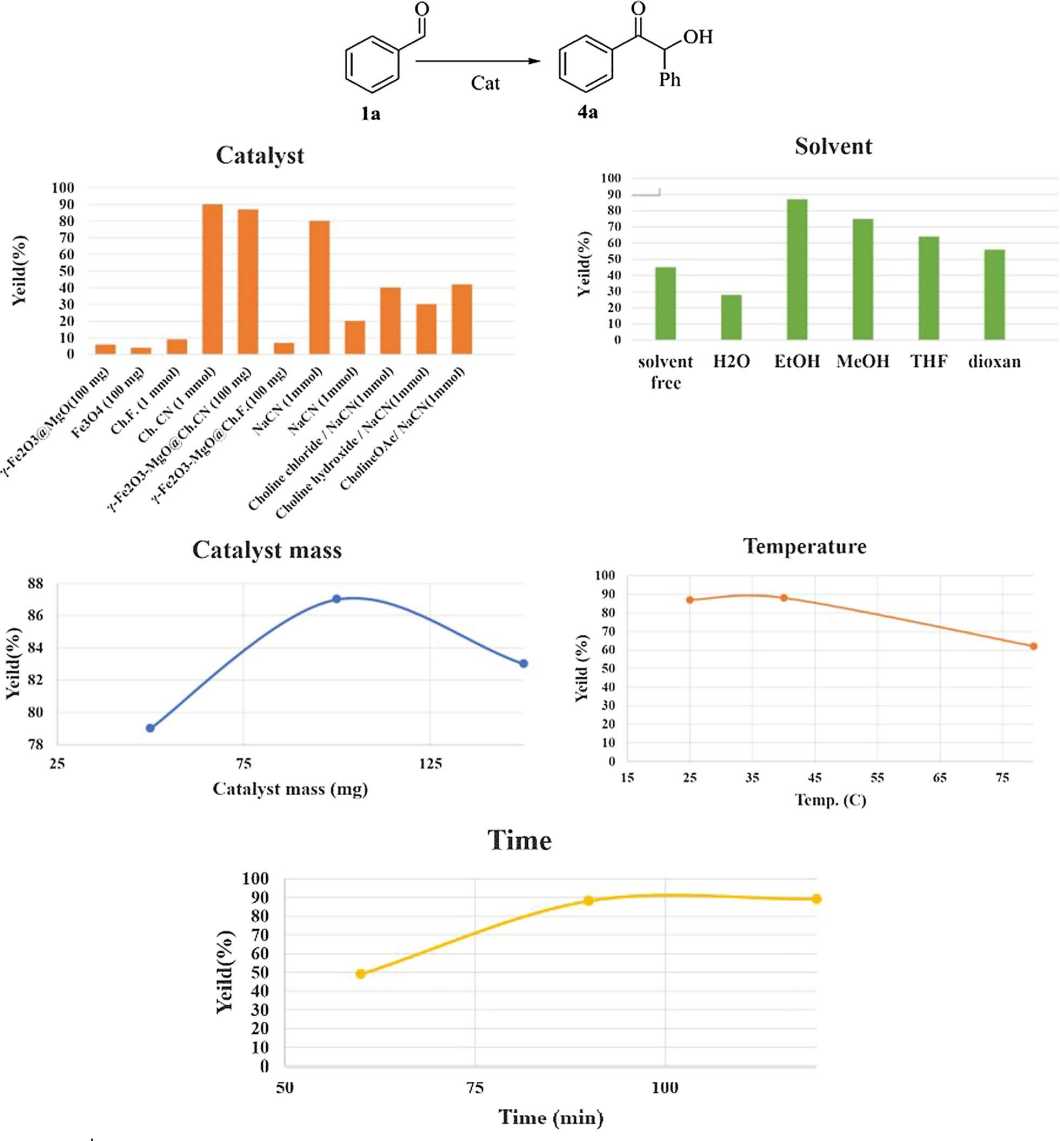
Optimization of benzoin condensation

Encouraged by our success, we sought a new reaction to evaluate chemo-selectivity and explore choline cyanide tendencies, leading us to benzoin condensation. The model reaction involved the reaction of benzaldehyde 1a with itself under room temperature (R.T.) conditions for 1.5 h. Catalysts included Fe_3_O_4_, Fe_2_O_3_-MgO, γ-Fe_2_O_3_-MgO@Ch.F., Ch.F., γ-Fe_2_O_3_-MgO@Ch.CN, and Ch.CN and some NaCN situations were also explored. Consequently, γ-Fe_2_O_3_-MgO@Ch.CN emerged as the preferred reusable catalyst with superior activity (yield 88%) for further investigation (Fig. 9). Additional experiments at different temperatures revealed 25 °C as the optimal reaction temperature. Figure 9 illustrates that the rise in temperature has resulted in a decline in reaction efficiency. This decline can be attributed to the partial conversion of choline cyanide to choline formate. EtOH, H_2_O, MeOH, THF, toluene, and dioxane were investigated as solvents to assess their impact on yield and reaction time. Notably, the performance of γ-Fe_2_O_3_-MgO@Ch.CN improved in the presence of EtOH. As depicted in Fig. 9, employing 100 mg of catalyst and a reaction time of 1.5 h proved to be optimal conditions for achieving higher isolated efficiency (88%) of the target product at room temperature. Therefore, in benzoin condensation, γ-Fe_2_O_3_-MgO@Ch.F. serving as an eco-friendly, reusable, and detachable magnetic catalyst, demonstrated superior isolated efficiency under EtOH as a green solvent at room temperature for a shorter duration.

To assess the breadth of the chemo-selective reaction, various benzyl aldehydes and heteroaromatic compounds with both electron-withdrawing and electron-donating functions were screened under optimum reaction conditions, yielding high isolated efficiency (76–88%) (Table 2). Further details on the proposed methodology can be found in the accompanying material.

**Table 2:** Screening of the derivatives Benzoin condensation

Label	Product	Melting point	References	Yield* Ch.CN (%)	Yield* Cat (%)
4a		132–135 ℃	[61]	90%	88%
4b		65–67 ℃	[61]	84%	82%
4c		94–96 ℃	[61]	83%	80%
4d		76–77 ℃	[62]	81%	79%
4e		109–111 ℃	[61]	80%	80%
4f		134–135 ℃	[63]	81%	76%
4g		107–108 ℃	[64]	81%	78%
4h		239 ℃	[65]	82%	77%
4i		87–89 ℃	[61]	83%	79%

*Isolated yield Benzaldehyde (1 mmol), Cat:choline cyanide (1 mmol:0.13 g) or: Fe_2_O_3_-MgO@Ch.CN:100mg:(0.092mmol)

### Recyclability γ-Fe_2_O_3_-MgO@Ch.F. and Fe_2_O_3_-MgO@Ch.CN

**Fig. 10:**
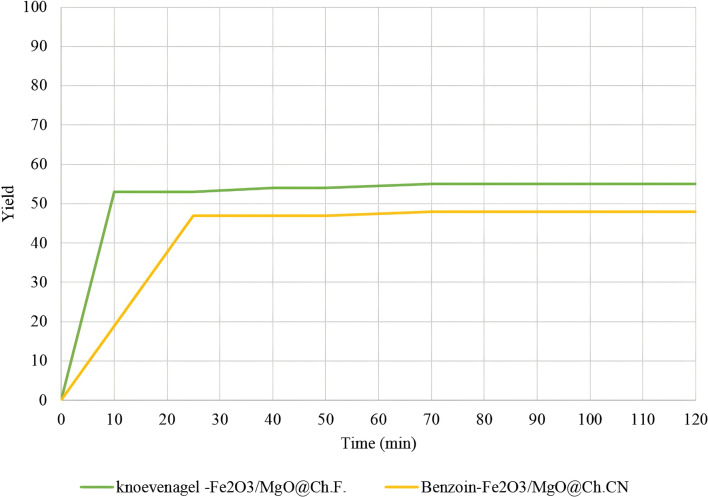
Hot filtration Test

**Fig. 11:**
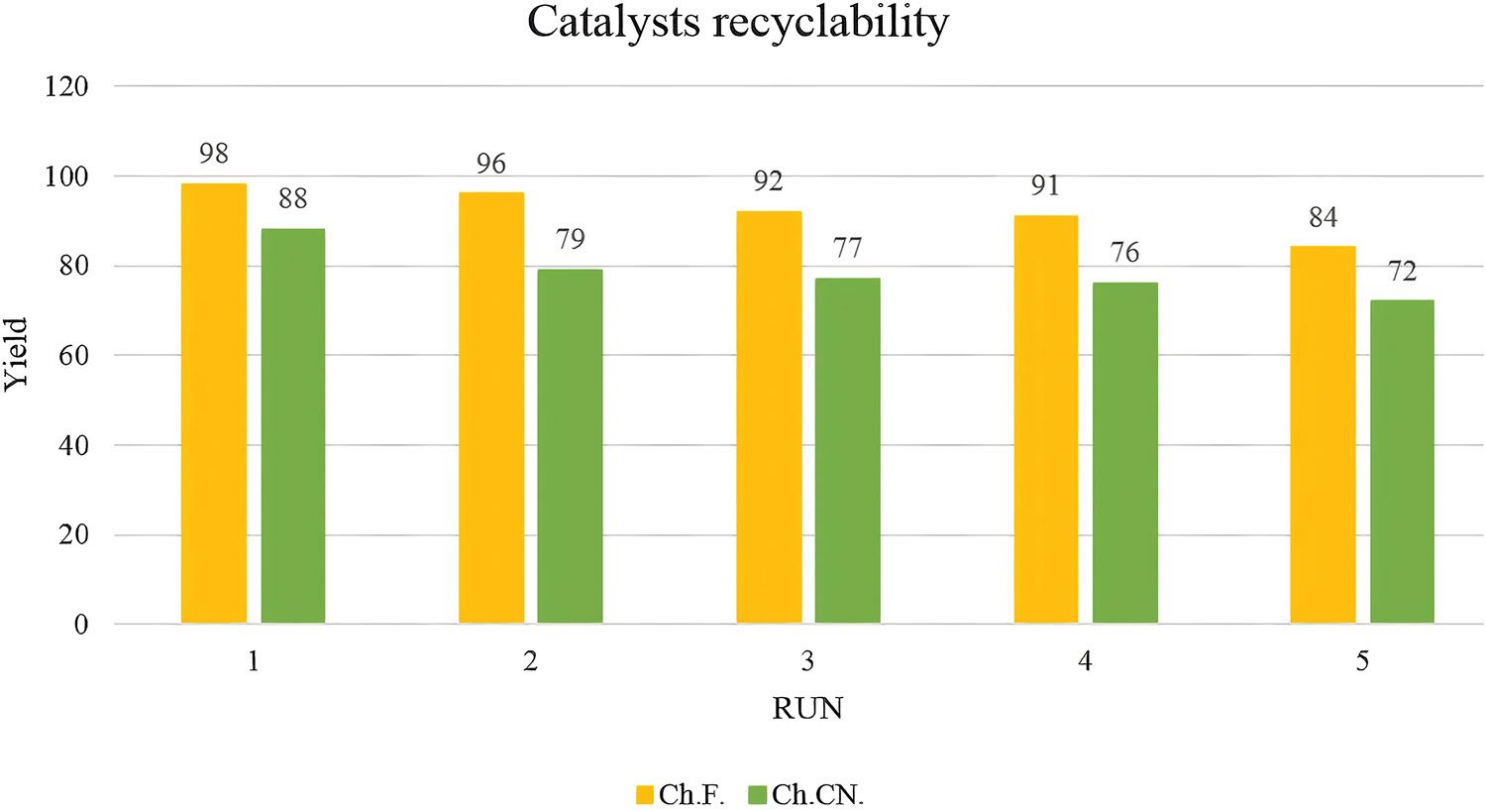
Recyclability of γ-Fe_2_O_3_-MgO@Ch.F. and γ-Fe_2_O_3_-MgO@Ch.CN

Hot filtration tests were conducted under optimal conditions to assess the leaching of Ch.F. from the heterogeneous catalysts during malononitrile and benzaldehyde reactions. Similarly, γ-Fe_2_O_3_-MgO@Ch.CN hot filtration tests were performed under ideal circumstances for benzoin condensation. In these tests, tubes were filled with EtOH after 10 and 25 min, and the catalysts were separated using an external magnet. Subsequently, the catalysts were isolated, and the reactions were terminated. No discernible improvement was observed after 45 min of stirring, as confirmed by GC analysis (Fig. 10). The results suggested that the response predominantly takes place through a heterogeneous pathway. The recyclability of the heterogeneous catalysts was then investigated in model reactions. Both Choline formate and cyanide heterogeneous catalysts could be regenerated for at least five cycles (Fig. 11). Following five runs, the structure of the reused catalysts was examined using FT-IR (Fig. 2B and C), XRD, FE-SEM, and VSM (Additional file 1: Figs. S3–S5). The structure of the reused γ-Fe_2_O_3_-MgO @Ch.F. and γ-Fe_2_O_3_-MgO@Ch.CN remained comparable to that of the fresh catalyst in all analyses.

**Scheme 1:**
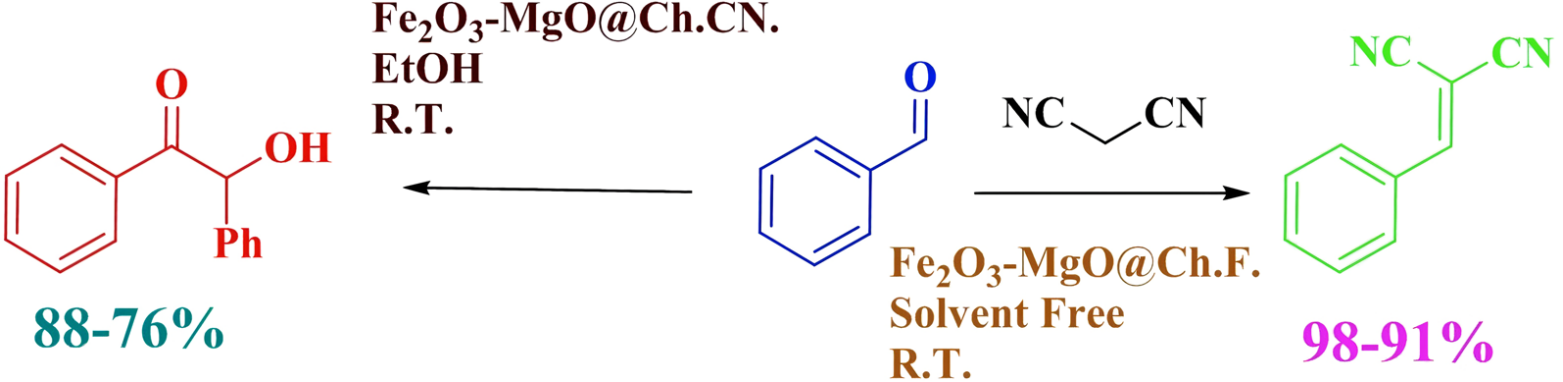
Graphical abstract of condensation reactions using the synthesized nanocatalyst. The source of this diagram is taken from https://encrypted-tbn1.gstatic.com/images?q=tbn:ANd9GcT2p2AUmPXG_97tGU3HEWCr2pLuNGOZ7qSuHqu_ulo_kR632V75. The software tool employed to create this diagram was Chemdraw. The Scheme was designed by authors

Table 3 provides a comparative analysis of the performance of two heterogeneous catalysts employing the solvent-free condensation approach, in contrast to methodologies reported in the literature. The results highlight the remarkable efficiency of γ-Fe_2_O_3_-MgO@Ch.F. and γ-Fe_2_O_3_-MgO@Ch.CN in catalyzing condensation reactions. Notably, these catalysts address certain drawbacks associated with previously reported methods. In comparison to many earlier catalysts, the presented catalysts offer distinct advantages. They prevent the need for homogeneous catalysts, which are challenging to separate from the reaction mixture, more costly, involve hazardous organic solvents, and necessitate extended reaction times. In contrast, the protocol outlined in this study offers several benefits, including reusability, cost-effectiveness, simple preparation using readily available materials, easy separation through an external magnetic field, and the ability to achieve good to high yields within a short reaction time. Furthermore, these catalysts operate under mild and environmentally friendly conditions, establishing their superiority over previously reported counterparts. Scheme 1 illustrated a graphical depiction of the condensation reactions aided by the synthesized nanocatalyst.

**Table 3:** Compares the efficiency of various methods for solvent-free condensation

Entry	Time	Mol of cat	cat	Temperature	Yield	TON	TOF	Type of condensation	Reference
1	2 h	0.01 mol	Ammonium bicarbonate	90 °C	91%	1.365	0.068	K.C	[66]
2	1 h	0.005 mol	(1-((4-chlorophenyl) amino)-1- oxopropan-2-aminium perchlorate)	25 °C	97%	0.0194	0.0194	K.C	[67]
3	3 h	0.000006 mol	Zn(pcp)(aip). (H_2_O)	25 °C	99%	32.9	10.96	K.C	[68]
4	1 h	0.2 mol	1-alkyl-2,3 dimethylimida olium salts (NHC) and DBU(0.2)	80 ^o^C	72%	0.0036	0.0036	B.C	[69]
5	12 h	0.5 mol	Imidazolium salts(NHC)/Cs_2_CO_3_	30 ^o^C	95%	0.00475	0.0004	B.C	[70]
6	5 min	0.02 mol	BMICl/NaOMe	150 °C/microwave	97%	0.53	13.2	B.C	[71]
8	30 min	0.067 mmol	γ-Fe_2_O_3_-MgO@Ch.F	25 °C	98%	36.56	73.13	K.C	This work
9	1.5 h	0.092 mmol	γ-Fe_2_O_3_-MgO@Ch.CN	25 °C	88%	4.78	3.18	B.C	This work

K.C.: Knoevenagel condensation; B.C.: Benzoin condensation

**Scheme 2:**
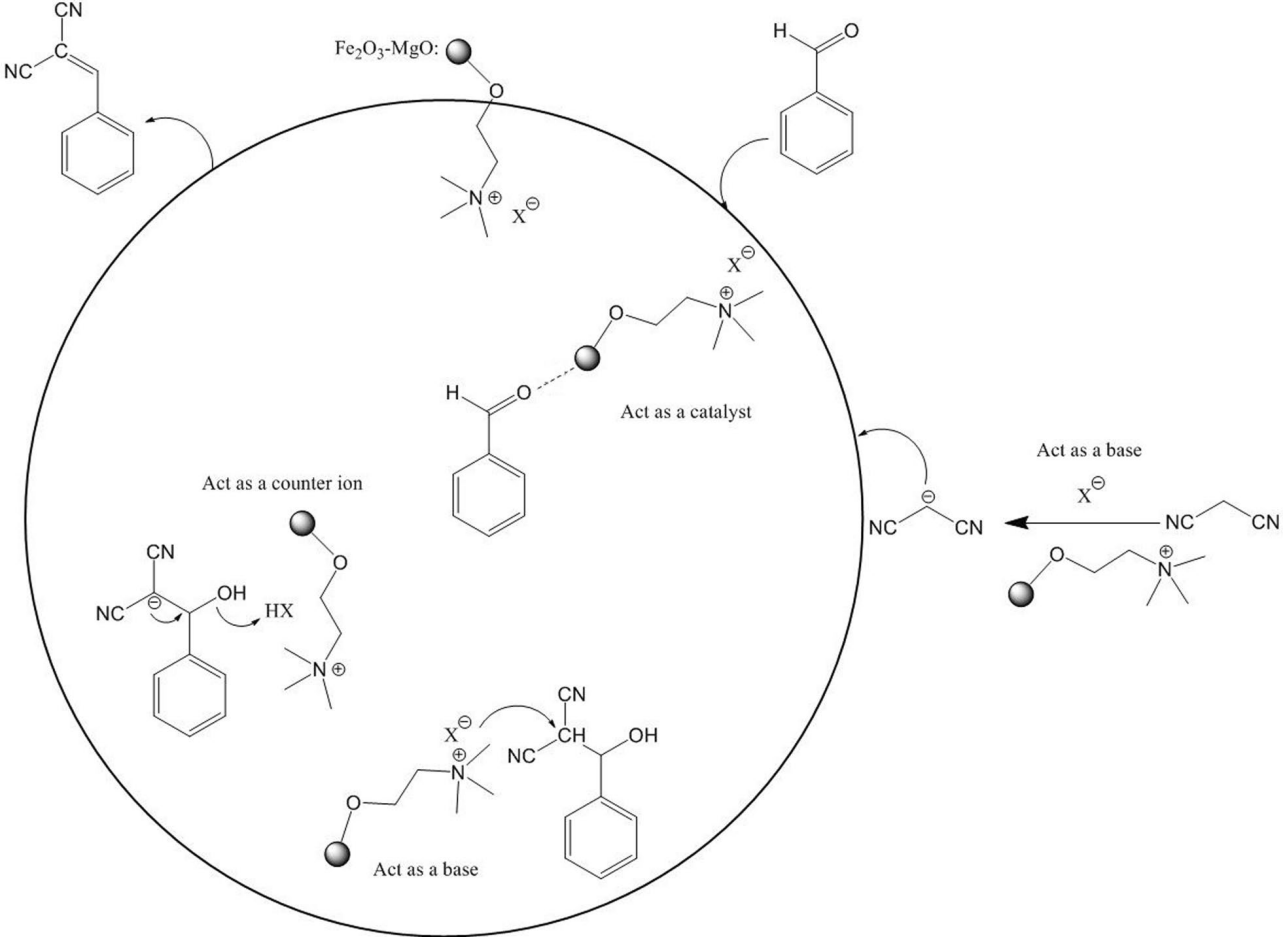
Proposed reaction mechanism for Knoevenagel condensation

**Scheme 3:**
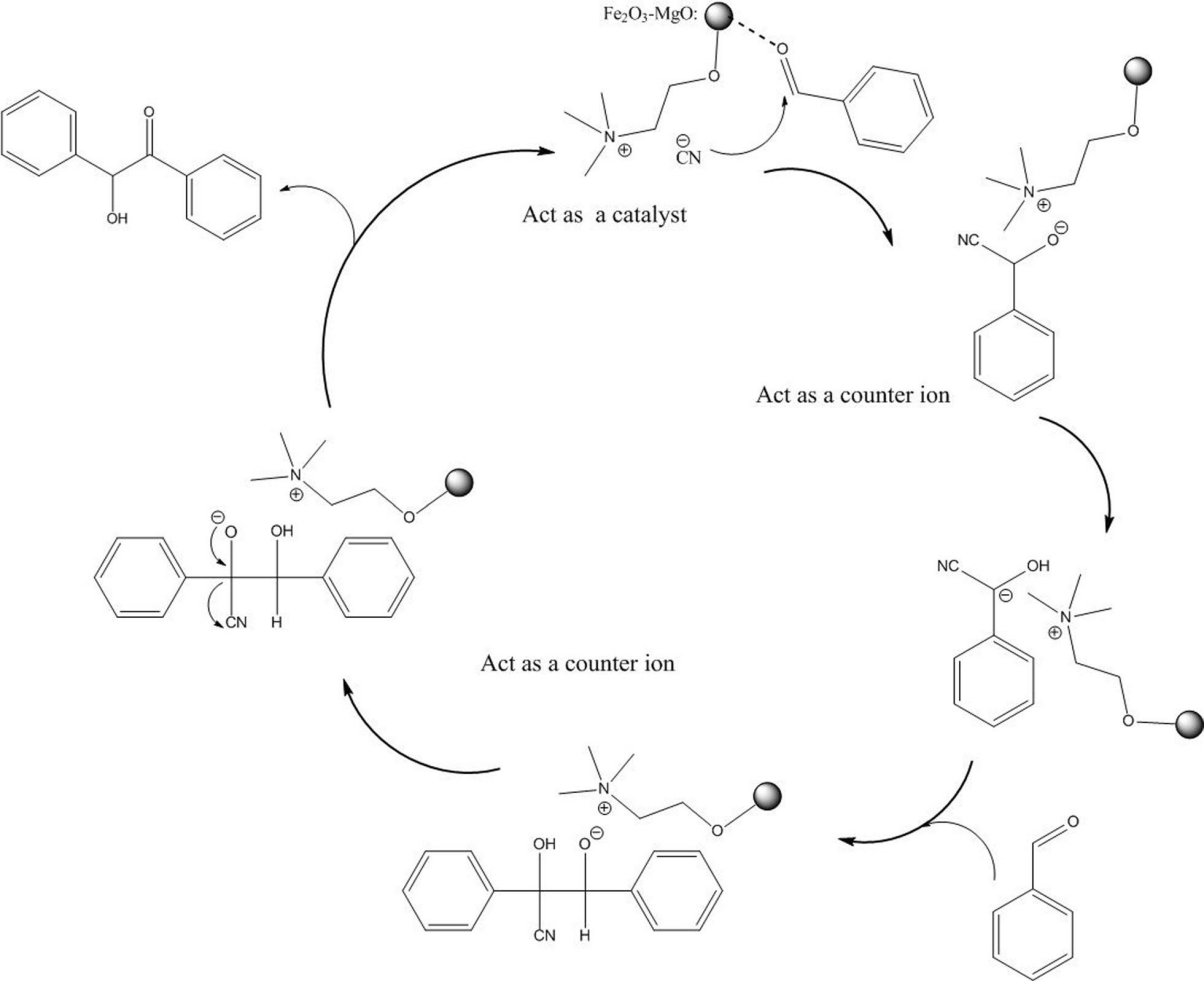
Proposed mechanism for benzoin condensation

Schemes 2 and 3 depicts the proposed mechanisms for the Knoevenagel and benzoin condensations of aromatic aldehydes catalyzed by Fe_2_O_3_-MgO@cholin X (X:CN, F.) nanoparticles, respectively. In the Knoevenagel reaction, the nanocatalyst activates the aromatic aldehyde, initiating a nucleophilic attack by the methylene group of malononitrile. This sequence leads to the formation of a carbon–carbon bond, followed by the dehydration of the intermediate to yield the Knoevenagel product [72]. For the benzoin condensation, the product is synthesized through a classical mechanism [73]. The activated aromatic aldehyde undergoes nucleophilic addition with a cyanide ion, followed by the addition of a carbanion to another molecule of aldehyde. The elimination of the cyanide ion then produces benzoin as the final product. In both reactions, the nanomagnetic support with Lewis and basic sites activates the aromatic aldehydes and stabilizes the carbanion intermediates. The choline cation forms an ion pair with the organic moiety carrying a negative charge. The immobilization of the choline base ion liquid onto the magnetic support enhances the catalyst's reusability, making it an attractive and efficient option for these types of reactions.

## Conclusions

This study has successfully developed and synthesized novel super-paramagnetic multifunctional nanocatalysts. The process involved a straightforward procedure using inexpensive and readily available materials, focusing on the immobilization of homogeneous organocatalysts on magnetic support. Characterization of these nanoparticles was conducted through various techniques, including FT-IR, TGA, FE-SEM, VSM, EDS, BET, and XRD. The optimization of reaction conditions in benzoin condensations resulted in a remarkable yield of 91–98% under solvent-free and room-temperature Knoevenagel reaction conditions. Additionally, a yield of 76–88% was achieved in EtOH at 25 °C for 1.5 h in benzoin condensation, using 100 mg of the catalyst. The key advantages of this protocol encompass reusability, easy separation facilitated by an external magnetic field, and high yield compared to similar homogeneous catalysts. Results from hot filtration tests, VSM, XRD, and SEM investigations indicate that these catalysts can be effectively reused up to five times without significant loss in efficiency. Moreover, this methodology has demonstrated effectiveness across a broad spectrum of aromatic and heteroaromatic aldehydes in both Knoevenagel and benzoin condensations.

## Experimental

### General

The chemicals were purchased from Merck, Aldrich, or Fluka without further purification. A BRUKERDRX-4F00AVANCE Advance spectrometer was used to record the NMR spectra. Electrothermal 9100 apparatus was used to measure melting points uncorrected. Nicolet IR100 instrument recorded IR spectra over a range of 400–4000 cm^−1^ with spectroscopic grade KBr. Vibrating magnetometers/alternating gradient force magnetometers (MD Co., Iran, www.mdk-magnetic.com) were used for the magnetic measurement experiments. Diffraction pattern of the sample was determined using a Philips X‐Pert 1710 diffraction meter. A spectrum of energy-dispersive X-rays (EDX) and field emission scanning electron microscopy (FESEM). Images were recorded on Tescan MIRA3 FE-SEM. A BET analysis was conducted to ascertain the specific surface area of the composite that was prepared, utilizing the Micromeritics Instrument Corporation/TriStar II device. TGA measurements were performed on the Simultaneous Thermal Analyzer (STA 504) (www.tainstruments.com). The Mettler Toledo DSC 1 analyzer was used to carry out differential scanning calorimetry tests on choline salts.

### Preparation of choline cyanide

In dry-methanol (500 mL), choline chloride (1 mol) and sodium cyanide (1 mol) were refluxed for 6 h under inert condition. By ion exchange, Choline cyanide was obtained by evaporating the methanol solution under reduced pressure and filtering the sodium salts (NaCl, extra NaCN). An orange-liquid was formed.

### Preparation of choline formate

In 500 mL methanol, choline chloride (1 mol) and sodium cyanide (1 mol) were refluxed for 6 h under air condition. By ion exchange, Choline formate was obtained by evaporating the methanol solution under reduced pressure and filtering the sodium salts (NaCl, extra NaCN). A red-liquid was formed.

### Preparation of Fe_2_O_3_@MgO

In order to prepare Fe_3_O_4_ nanoparticles, 100 mL of 10 mmol FeCl_3_·6H_2_O in 5 mmol FeCl_2_·4H_2_O in aqueous solutions were heated to 85 °C. A drop-wise addition of ammonia (20 mL 27 weight %) was then added under stirring to reach a pH of 10–11. Following 1 h of stirring at room temperature, the black dispersion was heated to reflux for 1 h. Using an external magnet, the brown precipitate was separated. The effluent solution pH was neutralized by washing with deionized water several times. Additionally, ethanol was used to wash and suspend the particles. Magnetite nanoparticle suspensions were sonicated with an excess of water (1:20) and magnesium nitrate or magnesium chloride (5 mmol). Under sonication for 1 h, the reaction mixture was heated, then stirred for 12 h at 70 °C under stirring. The magnet was used to separate the particles, and the ethanol was used to wash them. Afterwards, the powder was calcined at 400 °C for 4 h in air to yield γ-Fe_2_O_3_@MgO.

### Preparation of Fe_2_O_3_-MgO@Ch.X

0.5 g Fe_2_O_3_-MgO, dry EtOH (4 mL), and choline formate (under air) or choline cyanide (under argon) (5 mmol) were stirred at 25 °C for 15 min. It was then stirred at 80 °C for 12 h. Burnt brown–red catalyst was separated by potent magnet decantation. After washing with EtOH and acetone, the catalyst was dried at 50 °C for 6 h.

### Solvent-free Knoevenagel condensation procedure

A mixture of catalyst (100 mg), aldehyde (2.5 mmol), malononitrile (2.5 mmol), were stirred at 25 °C. After completion of the reaction (TLC), the magnetic catalyst was separated by an external magnet. The reaction solution was diluted with ethyl acetate (2 × 2 mL). The organic layer was separated and then concentrated under reduced pressure. The pure product was obtained by recrystallization with ethyl acetate: *n*-Hexane.

### The general procedure for benzoin condensation

An EtOH solution was stirred with catalyst (100 mg), and aldehydes (1 mmol) at 25 °C. After the reaction (TLC), ethyl acetate was used to extract the product (2 × 2 mL). Separated organic layers were concentrated under reduced pressure. A pure product was obtained by n-hexane and ethyl acetate as solvent: anti solvent.

### Supplementary Information

**Additional file 1:** Supplementary Material 1.

## Data Availability

The data that supports the findings of this study are available in the supplementary material of this article. Also, All of Crude data are available at: https://zenodo.org/record/7753834#.ZBiganZBxPY (10.5281/zenodo.7753834).
