# Supplementary material for: Modified nano magnetic Fe2O3-MgO as a high active multifunctional heterogeneous catalyst for environmentally beneficial carbon–carbon synthesis
Source: BMC Chem. 2024 Apr 20;18(1):78. doi: 10.1186/s13065-024-01176-5 (PMC11032600; doi:10.1186/s13065-024-01176-5)
Supplement: Supplementary file 1 — Additional file 1: Supplementary Material 1. [file 13065_2024_1176_MOESM1_ESM.docx]

**Additional file for**

**Modified nano magnetic Fe_2_O_3_-MgO as a high active multifunctional heterogeneous catalyst for environmentally beneficial carbon-carbon synthesis**

**Ehsan Kamali , Fahim Dreekvandy Abolfazl Mohammadkhani, Akbar Heydari*.**

^1^Chemistry Department, Tarbiat Modares University, Tehran, PO Box:14155-4838, Iran

E-mail: [Heydar_a@modares.ac.ir](mailto:Heydar_a@modares.ac.ir) , [Ehsan.kamali@modares.ac.ir](mailto:Ehsan.kamali@modares.ac.ir), [M_abolfazl@modares.ac.ir](mailto:M_abolfazl@modares.ac.ir), [F.dreekvandy@modares.ac.ir](mailto:F.dreekvandy@modares.ac.ir)

*Characterization of Products*

The compounds 3a, 3b, 3c, 3d, 3e, 3f, 3g, 3h, 3i, 3g_,_ 3k_,_ 3l,3m, 3n, 4a,4b,4c,4d,4e,4f,4g,4h are known compounds and were reported previously. 5a and 5b are reported at the first time .

2-benzylidenemalononitrile (3a) M.P. 82 (Lit[1]. M.P. : 82-83 ºC) ^1^H NMR (400 MHz, CDCl_3_) δ 7.98 – 7.90 (m, 2H), 7.81 (s, 1H), 7.70 – 7.62 (m, 1H), 7.61 – 7.53 (m, 2H). IR(KBr): 3234, 3031, 2223,1637, 1591, 1217 cm^-1^

 2-(4-chlorobenzylidene)malononitrile (3b) M.P.: 162-163 ᵒC (Lit. [1] M.P. 162-163ºC). ^1^H NMR (400 MHz, DMSO) δ 8.54 (s, 1H), 7.99 – 7.91 (m, 2H), 7.75 – 7.67 (m, 2H). Molecular weight:188, Molecular weight found :188.2 IR(KBr): 3240 , 2986, 2229, 1638, 1448, 1048, 973, 847, 786, cm^-1^

2-(4-(dimethylamino)benzylidene)malononitrile(3c) M.P. 182(Lit[2]. M.P. 182ºC) ^1^H NMR (400 MHz, CDCl_3_) δ 7.88 – 7.80 (m, 2H), 7.49 (s, 1H), 6.75 – 6.67 (m, 2H), 3.17 (s, 6H). IR(KBr): 3234,2921,2209,1637,1616,1385 cm^-1^

2-(4-methylbenzylidene)malononitrile (3d) M.P. 131-134 (Lit[1]. M.P. 136-137ºC) ^1^H

NMR (400 MHz, CDCl_3_) δ 7.87 – 7.81 (m, 2H), 7.75 (s, 1H), 7.38 – 7.33 (m, 2H), 2.48 (s, 3H). IR(KBr): 3232,2918,2223,1637,1617,1588,1384,1191 cm^-1^

 2-(4-methoxybenzylidene)malononitrile (3e) M.P.: 110-112ᵒC (Lit. [1]. M.P. 113ºC) ^1^H NMR (400 MHz, CDCl_3_) δ 7.98 – 7.90 (m, 2H), 7.68 (s, 1H), 7.08 – 7.00 (m, 2H), 3.94 (s, 3H). IR(KBr): 3230, 3027, 2923, 2222,1627,1606,1571,1384,1277,1021cm^-1^

 2-(3-nitrobenzylidene)malononitrile (3f) M.P. 100-101 (Lit[3]. M.P. 99-101 ºC) ^1^H NMR (400 MHz, CDCl_3_) δ 8.69 (t, *J* = 2.0 Hz, 1H), 8.50 (ddd, *J* = 8.3, 2.2, 1.0 Hz, 1H), 8.39 – 8.31 (m, 1H), 7.93 (s, 1H), 7.82 (t, *J* = 8.1 Hz, 1H).IR(KBr): 3083,2225,1596,1355,1215 cm^-1^

 2-(4-bromobenzylidene)malononitrile (3g), M.P. 165-166 (Lit. M.P. 162-163[4] ºC) ^1^H NMR (400 MHz, CDCl_3_) δ 7.83 – 7.77 (m, 2H), 7.74 (s, 1H), 7.73 – 7.66 (m, 2H). IR(KBr): 3027,2225,1637,1578,822 cm^-1^

2-(4-hydroxybenzylidene)malononitrile (3h), M.P. 183-184(Lit. [3] M.P. 186 ºC) ^1^H NMR (400 MHz, CDCl_3_) δ 7.88 – 7.77 (m, 2H), 7.56 (s, 1H), 6.89 – 6.75 (m, 2H), 5.58 (s, 1H). IR(KBr): 3413, 3236, 3036, 2917, 2220, 1638,1616,1558,1384,1161cm^-1^

2-(2-hydroxybenzylidene)malononitrile (3i) M.P. =73-74(Lit. [5] M.P. 163-164 ºC) ^1^H NMR (400 MHz, CDCl_3_) δ 7.79 (s, 1H), 7.55 (ddd, *J* = 8.6, 7.4, 1.6 Hz, 1H), 7.41 (dd, *J* = 7.7, 1.7 Hz, 1H), 7.24 (td, *J* = 7.6, 1.1 Hz, 1H), 7.18 (d, *J* = 8.3 Hz, 1H). IR(KBr):3294,3024,2917,2847,2231,1653,1610,1261,1041. cm^-1^

2-(1,3-dioxo-1,3-dihydro-2H-inden-2-ylidene)malononitrile (3j) M.P. =281(Lit. [6] M.P. 281ºC) ^1^H NMR (400 MHz, DMSO) δ 8.11 – 7.89 (m, 4H). IR(KBr): 3240, 2918 , 2104 ,1750 ,1712 ,1678 ,1637 ,1616 ,1421, 1222,1093 cm^-1^

 2-(2-oxoacenaphthylen-1(2H)-ylidene)malononitrile (3k) M.P. =244-246(Lit. [7]M.P. 244-246ºC) 1H NMR (400 MHz, CDCl3) δ 8.60 (d, *J* = 7.3 Hz, 1H), 8.34 – 8.29 (m, 2H), 8.20 (d, *J* = 7.0 Hz, 1H), 7.94 – 7.87 (m, 2H). IR(KBr): 3238, 2920,2228, 1719, 1637,166,1420,cm^-1^

2-(2-oxoindolin-3-ylidene)malononitrile (3l) M.P. =215-217(Lit. [8] M.P. 215-217ºC) IR(KBr-ethyl acetate ): 3269,3111,2876,2233,1736,1718,1625,1589,cm^-1^

2-(5-bromo-2-oxoindolin-3-ylidene)malononitrile (3m): M.P. . =228-230(Lit. [9]M.P. 228-230ºC) IR(KBr): 3234, 3058, 2958, 2929, 2268, 1713, 1621, 1390, 1265,981, 893, 741 cm^-1^

2-(7-chloro-2-oxoindolin-3-ylidene)malononitrile (3n): M.P.: 245-246 ^O^C (Lit. [9]M.P. 245-246 ºC) . IR(KBr): 3193, 3072, 2988, 2929, 2859, 2223, 1723, 1620, 1587, 740 cm^-1^

2-hydroxy-1,2-diphenylethan-1-one (4a) White solide; M.P. :132-135 ℃(Lit. [10] M.P. 133-134 ºC); IR(KBr) ν (Cm^-1^): 3406, 3060, 2926, 1675, 1569, 1489, 1447, 1387, 1262, 1200, 1067, 973, 832, 748, 694, 599, 509 ; ms Molecular weight found :: 211

 2-hydroxy-1,2-di-p-tolylethan-1-one (4b) White powder; M.P. :65-67℃(Lit [10]. M.P. 84-85 ºC); ^1^H NMR (400 MHz, DMSO-*d*_6_) : δ 7.84 (d, J=12.0, 2H), 7.26 – 7.17 (m, 4H), 7.13-711 (d, J=8, 2H), 5.90 (s, 1H), 4.60 (bs, 1H), 2.35 (s, 3H), 2.29 (s, 3H). ^13^C NMR (100 MHz, DMSO- *d*_6_) :199.5 ,144.8 ,138.2, 136., 130.9, 129.9 ,129.7 ,1129.6 ,129.3 ,129.2 ,127.6 ,84.8, 29.6 ,21.6 ,21.1 .IR (KBr) ν (Cm-1): 3457, 3028, 2922, 1676, 1607, 1511, 1396, 1247, 1180, 1078, 973, 814, 723, 659, 576, 503 ; Ms Molecular weight found : 240

1,2-bis(4-bromophenyl)-2-hydroxyethan-1-one (4c) Yellow solid; M.P. :94-96 ℃ Lit[10]. M.P. 94-96 ºC) ; IR (KBr) ν (Cm^-1^): 3438, 2919, 2852, 1669, 1580, 1481, 1393, 1267, 1175, 1070, 1008, 817, 753, 607, 523, 461 ;ms Molecular weight found :: 367.9

1,2-bis(3-bromophenyl)-2-hydroxyethan-1-one (4d) Solid; M.P. : 76-77 ℃ Lit. [11] M.P. 75-77 ºC); IR (KBr) ν (Cm^-1^): 3427, 3065, 2922, 2854, 1686, 1576, 1470, 1418, 1260, 1193, 1073, 990, 886, 782, 740, 690 ; ms Molecular weight found :: 368

2-hydroxy-1,2-bis(4-methoxyphenyl)ethan-1-one (4e) Solid ; M.P. : 109-11℃ Lit. [10]. M.P. 110-112 ; IR (KBr) ν (Cm^-1^): 3420, 2924, 2853, 1680, 1608, 1511, 1441, 1277, 1172, 1106, 1022, 846, 761, 695, 605, 513: Molecular weight found :: 272

1,2-di(furan-2-yl)-2-hydroxyethan-1-one (4f) White solid; M.P. :134-135℃ ; Lit. [12] M.P. 134-135 IR (KBr) ν (Cm^-1^): 3416, 3147, 3124, 3093, 1674, 1592, 1523, 1444, 1383, 1307, 1209, 1141, 1016, 926, 883, 755, 596 ; Molecular weight found :: 192

2-hydroxy-1,2-di(thiophen-2-yl)ethan-1-one (4g )Yellow Solid ; M.P. : 107-108℃; Lit. [13] M.P. 108-109 ; IR (KBr) ν (Cm^-1^): 3440, 3068, 2945, 1641, 1453, 1392, 1274, 1091, 1022, 939, 882, 789, 584 ; Molecular weight found :: 224

 2-hydroxy-1,2-bis(4-nitrophenyl)ethan-1-one (4h) Solid; M.P. : 239 ℃ ; Lit. [14]M.P. 239-240 IR (KBr) ν (Cm^-1^): 3410, 3062, 2924, 1682, 1632, 1595, 1511, 1435, 1279, 1170, 1109, 844, 768, 693, 603, 507 ; Molecular weight found : 302

1,2-bis(4-chlorophenyl)-2-hydroxyethan-1-one (4i)M.P.:87-89 [10] IR (KBr) ν (Cm^-1^): 3463,2986,1893,1743,1374,1243,1048,937,847,608; Molecular weight found : 280

 Choline Formate red- liquid (5a) : ^1^H NMR (400 MHz, MeOD) δ 8.57 (s, 1H), 4,96 (s,) 4.04 (dq, *J* = 7.9, 2.9 Hz, 2H), 3.68 – 3.46 (m, 2H), 3.27 (d, *J* = 1.2 Hz, 9H). ^13^C NMR (100 MHz, MeOD) δ 168.94, 159.82, 67.71, 55.76, 54.14 – 53.16 (m). IR( KBr ): 3457 ,2960 ,2851 ,2164 ,1648 ,1484 ,1484 ,1385 ,1135 cm^-1^ Molecular weight found :149

 Choline cyanide (5b) orange- liquid: ^1^H NMR (400 MHz, MeOD) : δ 4,91 (s,HOD) 4.08 – 4.04 (m, 2H), 3.66 – 3.61 (d, *J*=8 Hz 2H), 3.33 (S, 9H). ^13^C NMR (100 MHz, MeOD) δ 163.27 (C-N) [15], 67.83 – 67.62 (m), 55.47, 53.99 – 53.76 (m). IR( KBr ): 3375 ,3186,3060,2914,2201,1915,1719,1655,1600,1482, 1350,1204,1083,812,619,570 cm^-1^  Molecular weight found :131


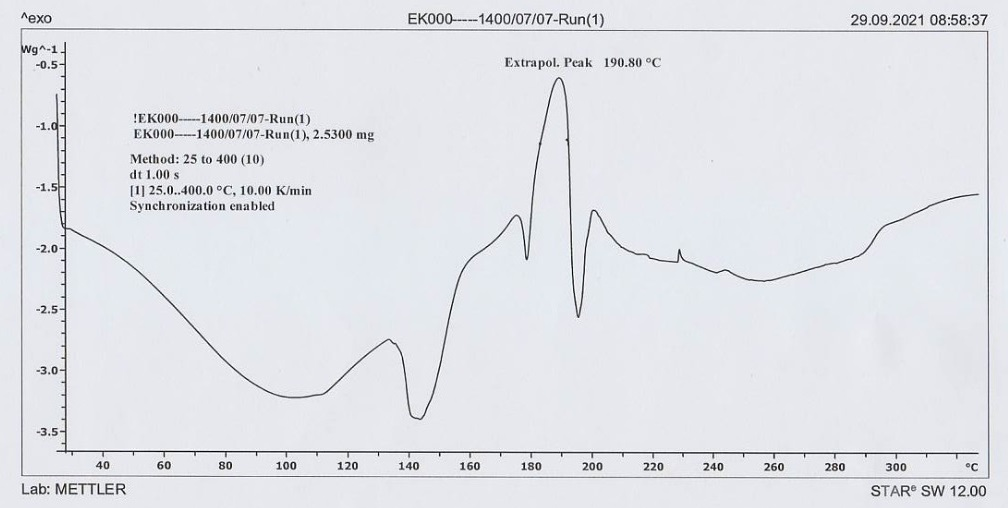


**FigureS_1_** DSC of choline cyanide


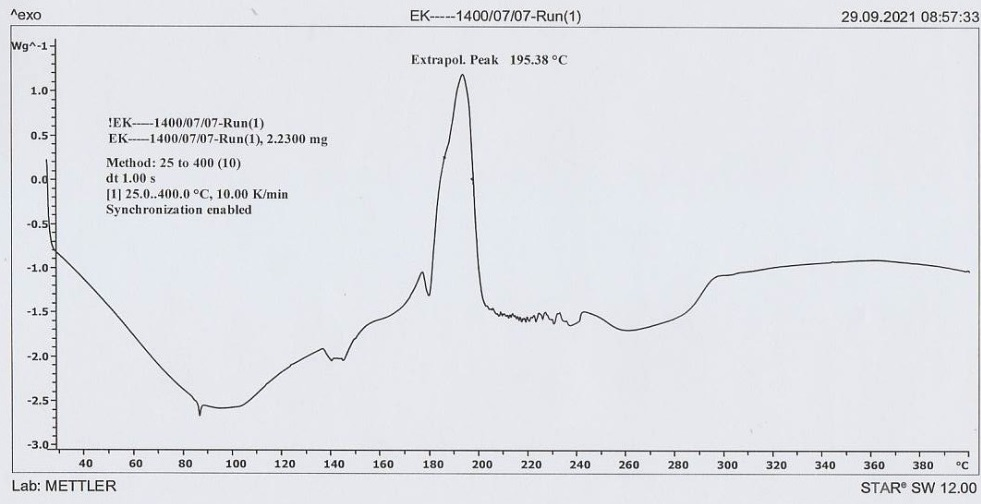


**FigureS_2_** DSC of choline formate


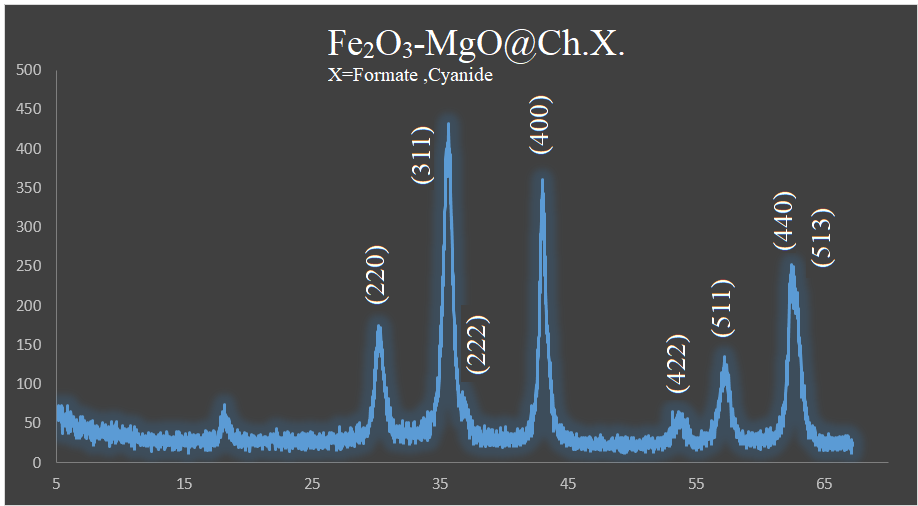


**Figure. S_3_.** XRD of reuse Fe_2_O_3_-MgO@Ch.F and Fe_2_O_3_/MgO@Ch.CN


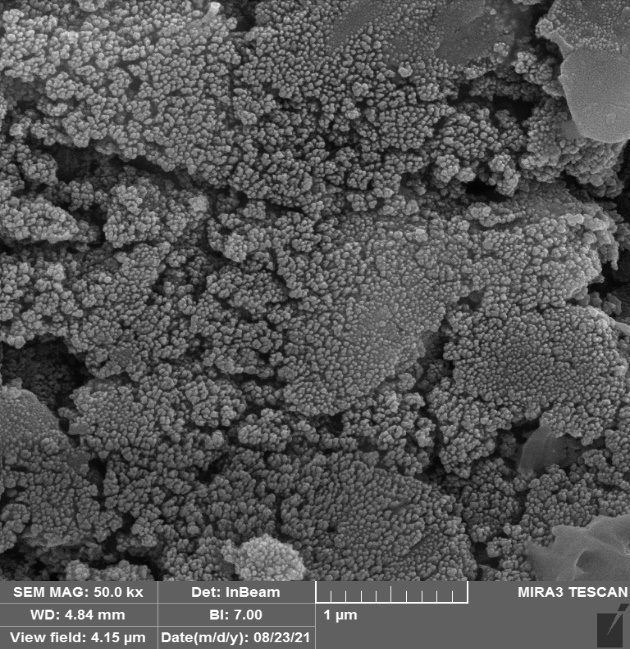


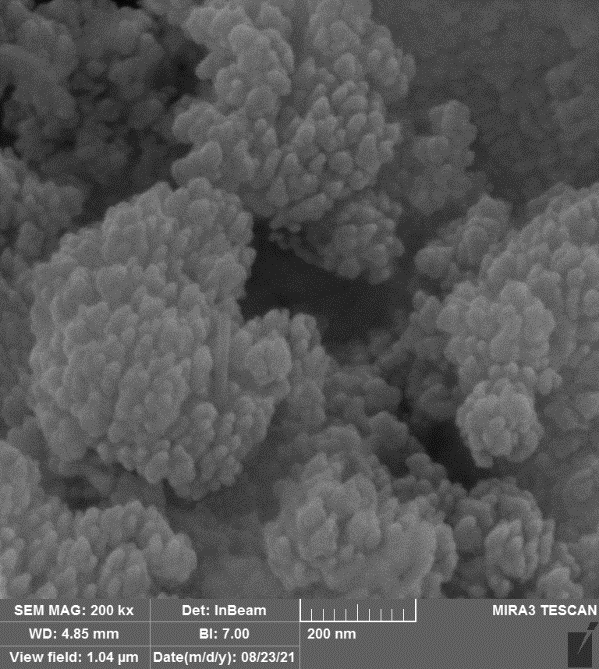


(B)

(A)

**Figure. S_4_** SEM images of reuse γ-Fe_2_O_3_/MgO@Ch.F (A) and γ-Fe_2_O_3_/MgO@Ch.CN (B)

**Figure S_5_**. VSM of reuse γ-Fe_2_O_3_/MgO@Ch.F. and γ-Fe_2_O_3_/MgO@Ch.CN


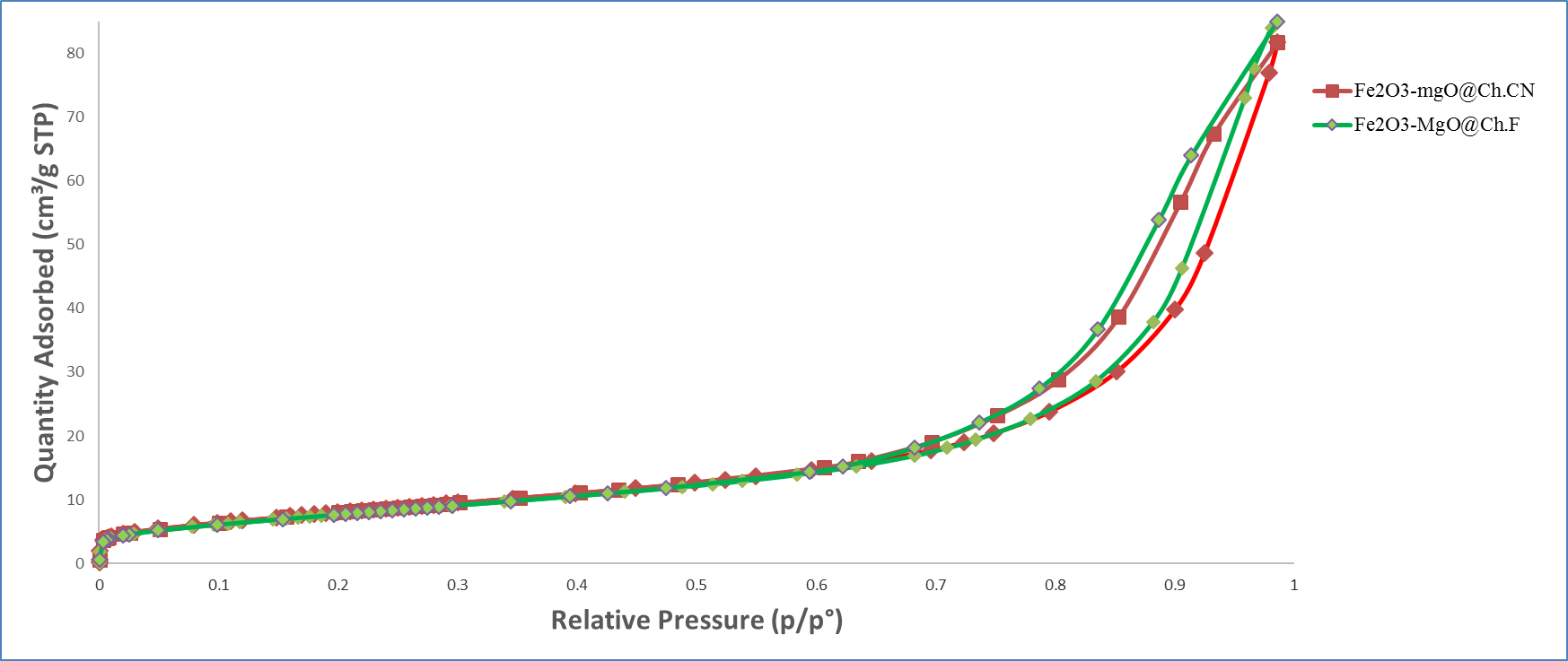


**Figure. S_6_.** BET results of Fe_2_O_3_-MgO@Ch.CN and Fe_2_O_3_/MgO@Ch.F.


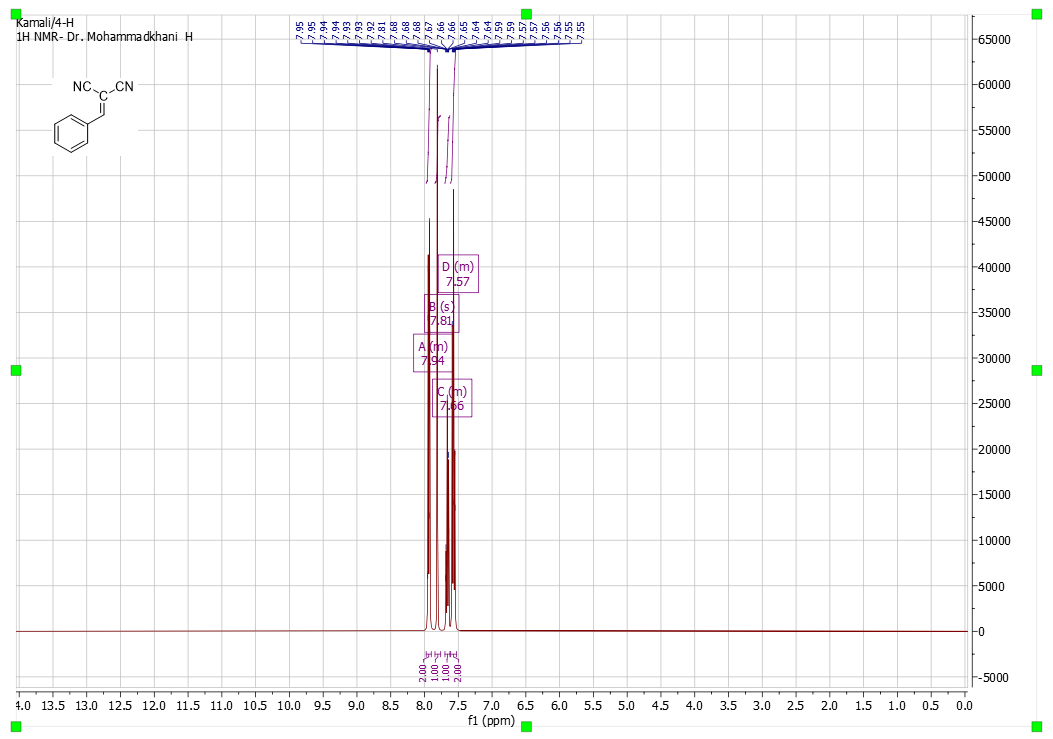


**FigureS_7_** the ^1^H NMR spectrum of 3a in CDCl_3_


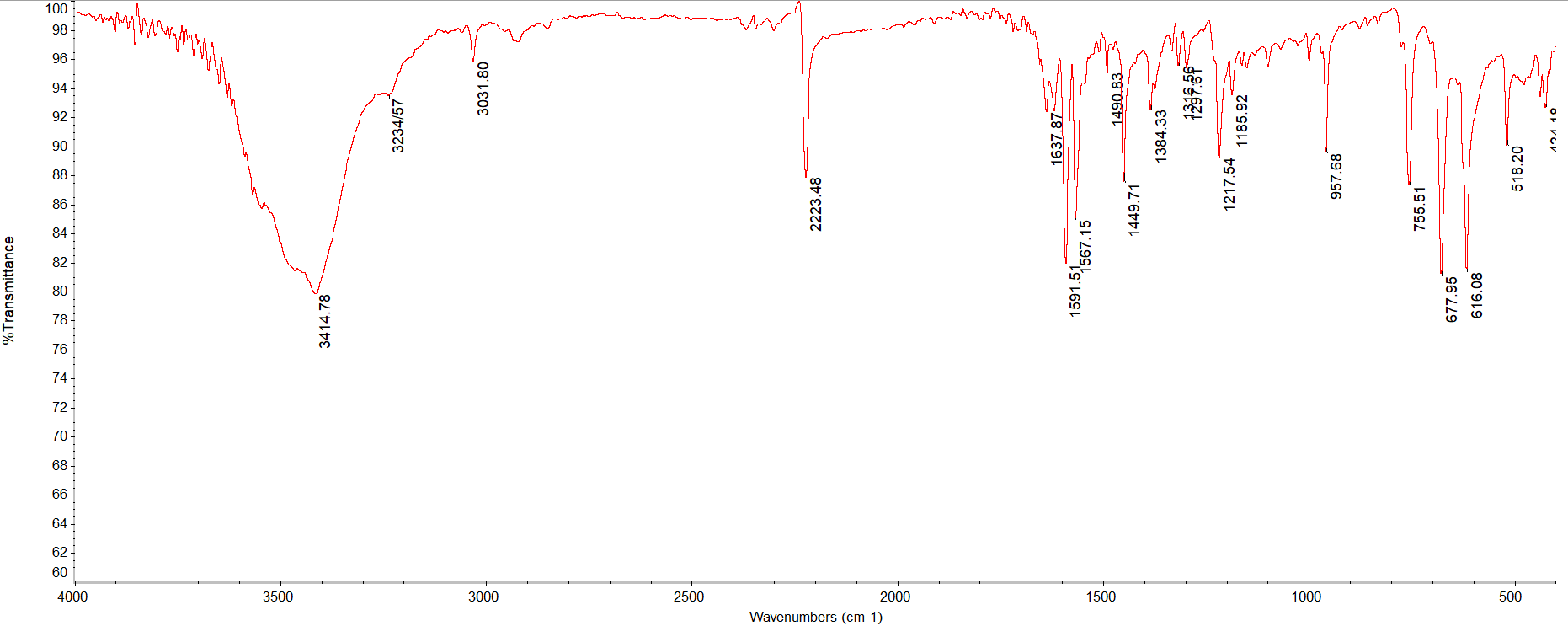


**FigureS_8_**the FT-IR spectrum of 3a


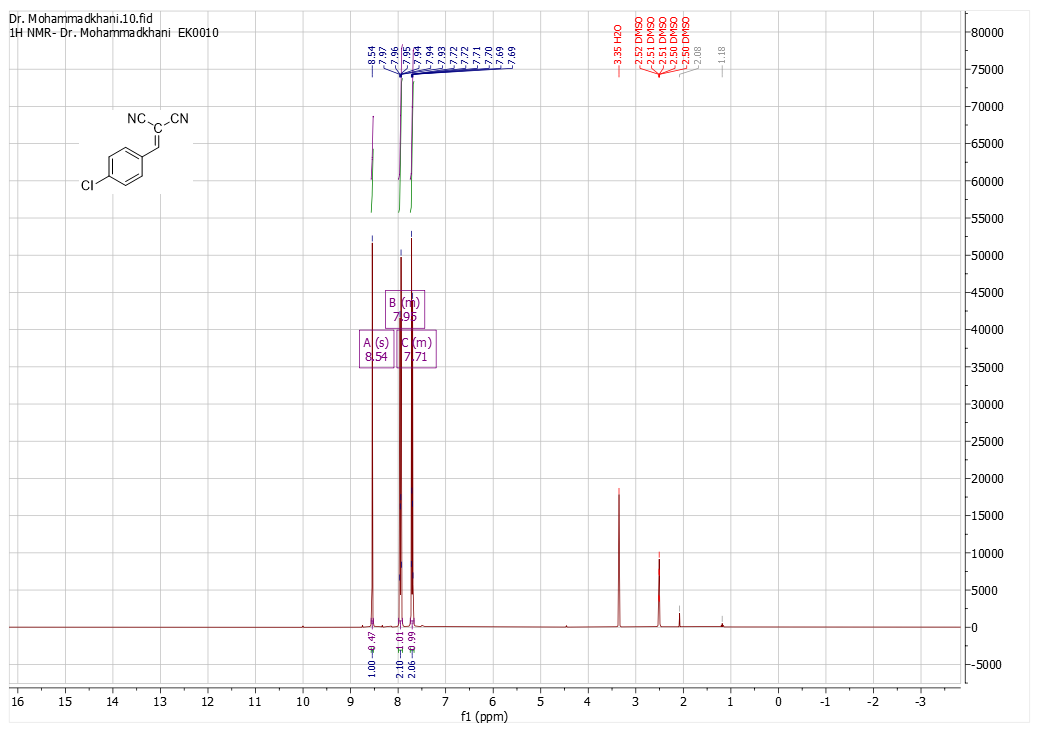


**FigureS_8_** the ^1^H NMR spectrum of 3b in CDCl_3_


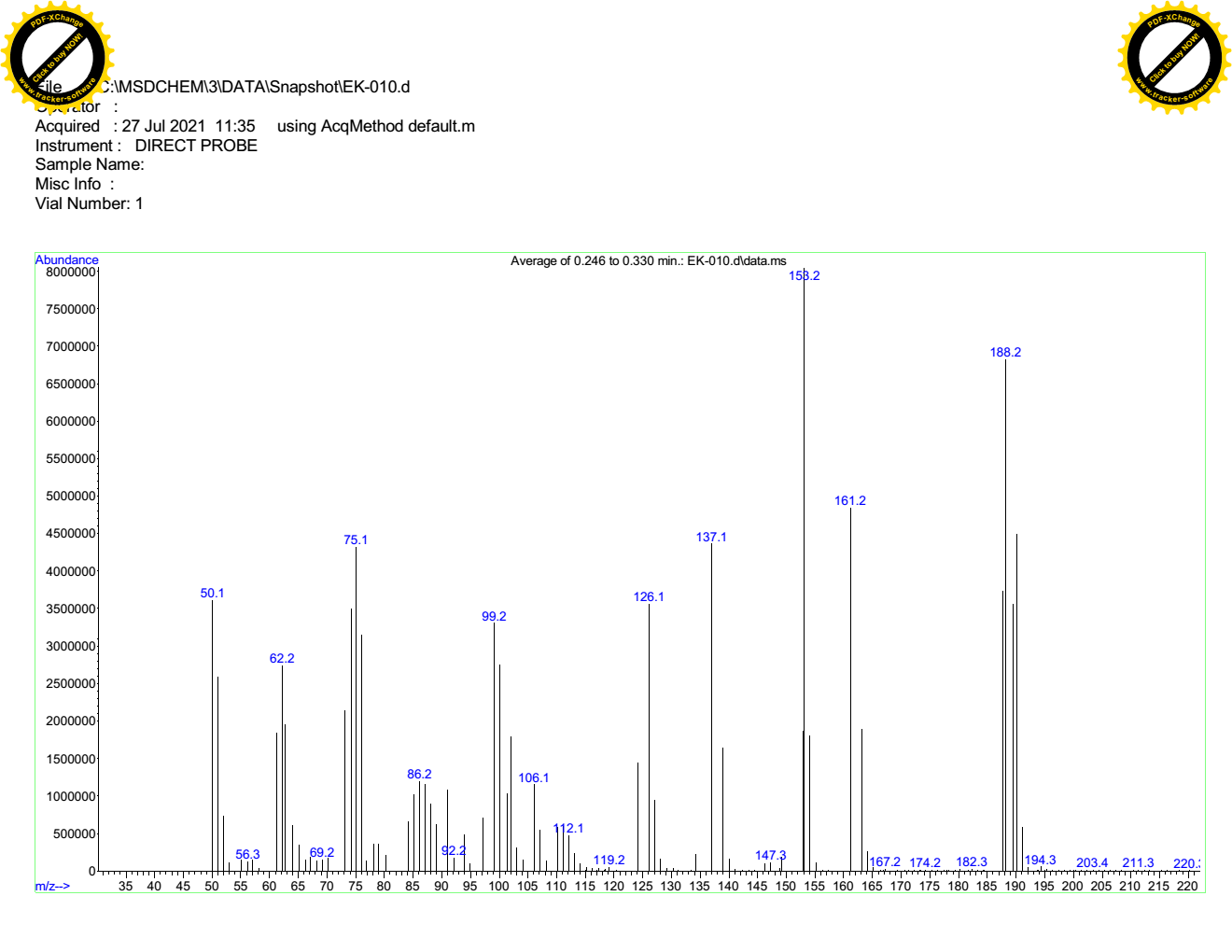


**FigureS_10_** Mass spectrum of 3b


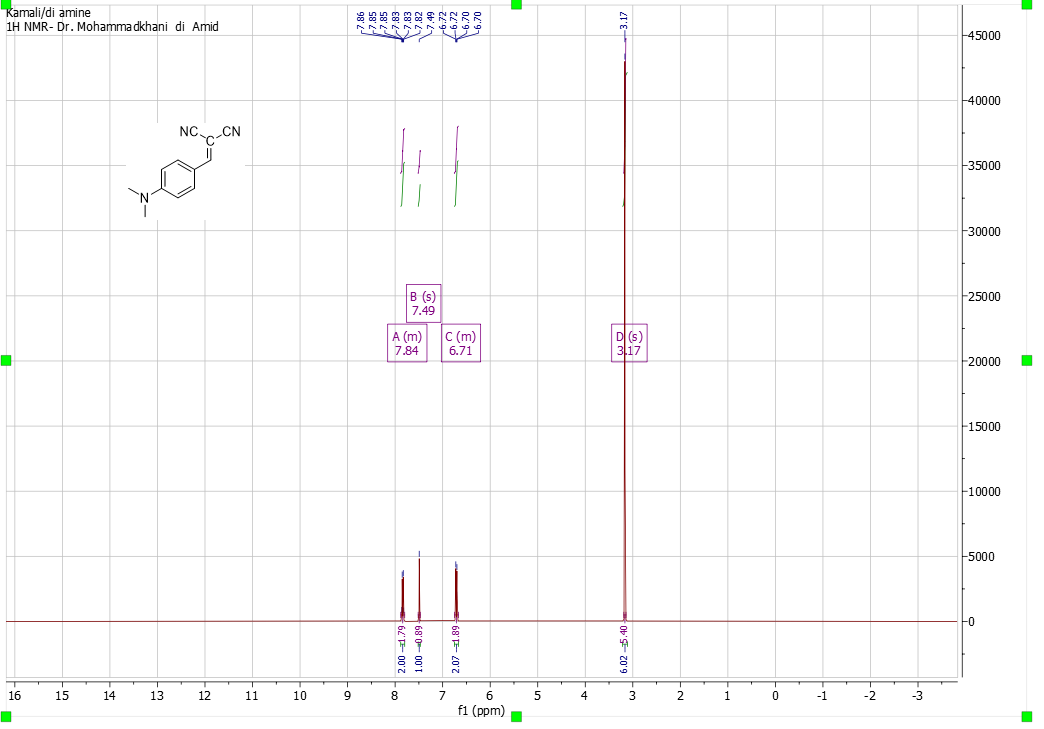


_._ **FigureS_11_**the ^1^H NMR spectrum of 3c in CDCl_3_


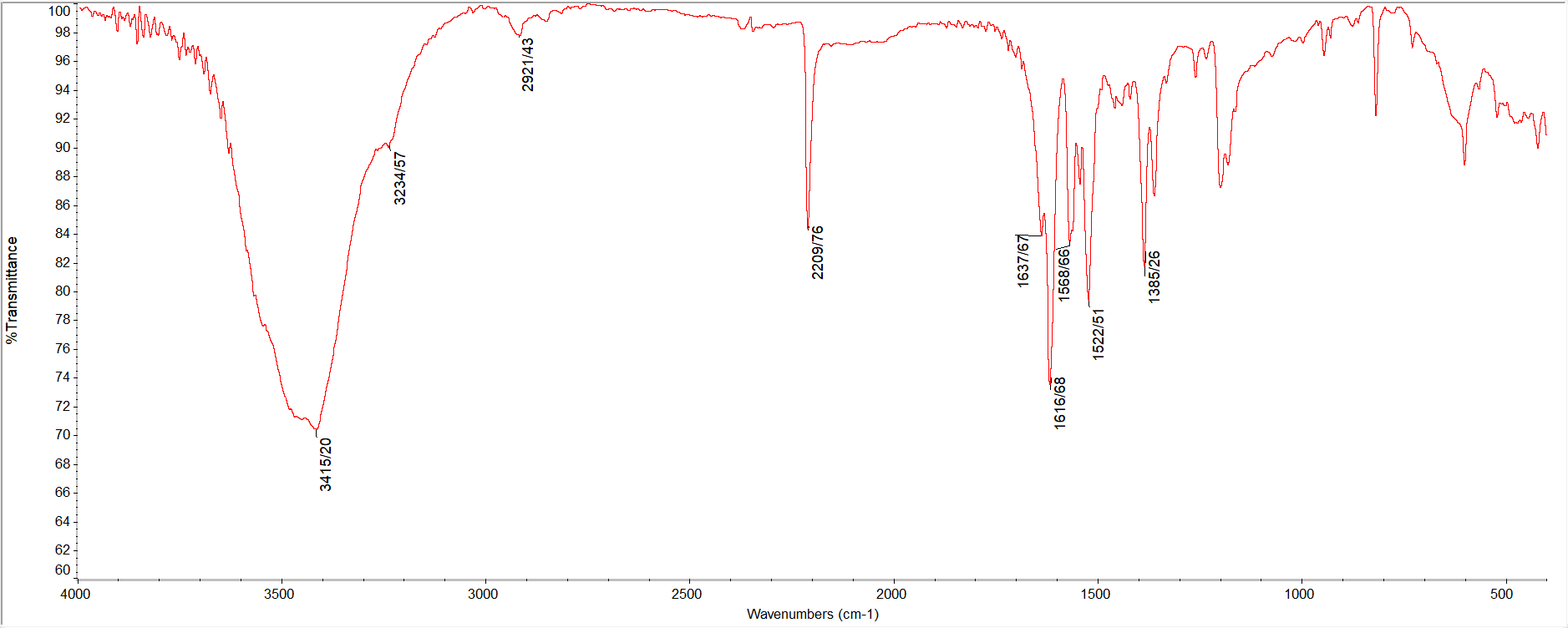


_._ **FigureS_12_** the FT-IR spectrum of 3c

_
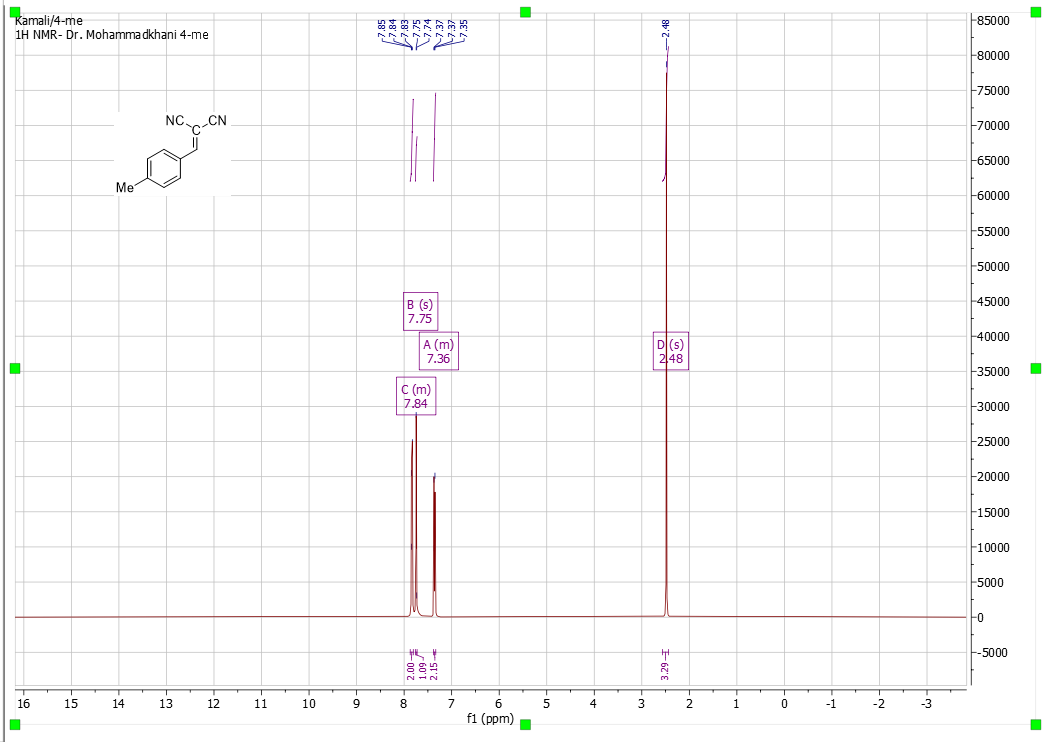
_

_._ **FigureS_13_**the ^1^H NMR spectrum of 3d in CDCl_3_


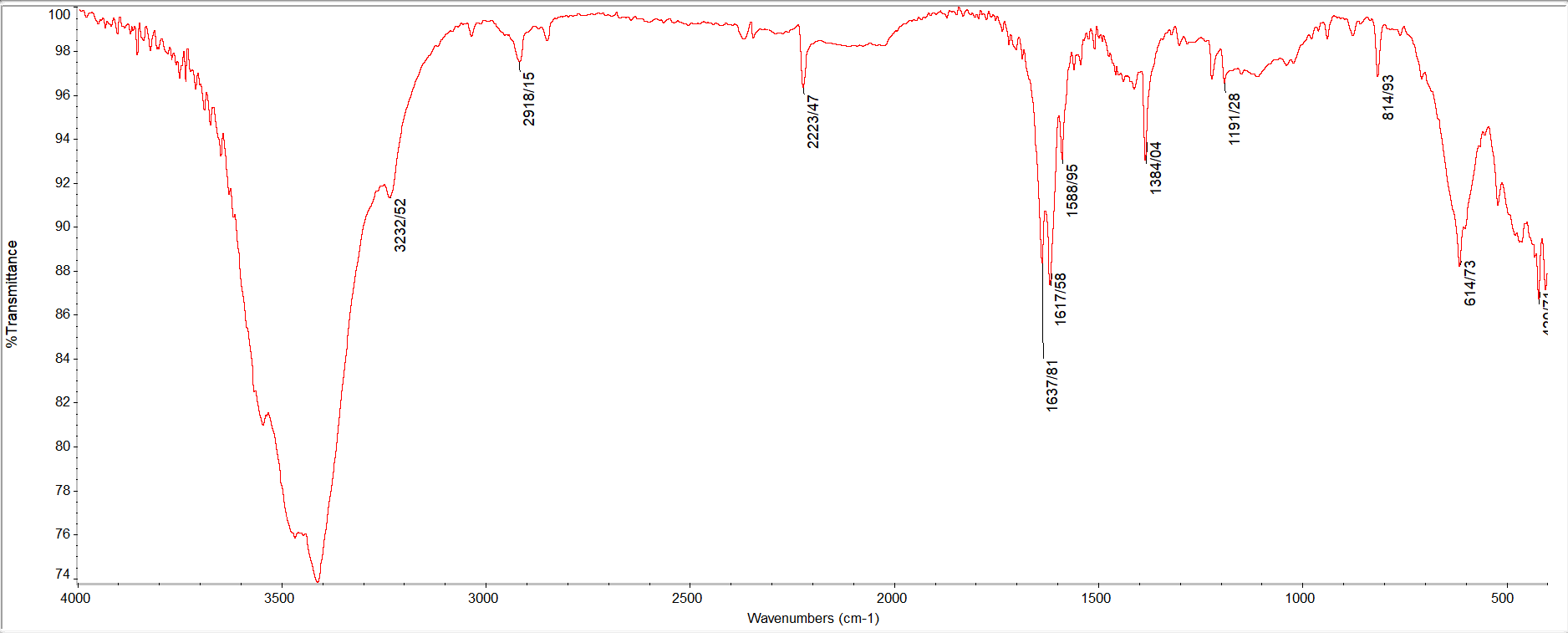


_._ **FigureS_14_** the ^1^H NMR spectrum of 3d in CDCl_3_


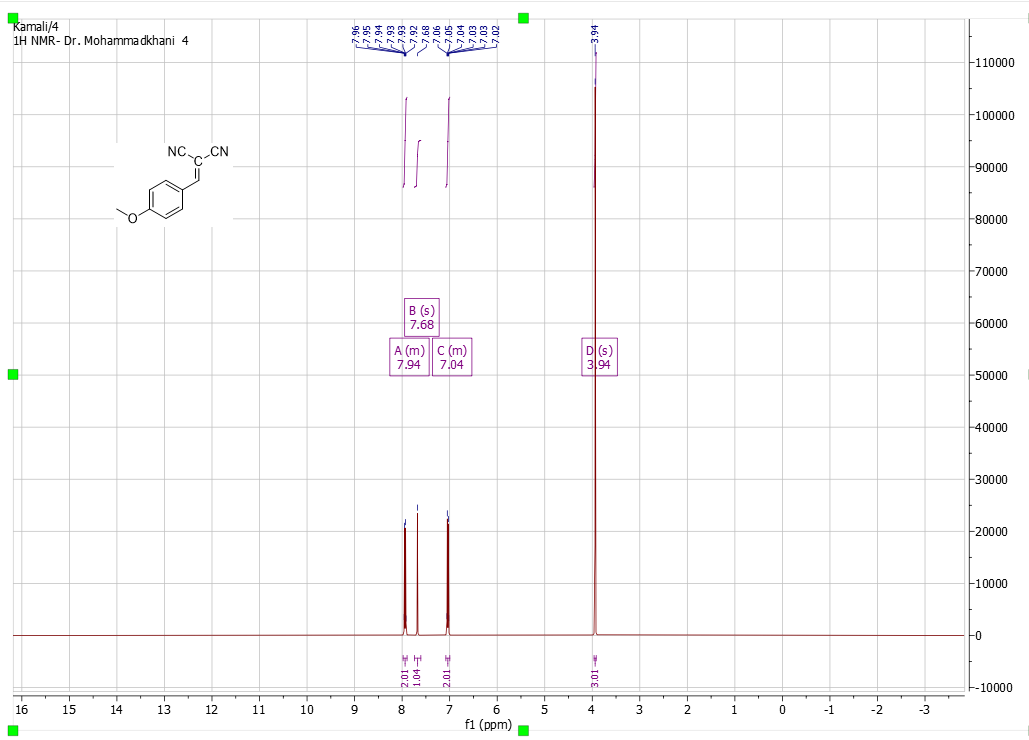


_._ **FigureS_15_**the ^1^H NMR spectrum of 3e in CDCl_3_


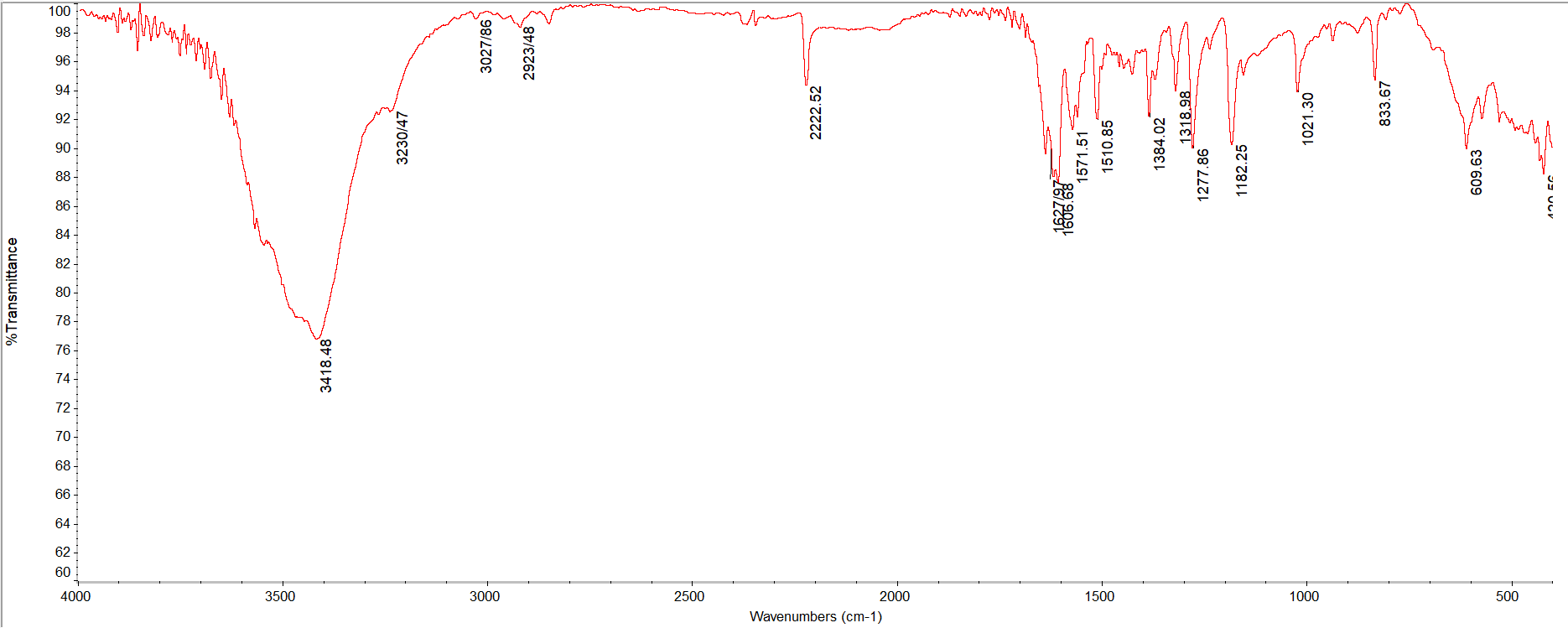


**FigureS_16_** the FT-IR spectrum of 3e

_
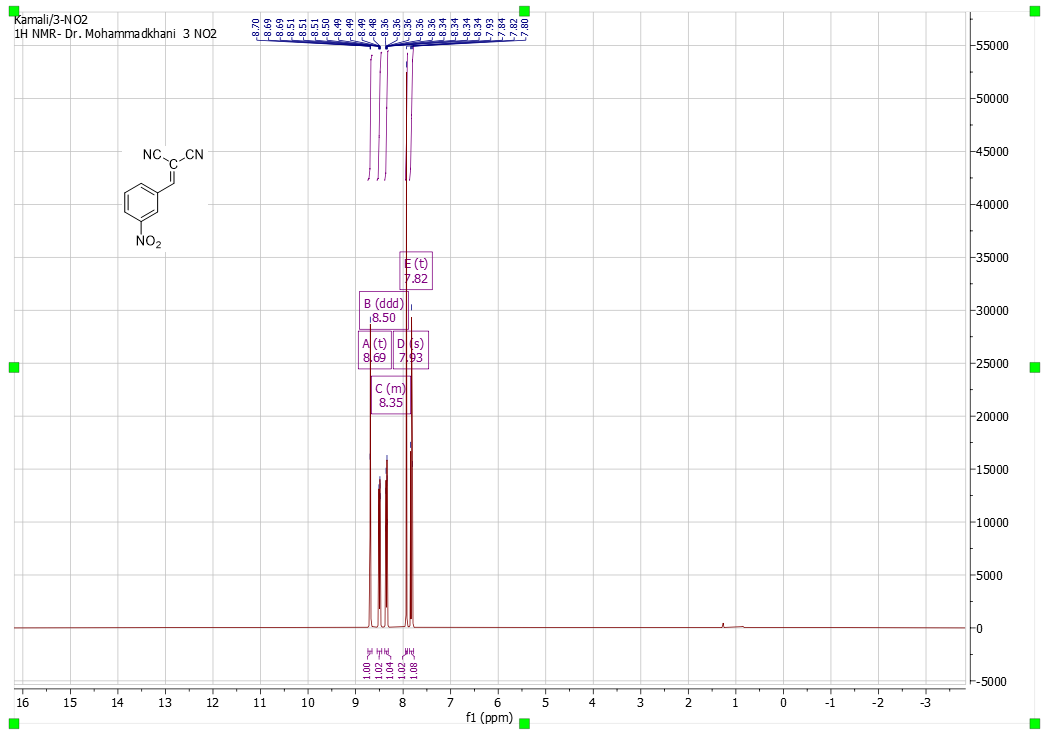
_

_._ **FigureS_17_**the ^1^H NMR spectrum of 3f in CDCl_3_


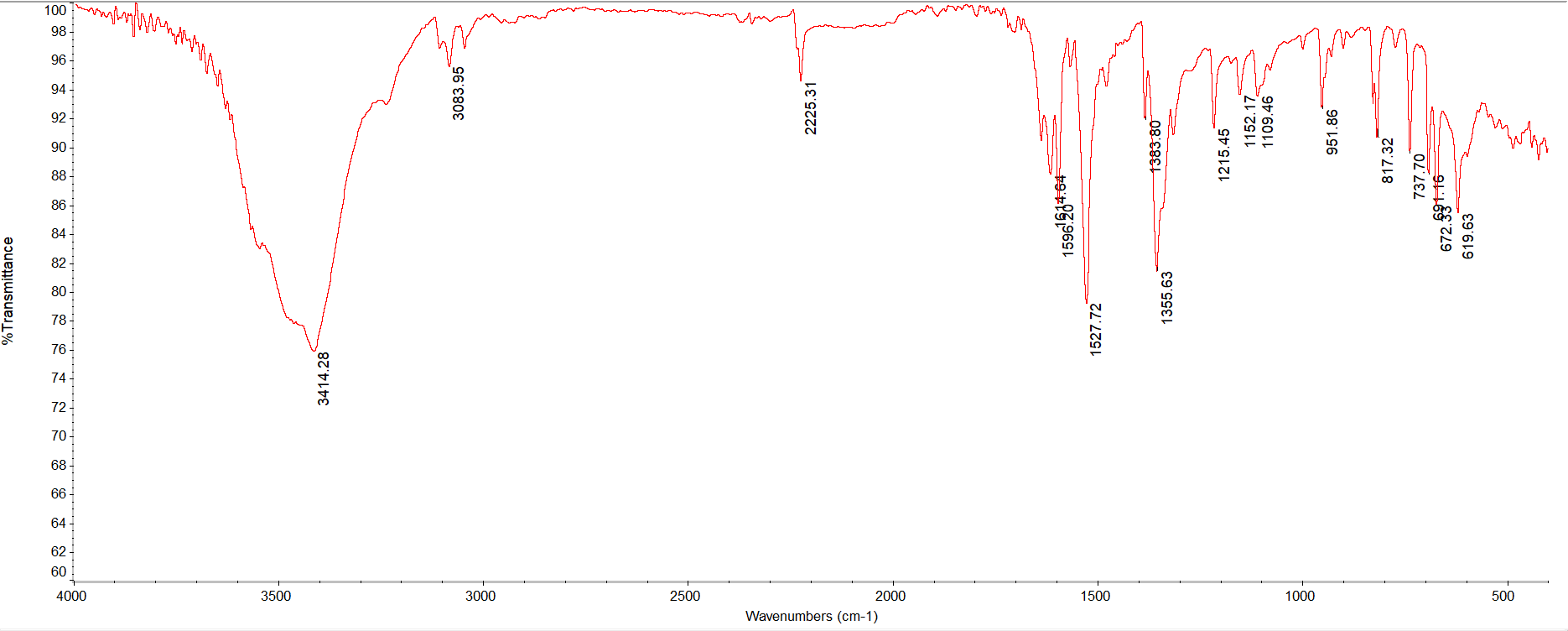


**FigureS_18_**the FT-IR spectrum of 3f

_
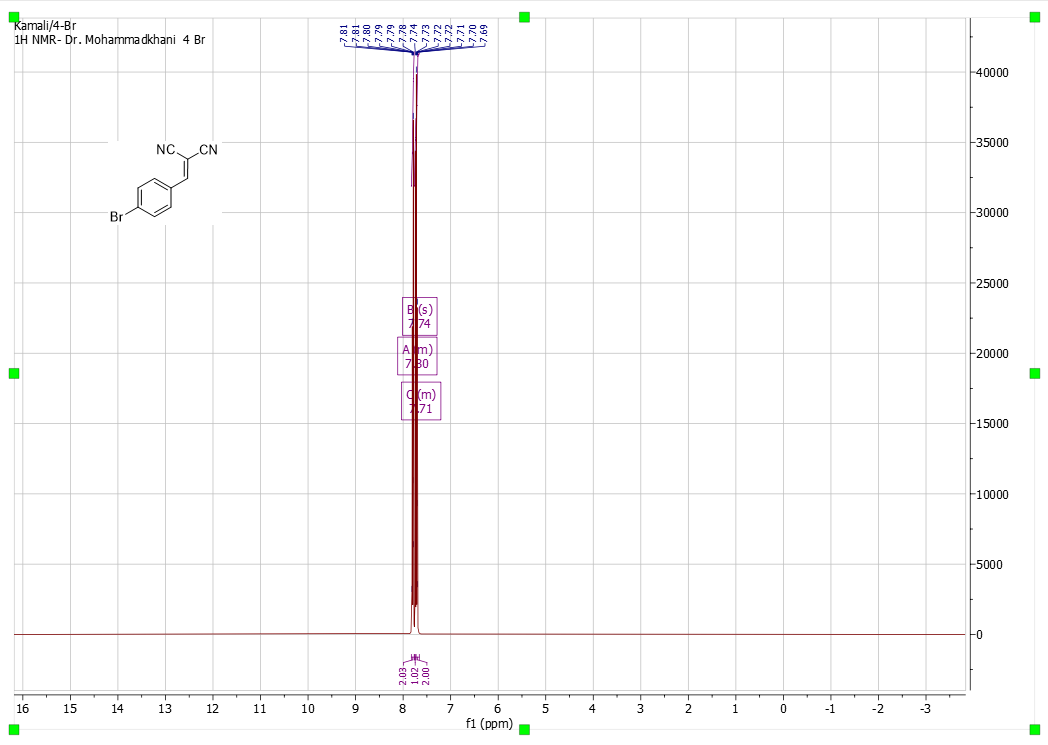
_

_._ **FigureS_19_**the ^1^H NMR spectrum of 3g in CDCl_3_


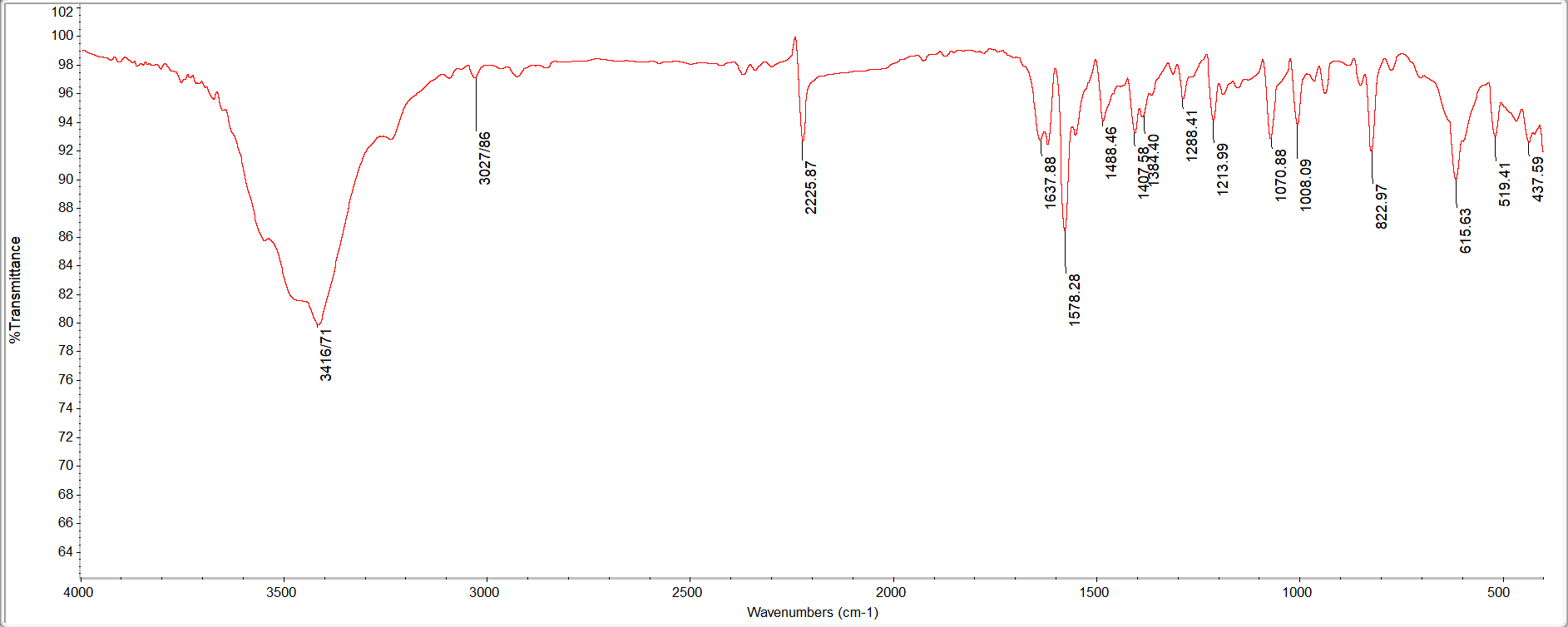


**FigureS_20_** the FT-IR spectrum of 3g

_
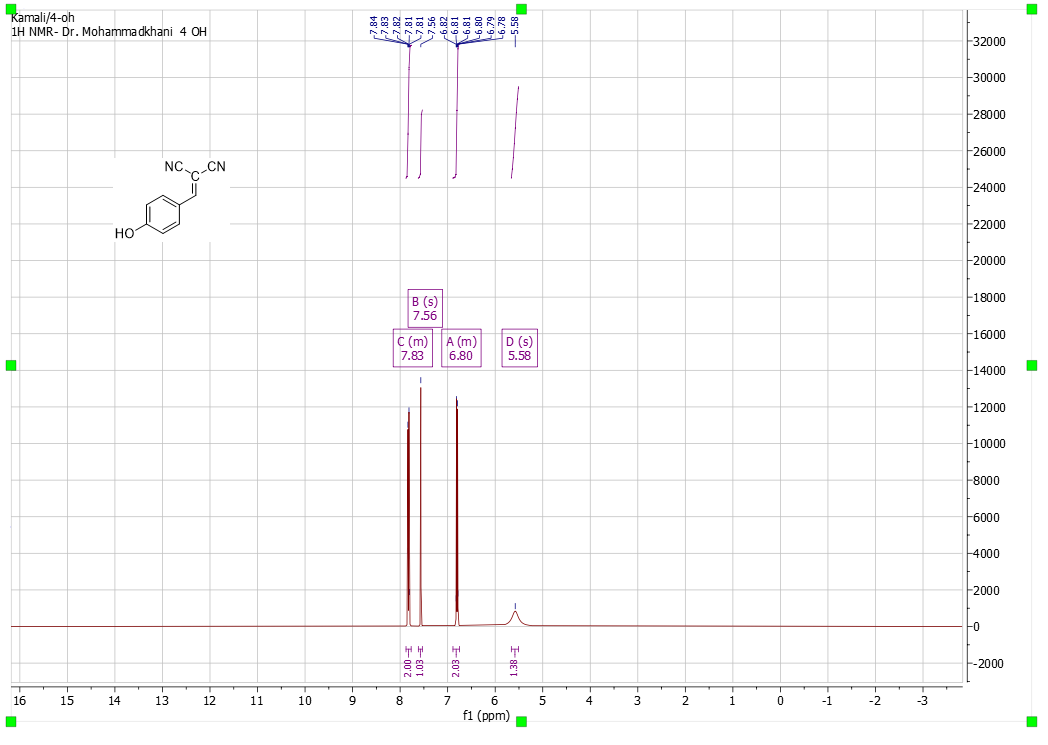
_

**FigureS_21_**the ^1^H NMR spectrum of 3h in CDCl_3_


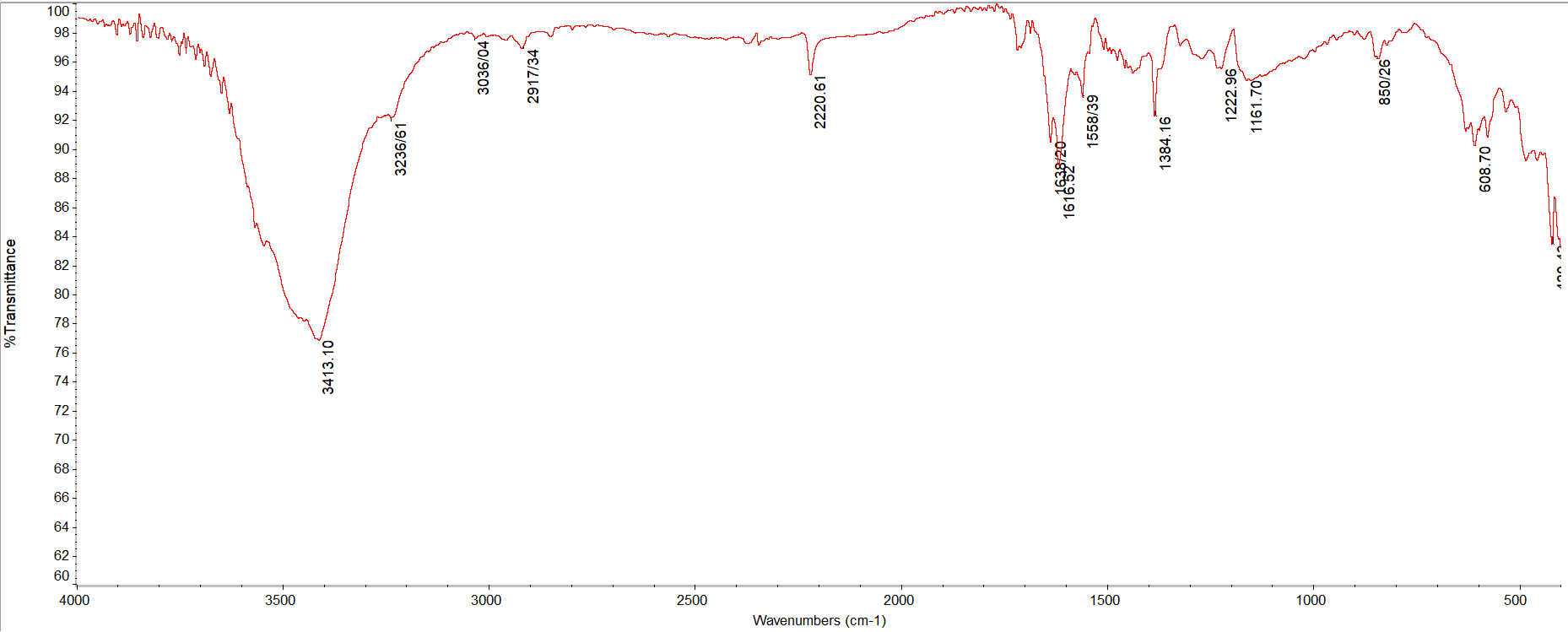


**FigureS_22_**the FT-IR spectrum of 3h

_
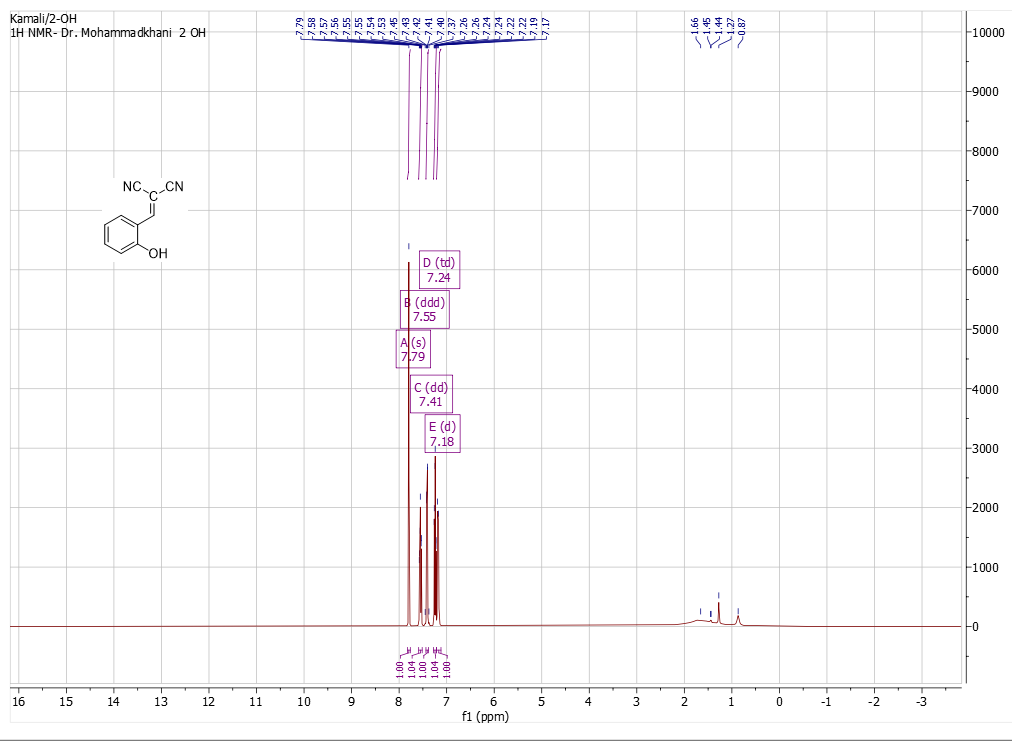
_

**FigureS_23_** the ^1^H NMR spectrum of 3i in CDCl_3_

_
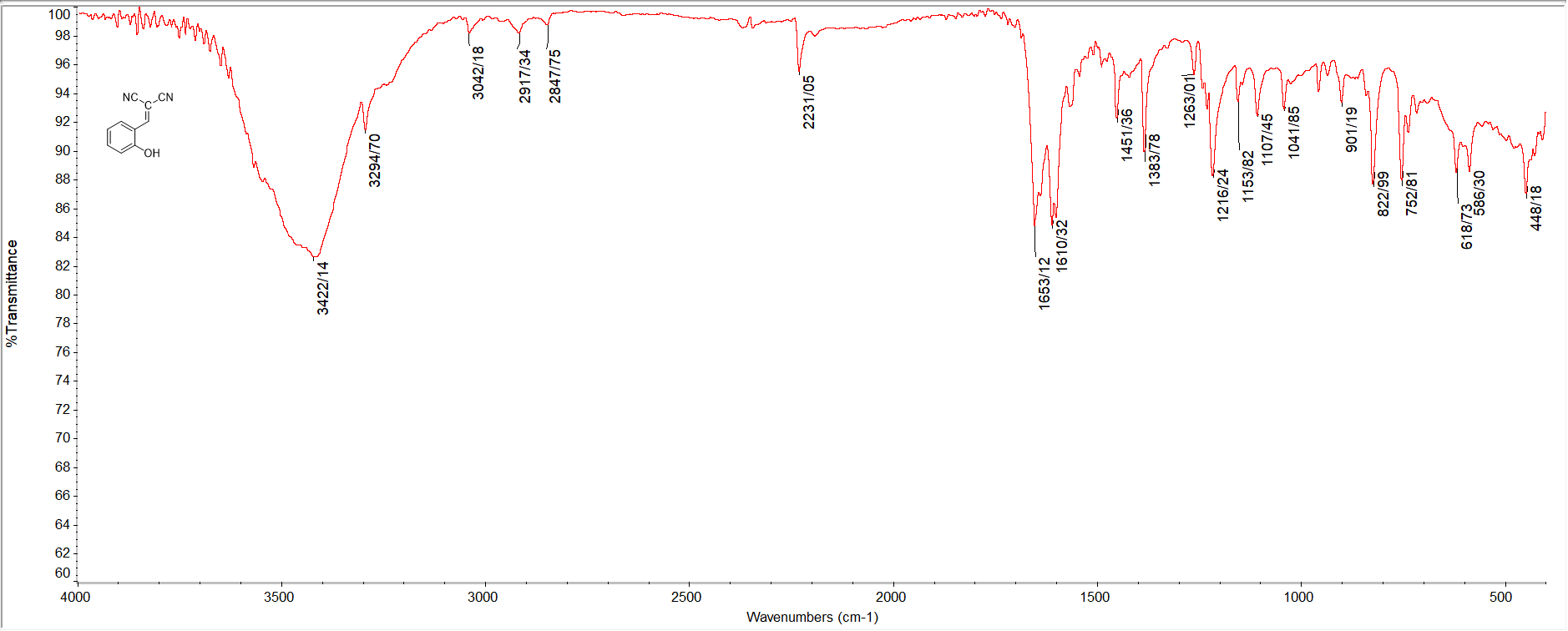
_

**FigureS_24_** the FT-IR spectrum of 3i

_
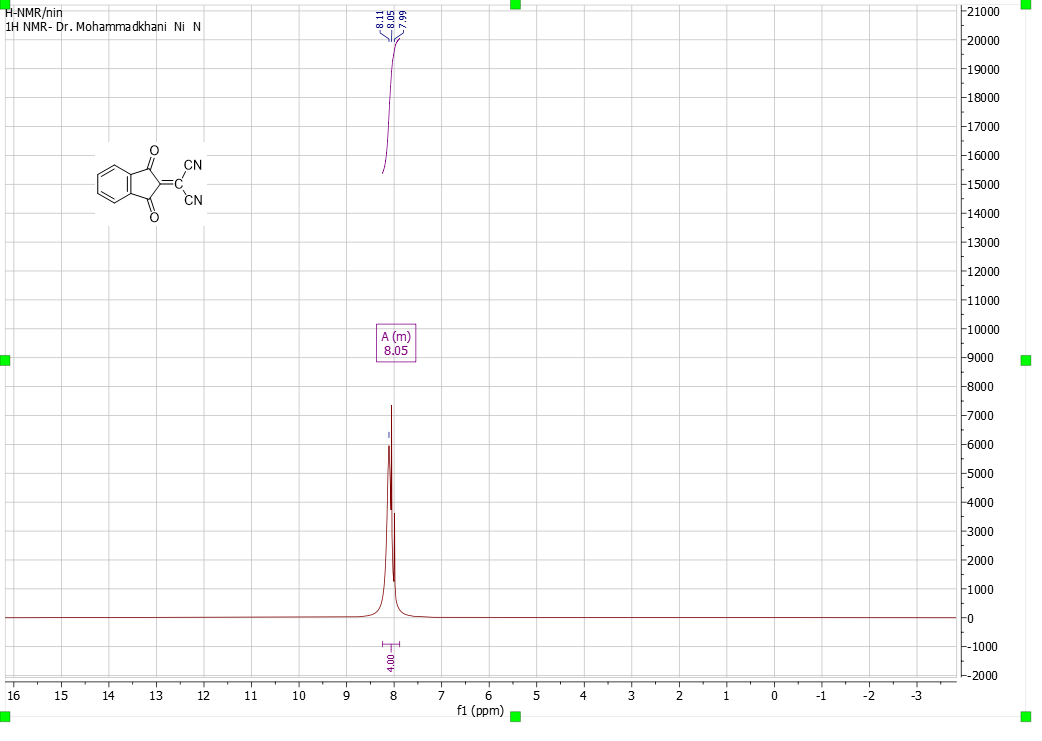
_

**FigureS_25_** the ^1^H NMR spectrum of 3j in CDCl_3_


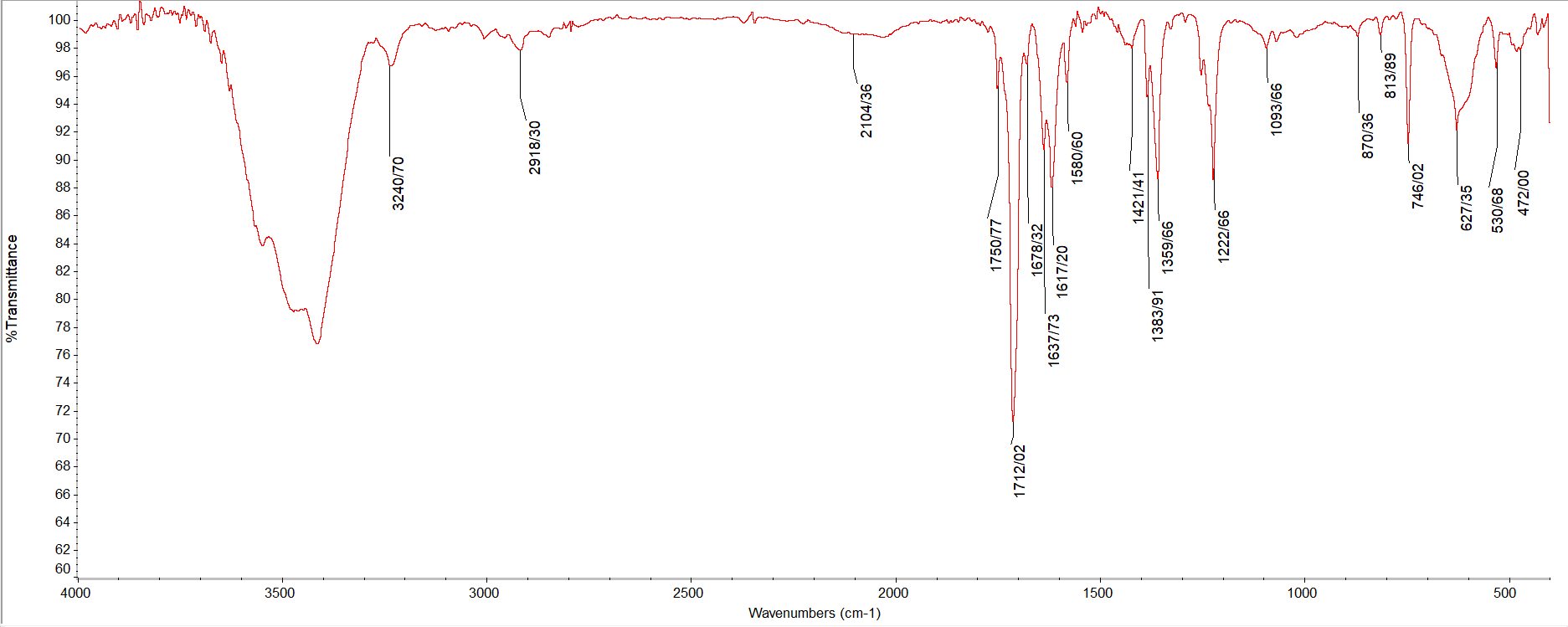


**FigureS_26_**the FT-IR spectrum of 3j

_
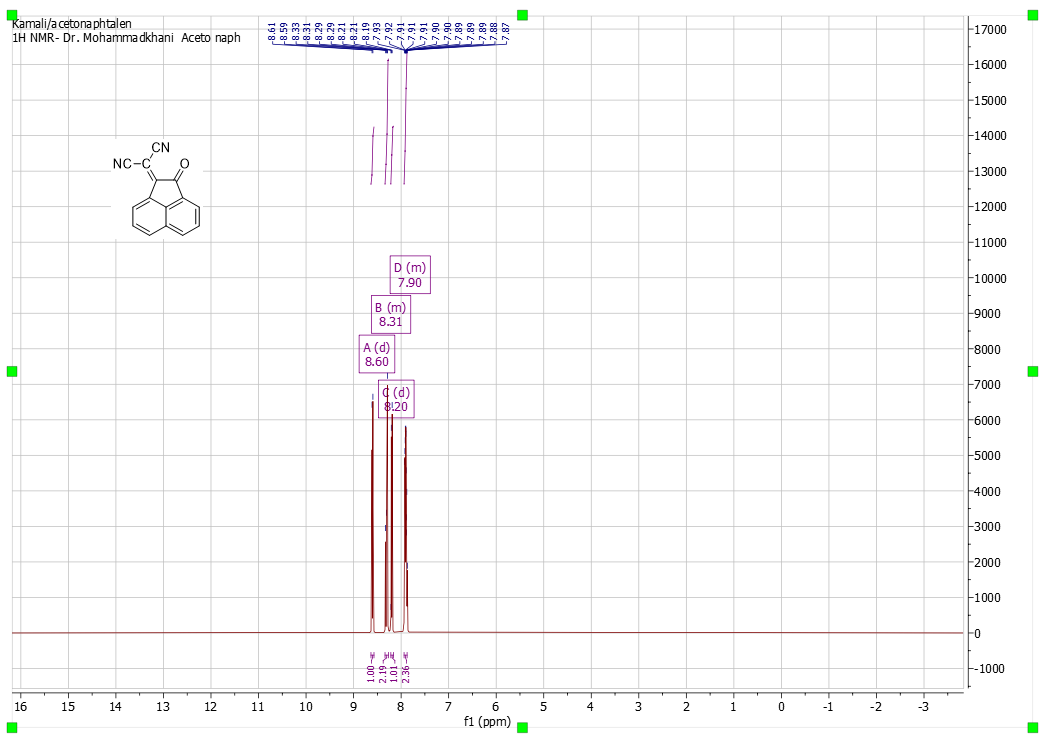
_

**FigureS_27_** the 1H NMR spectrum of 3k in CDCl_3_


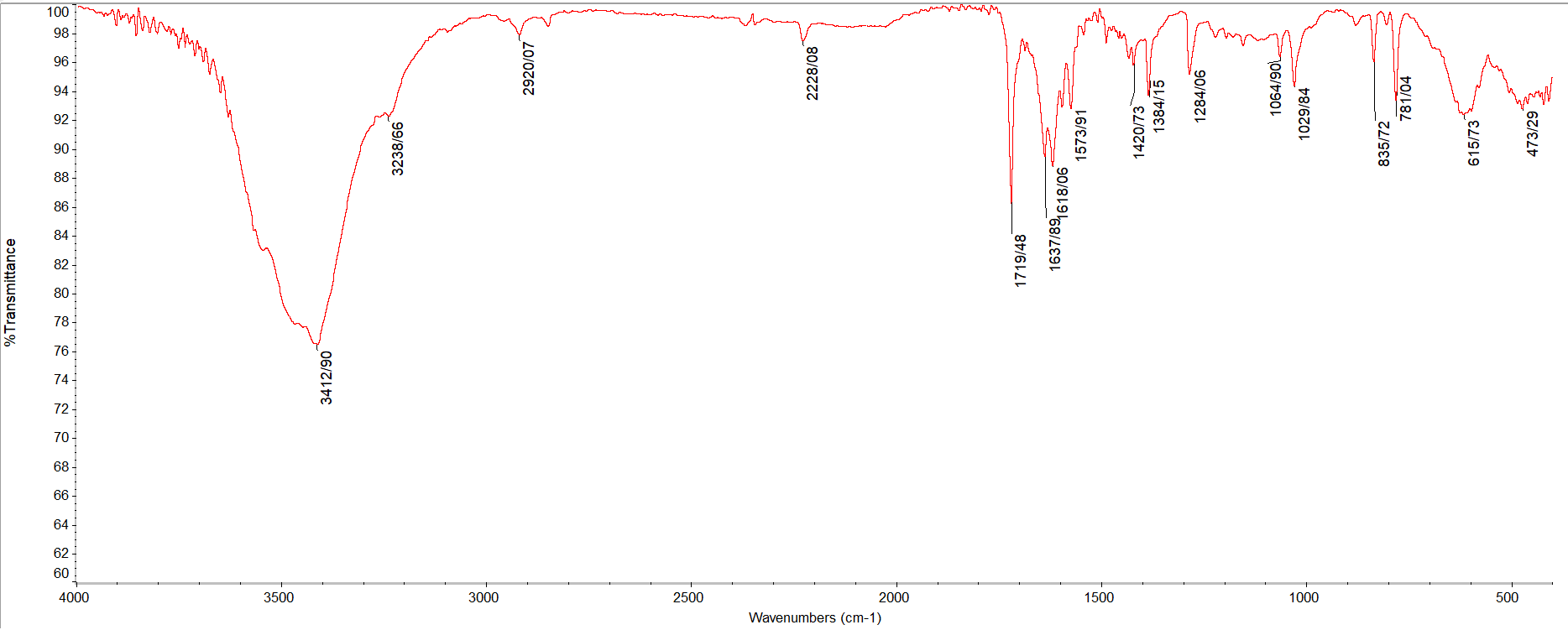


**FigureS_28_**the FT-IR spectrum of 3k


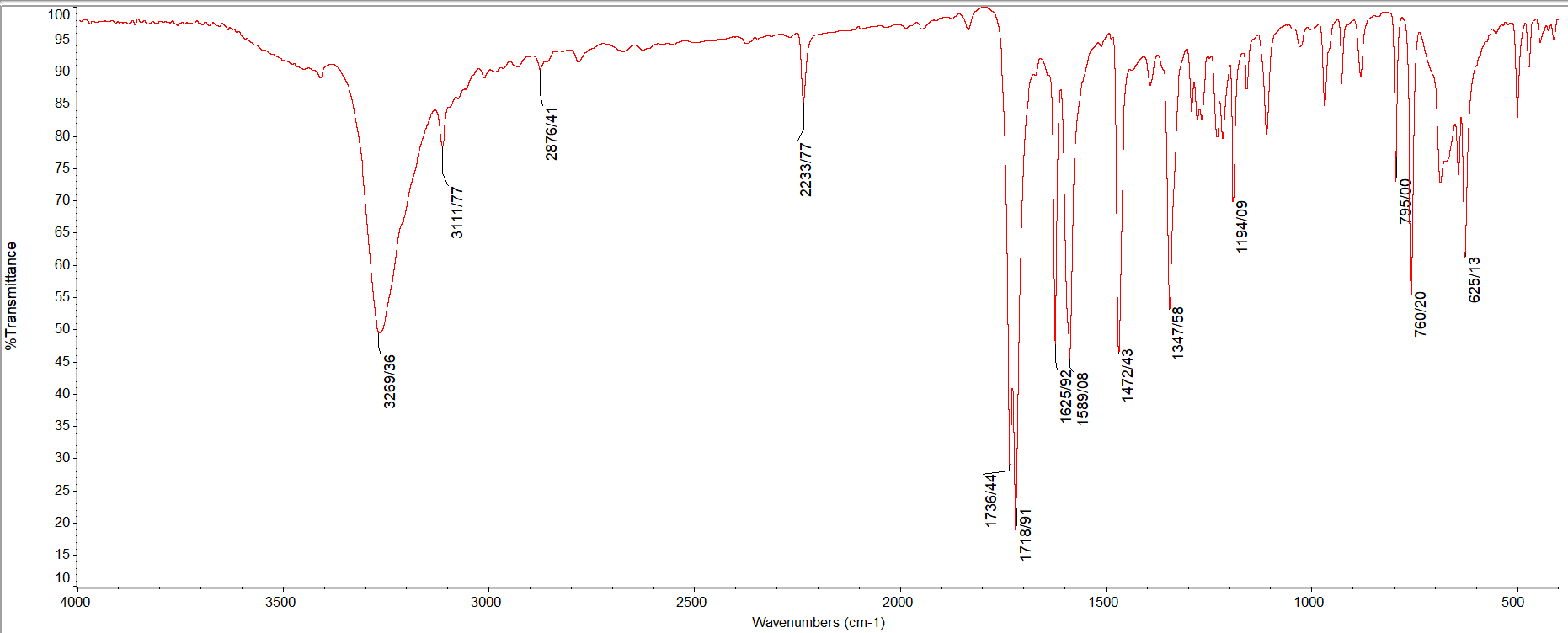


**FigureS_29_** the FT-IR spectrum of 3l in CDCl_3_


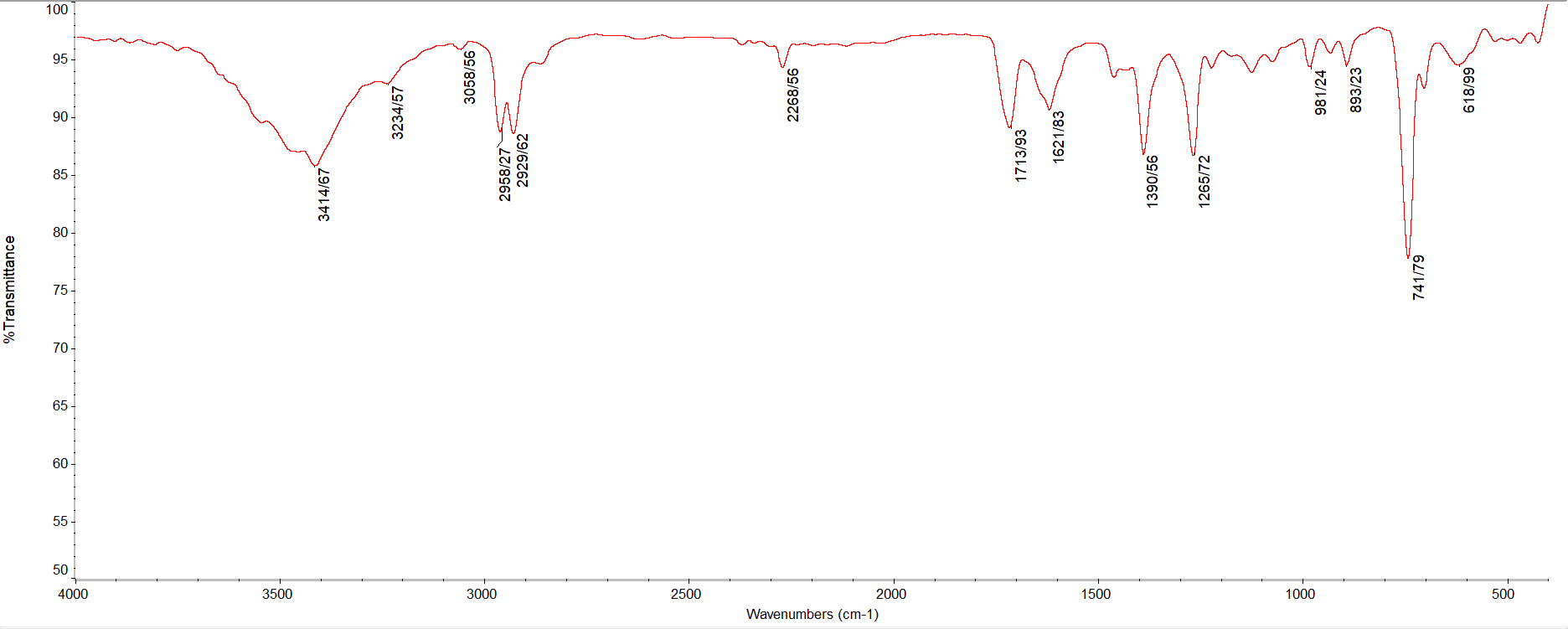


**FigureS_30_** the FT-IR spectrum of 3m


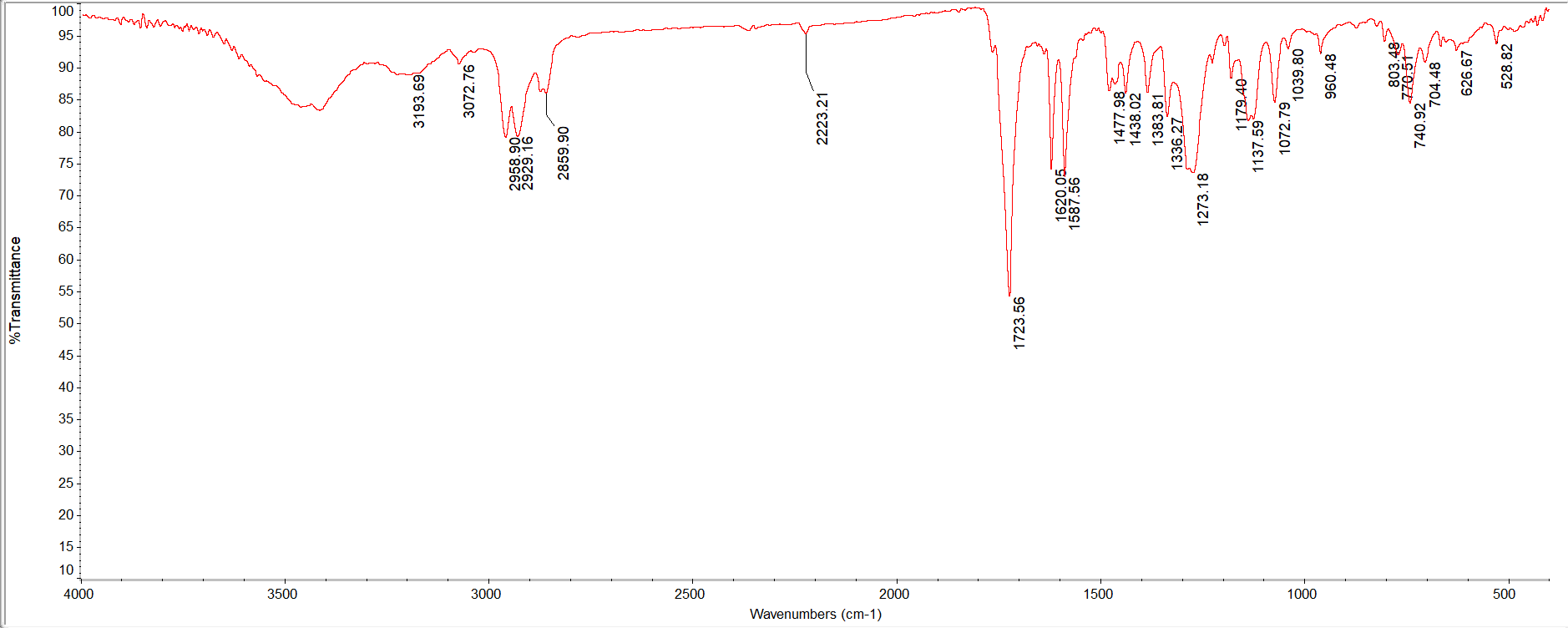


**FigureS_31_** the the FT-IR spectrum 3n

211


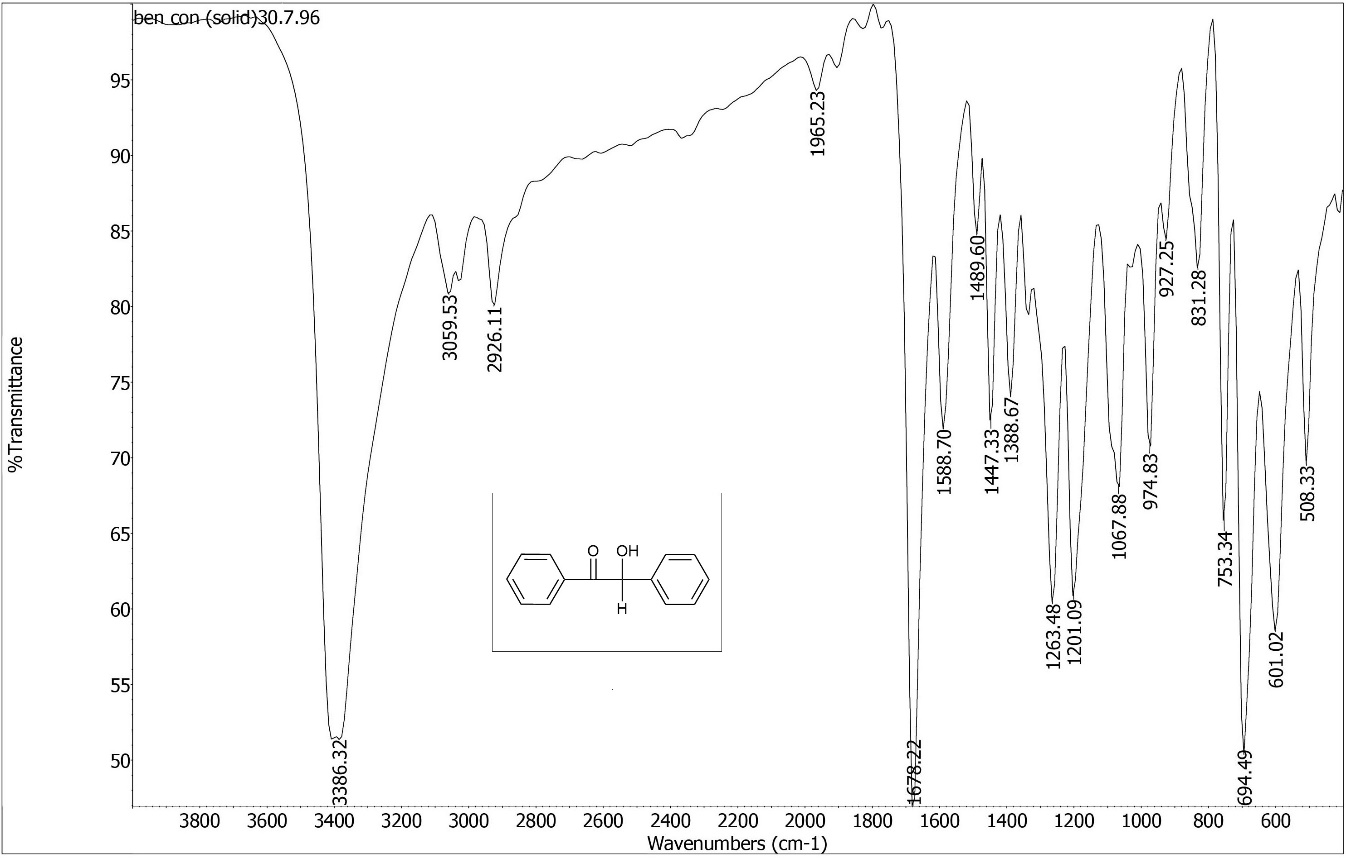


**FigureS_32_** the the FT-IR spectrum 4a


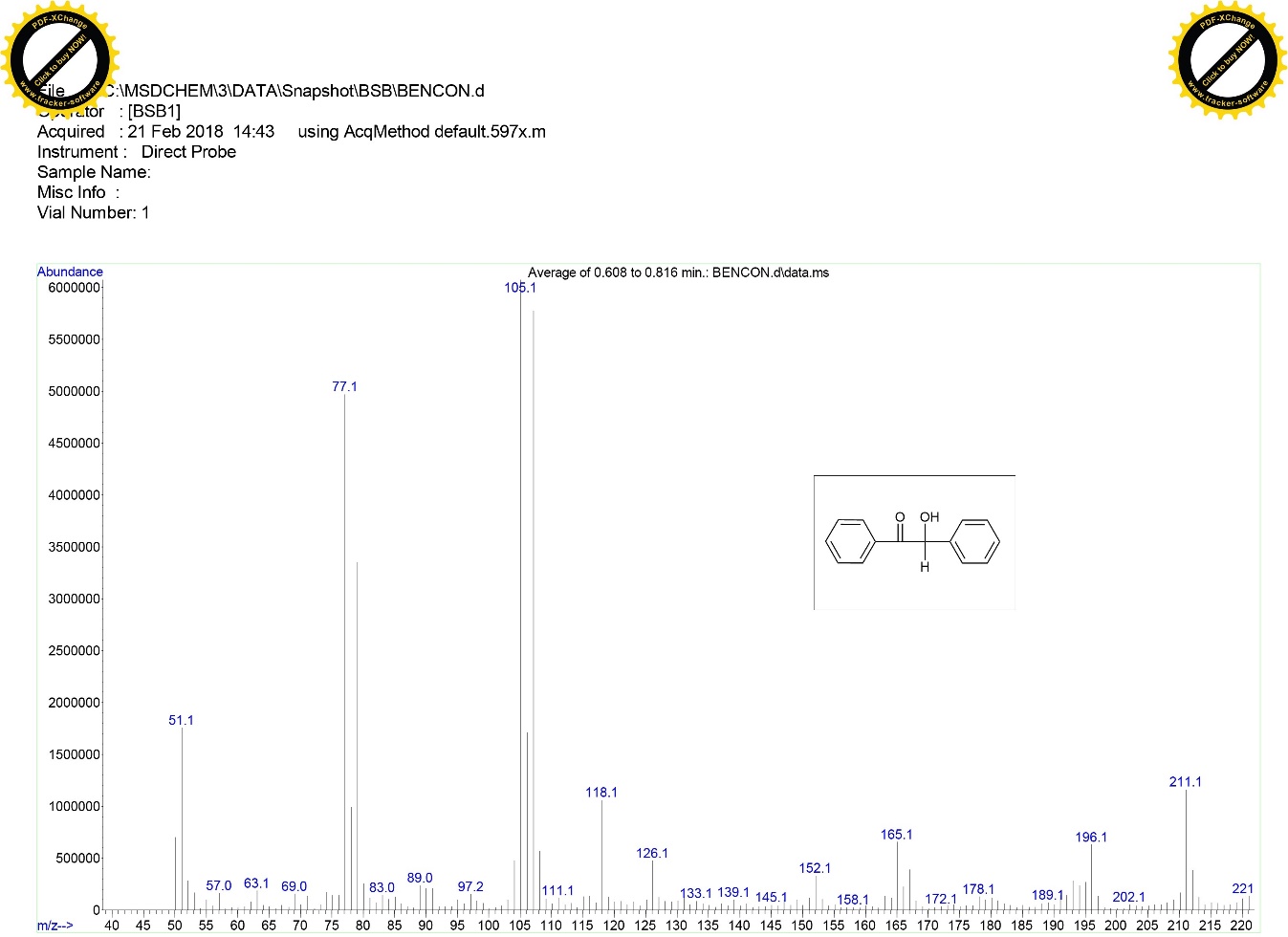


**FigureS_33_** Mass spectrum of 4a


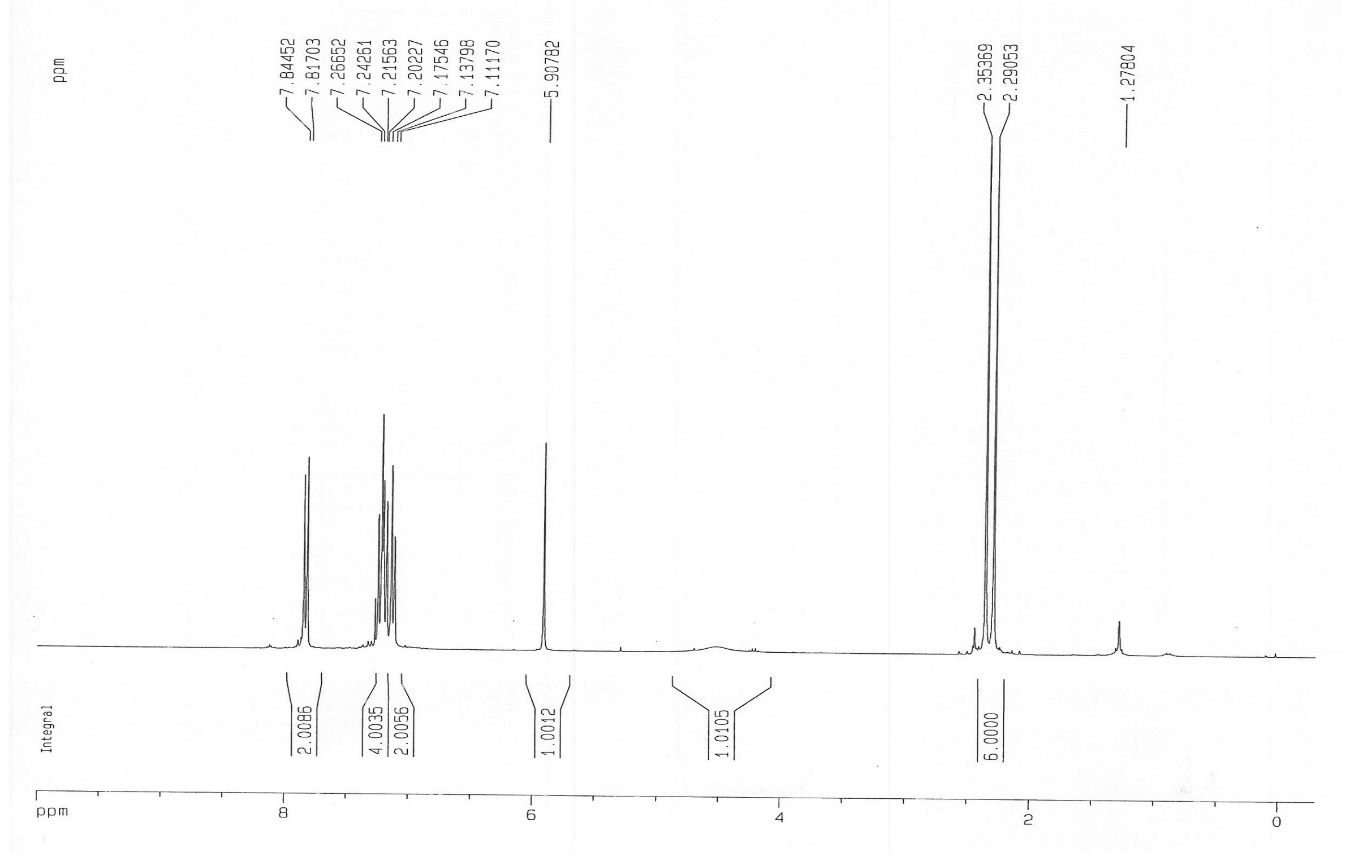


**FigureS_34_** the ^1H^ NMR spectrum of 4b in DMSO


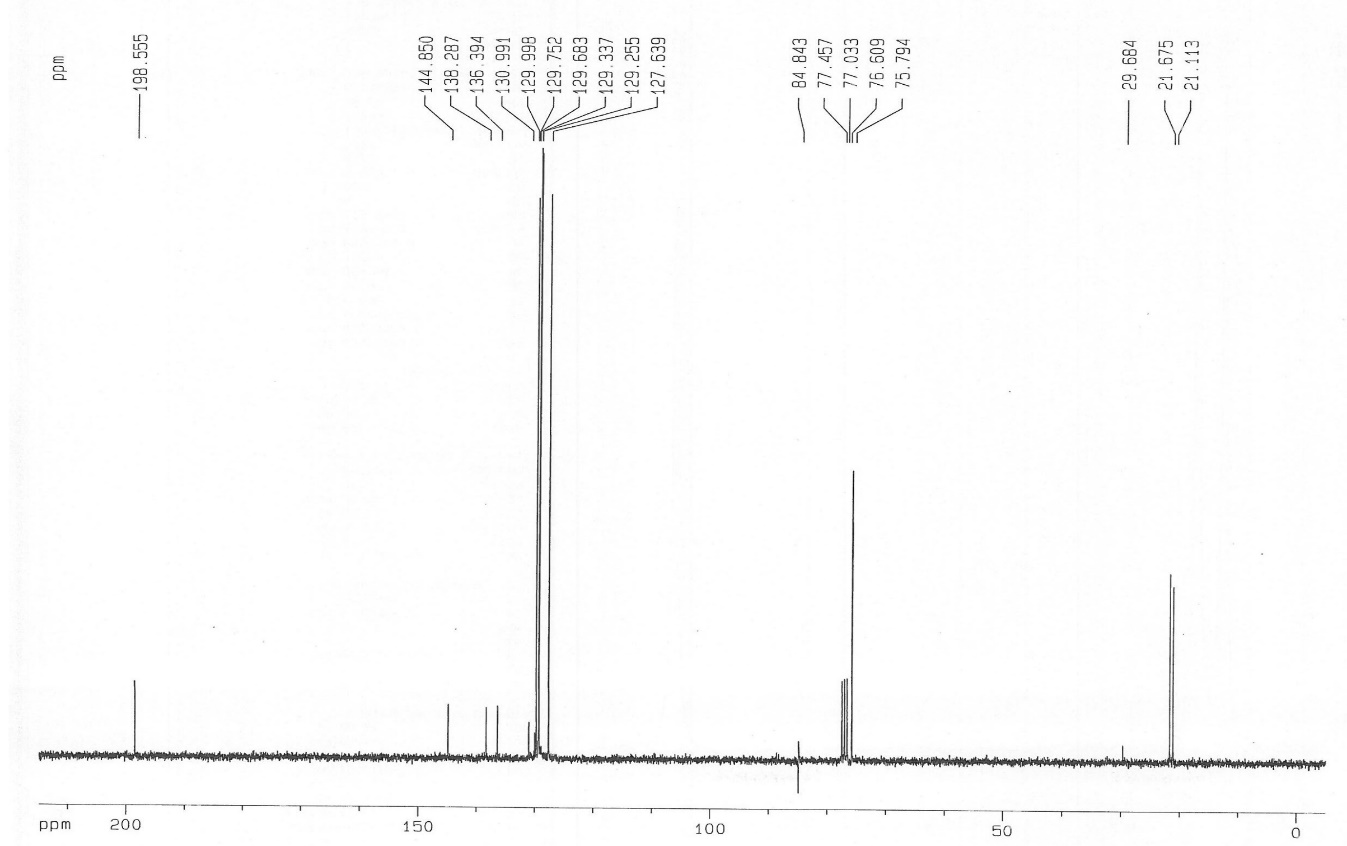


**FigureS_35_** the ^13^C NMR spectrum of 4b in DMSO


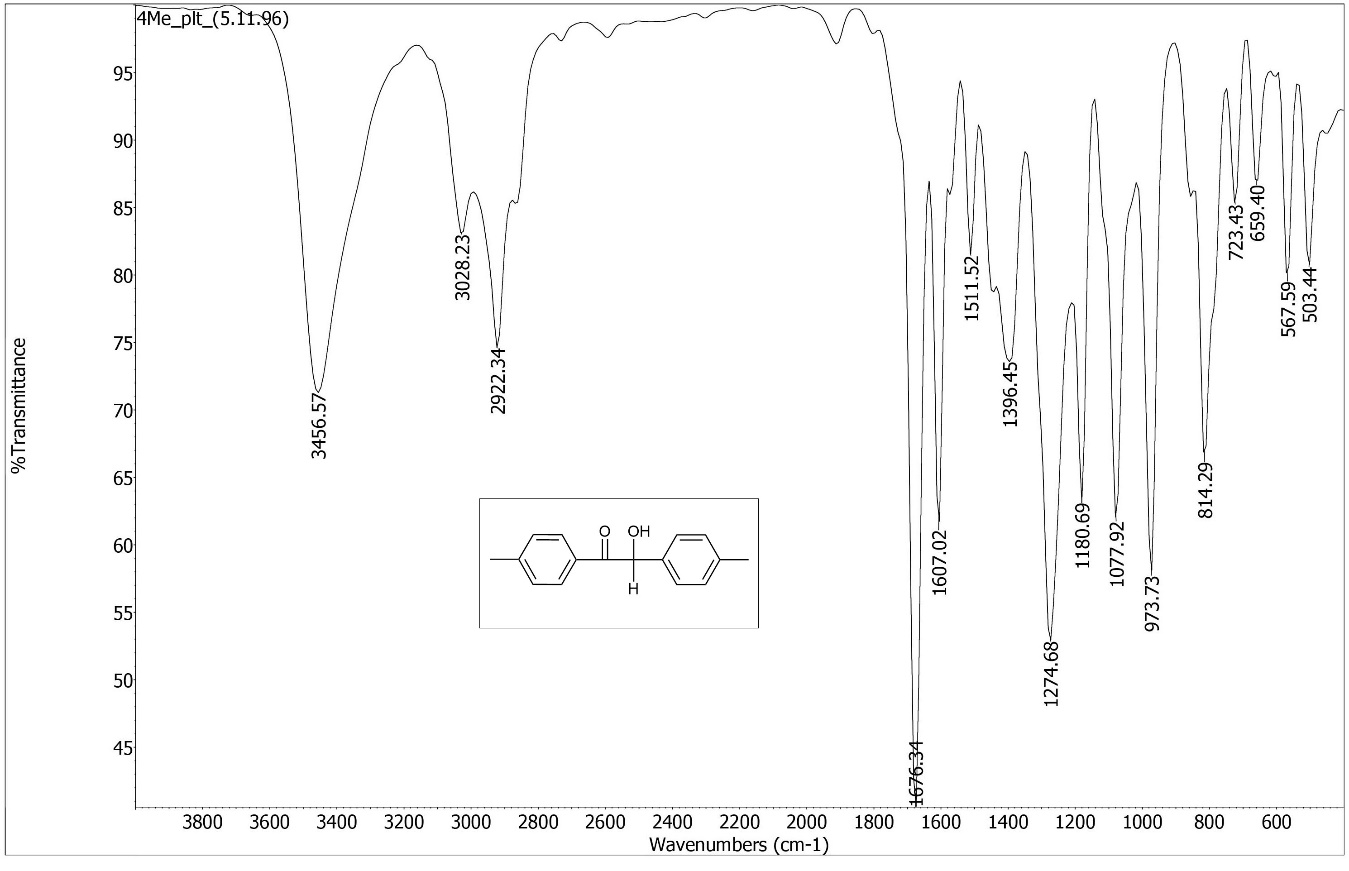


**FigureS_36_** the the FT-IR spectrum 4b


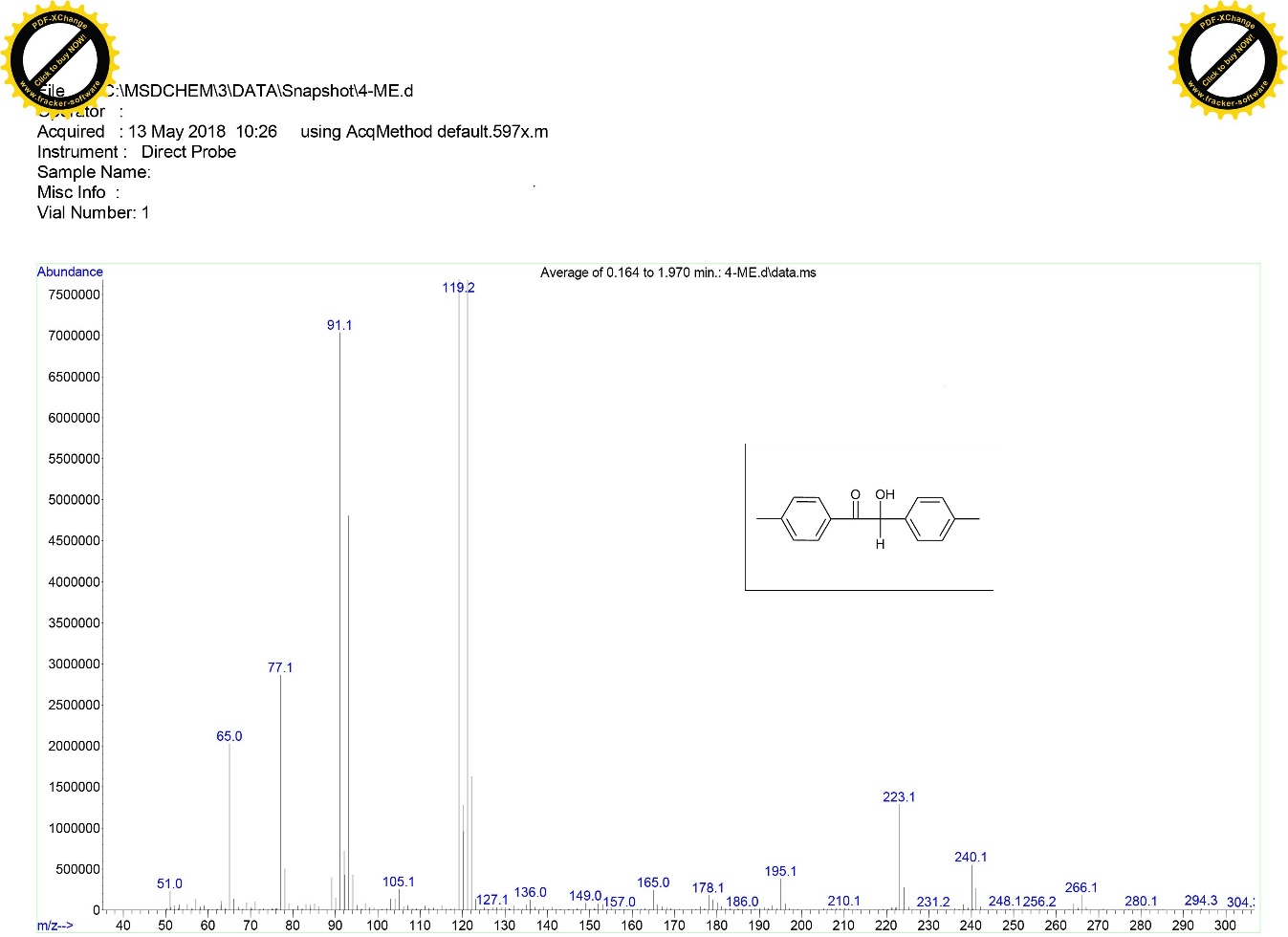


**FigureS_37_** Mass spectrum of 4b


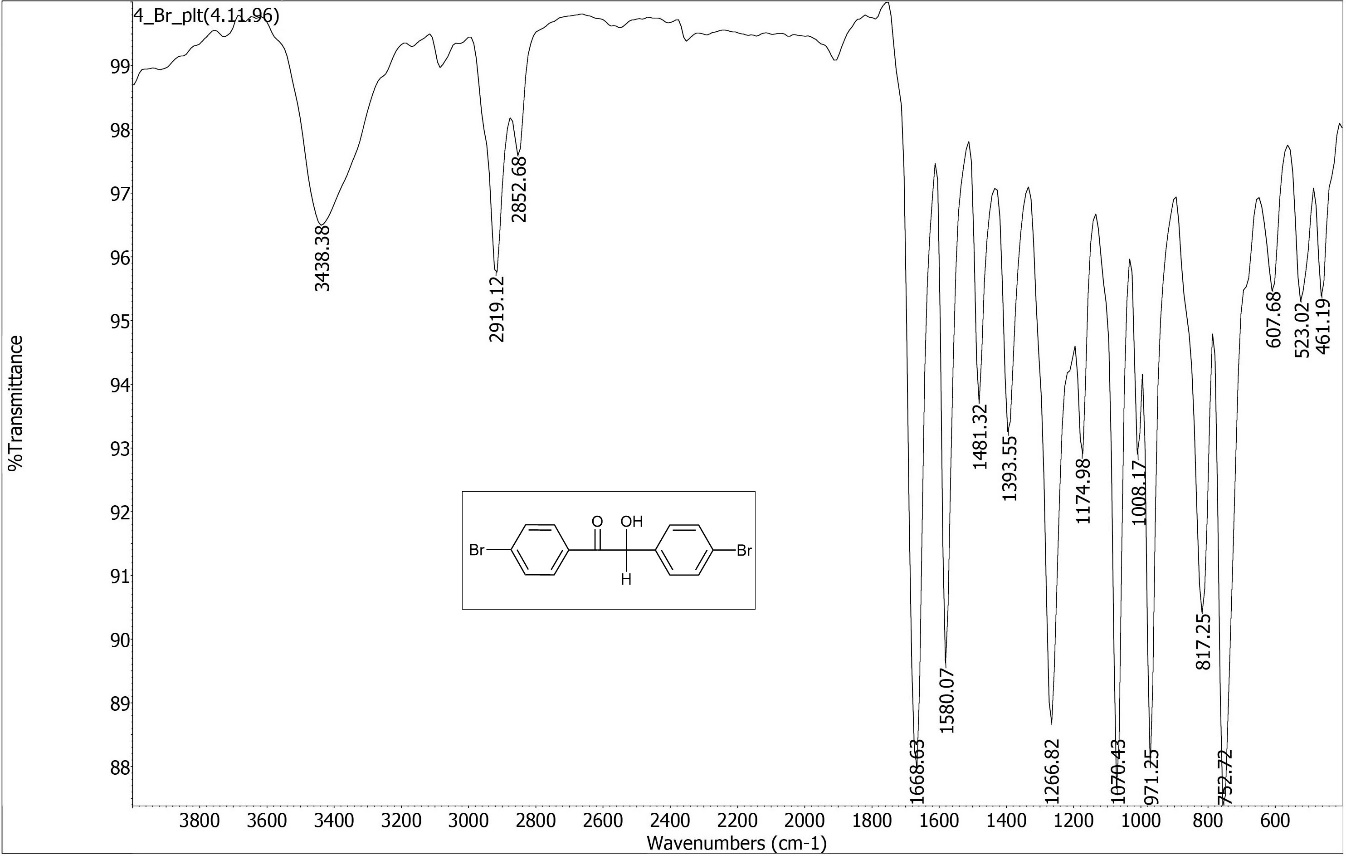


**FigureS_38_** the the FT-IR spectrum 4c


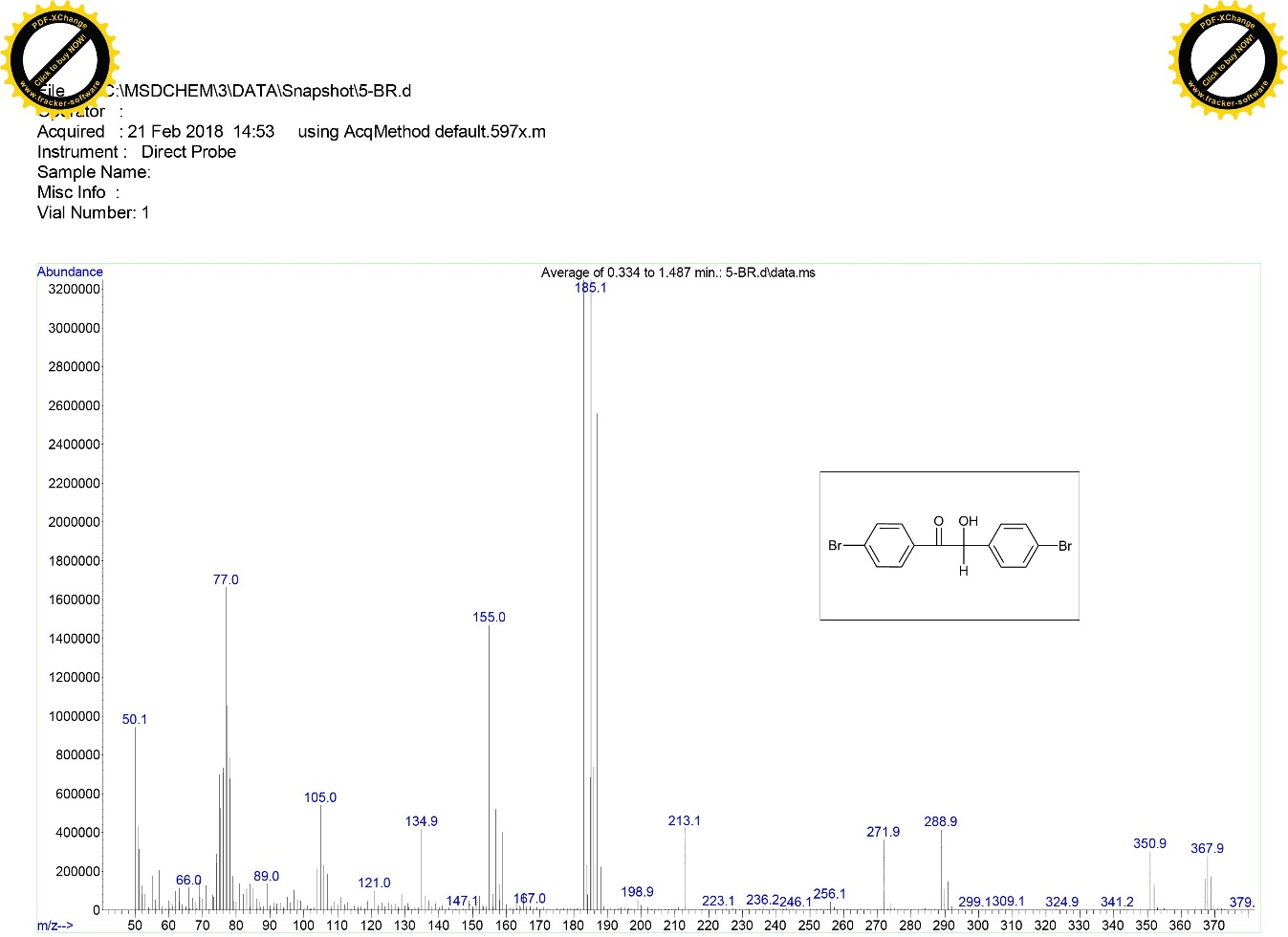


**FigureS_39_** Mass spectrum of 4c


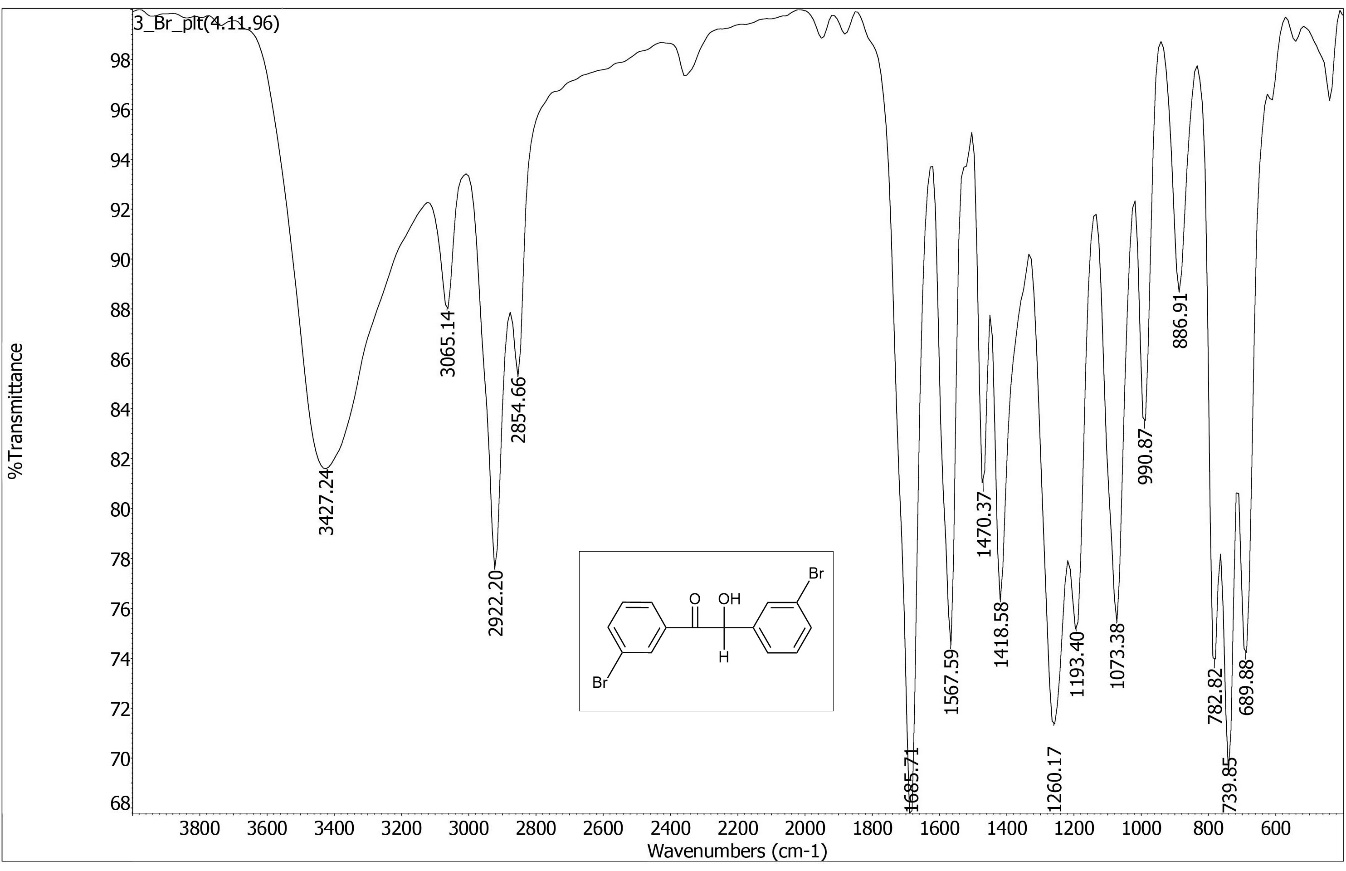


**FigureS_40_** the the FT-IR spectrum 4d


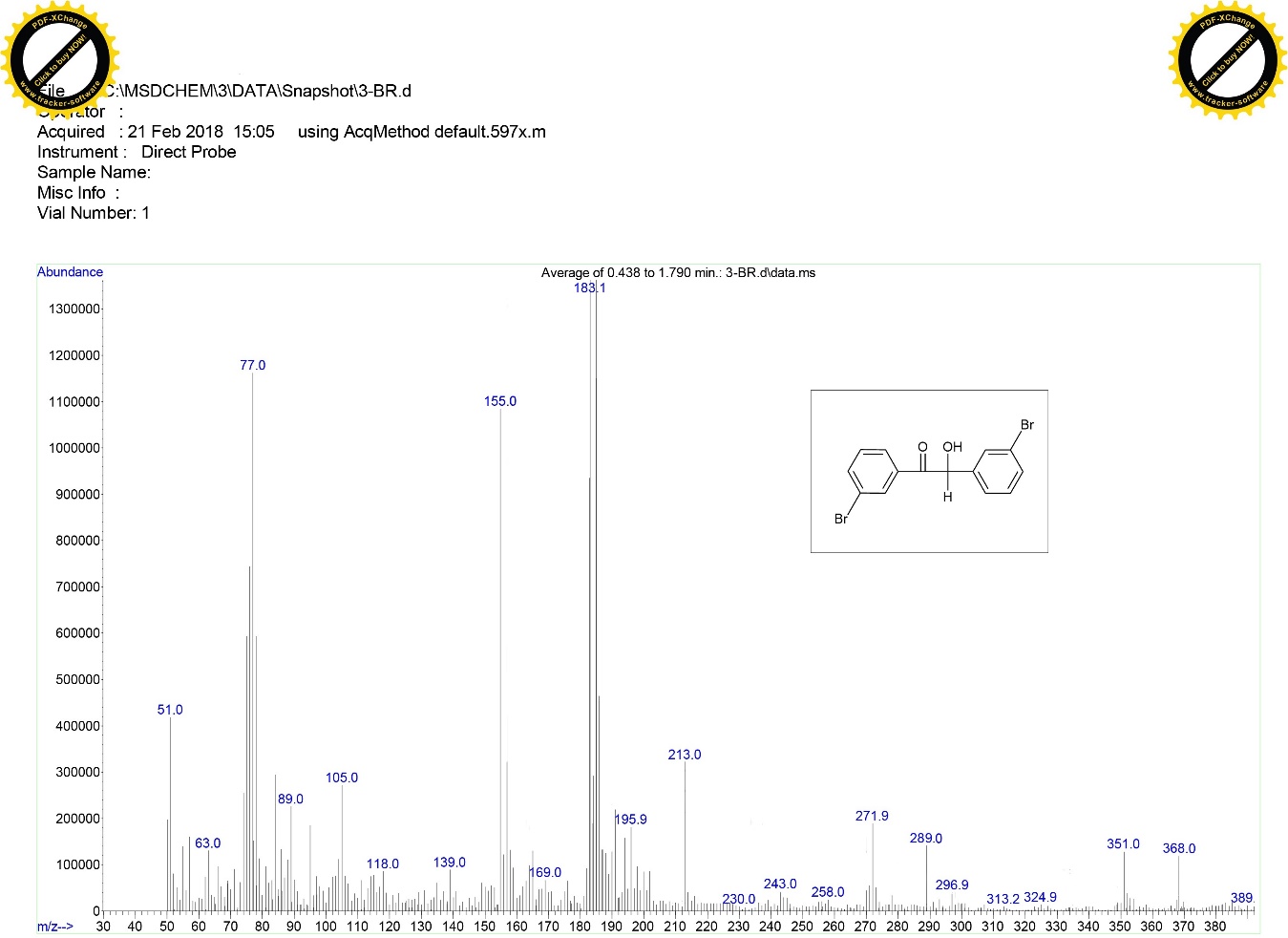


**FigureS_41_** Mass spectrum of 4d


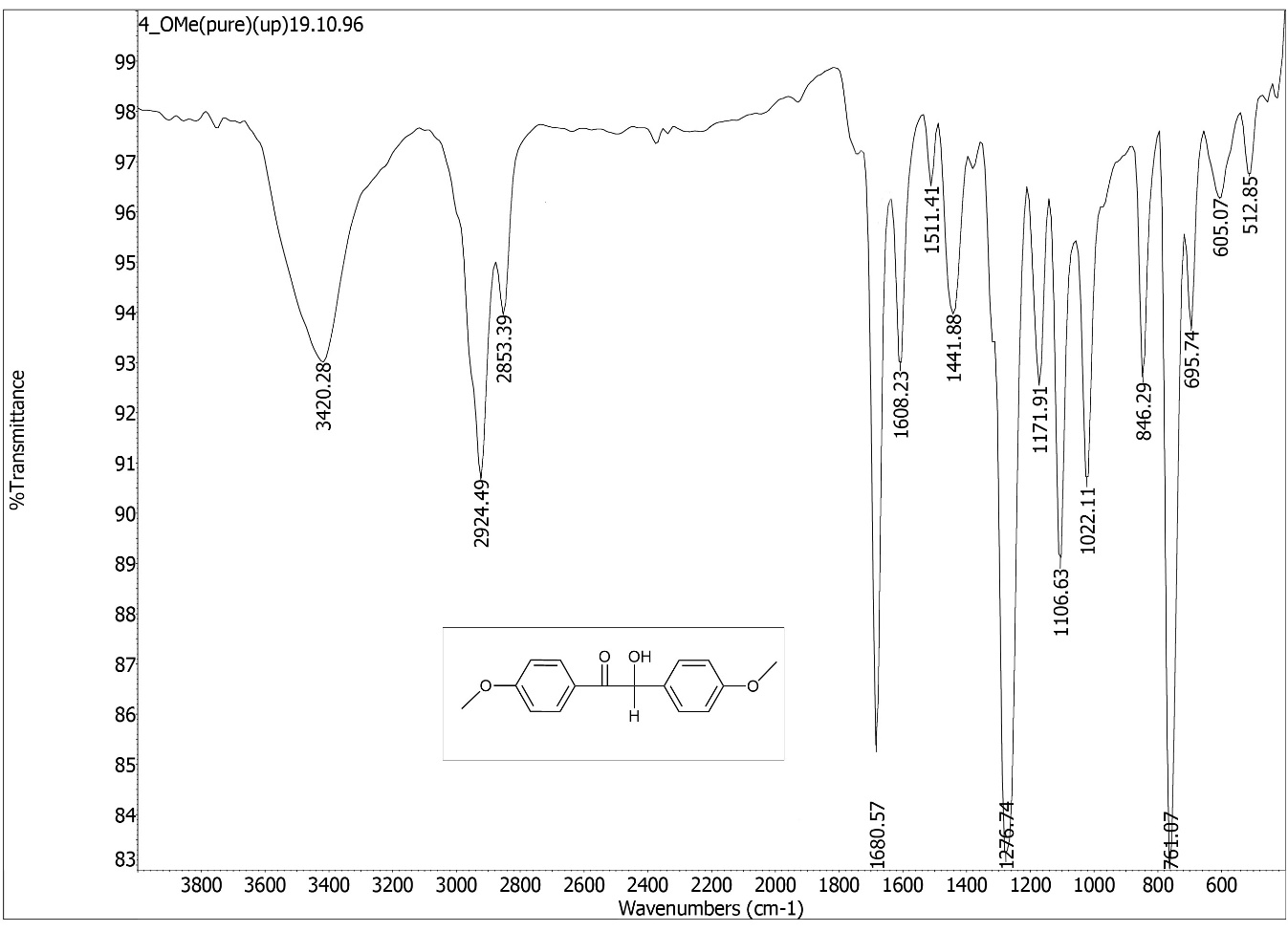


**FigureS_42_** the the FT-IR spectrum 4e


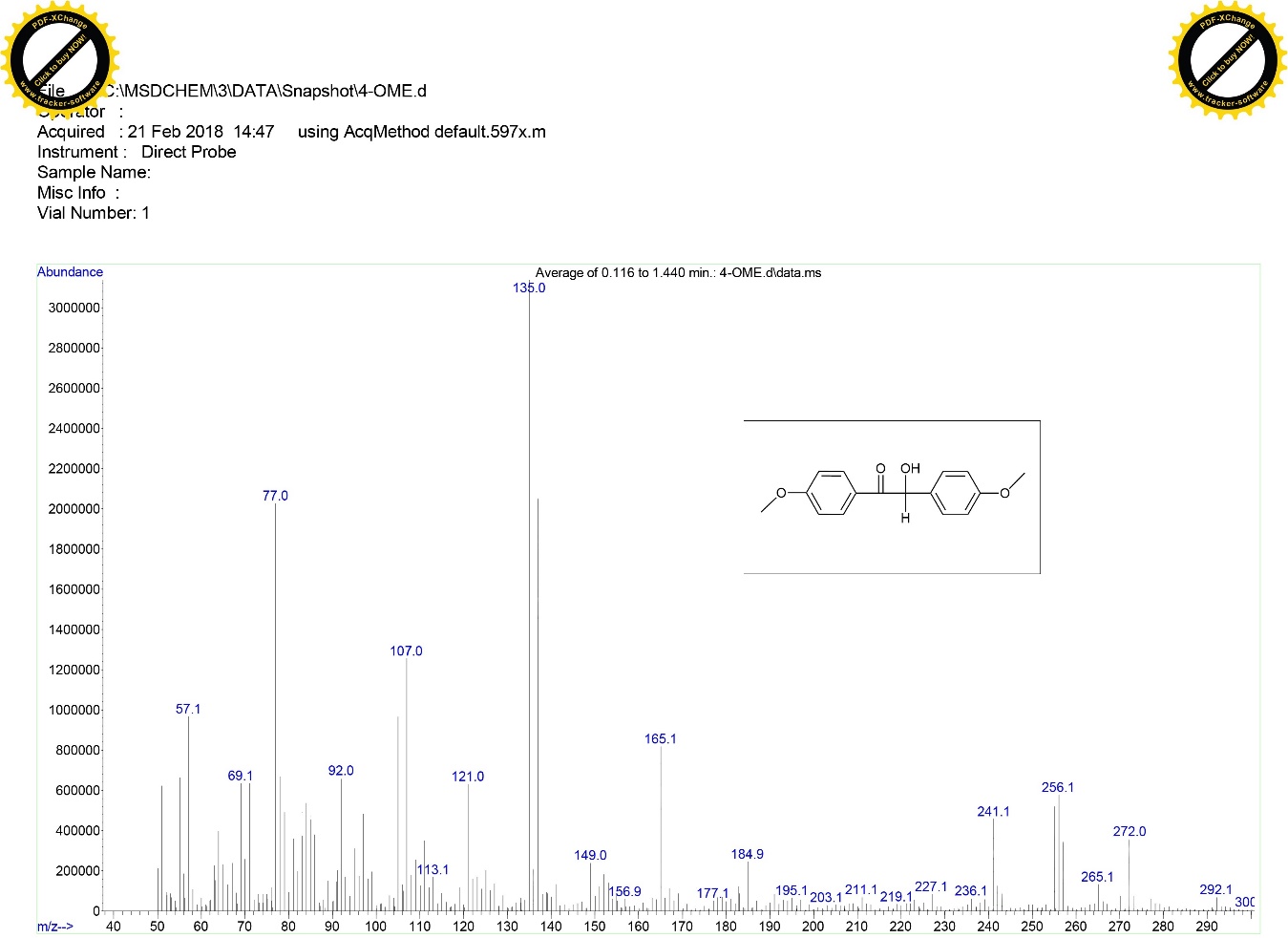


**FigureS_43_** Mass spectrum of 4e


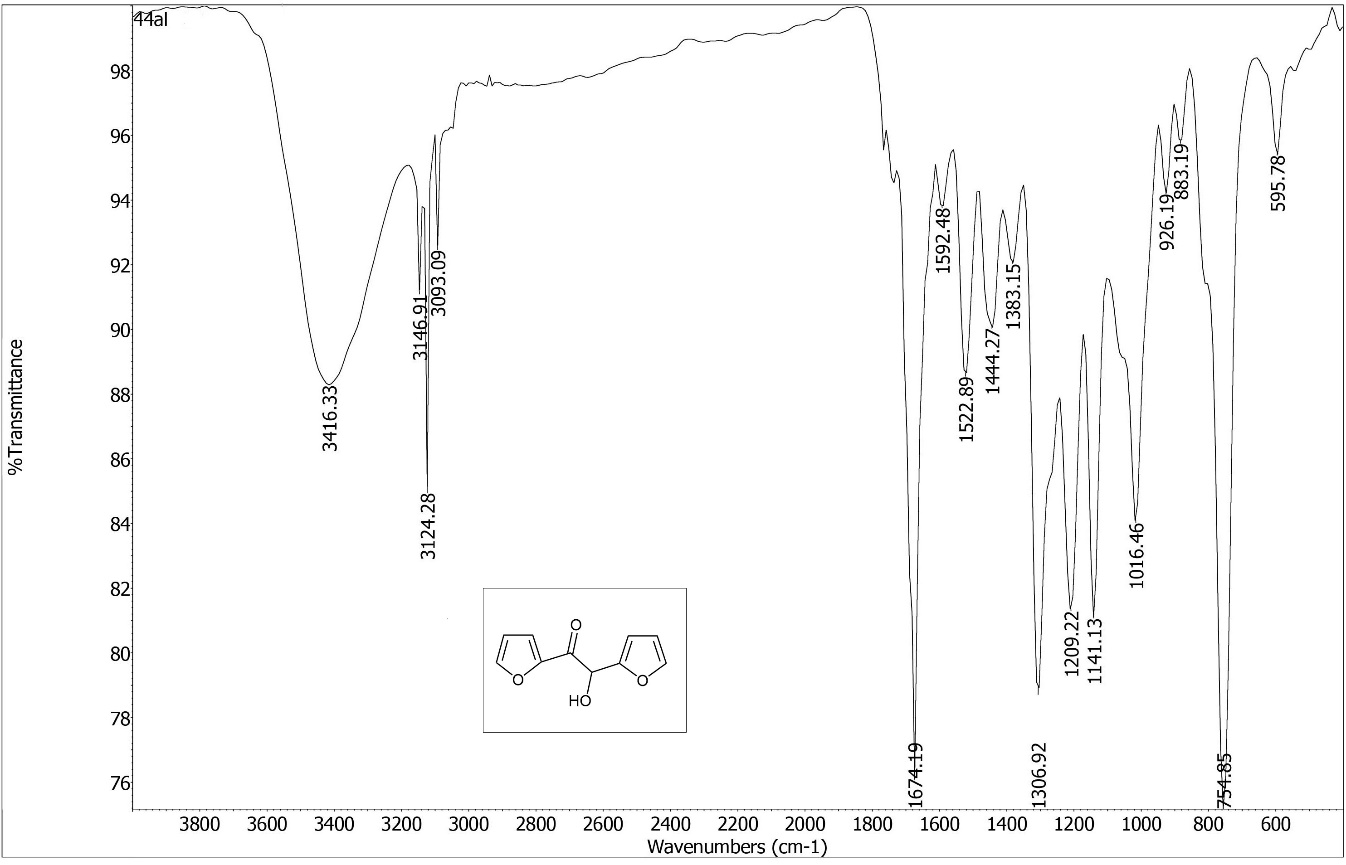


**FigureS_44_** the the FT-IR spectrum 4f


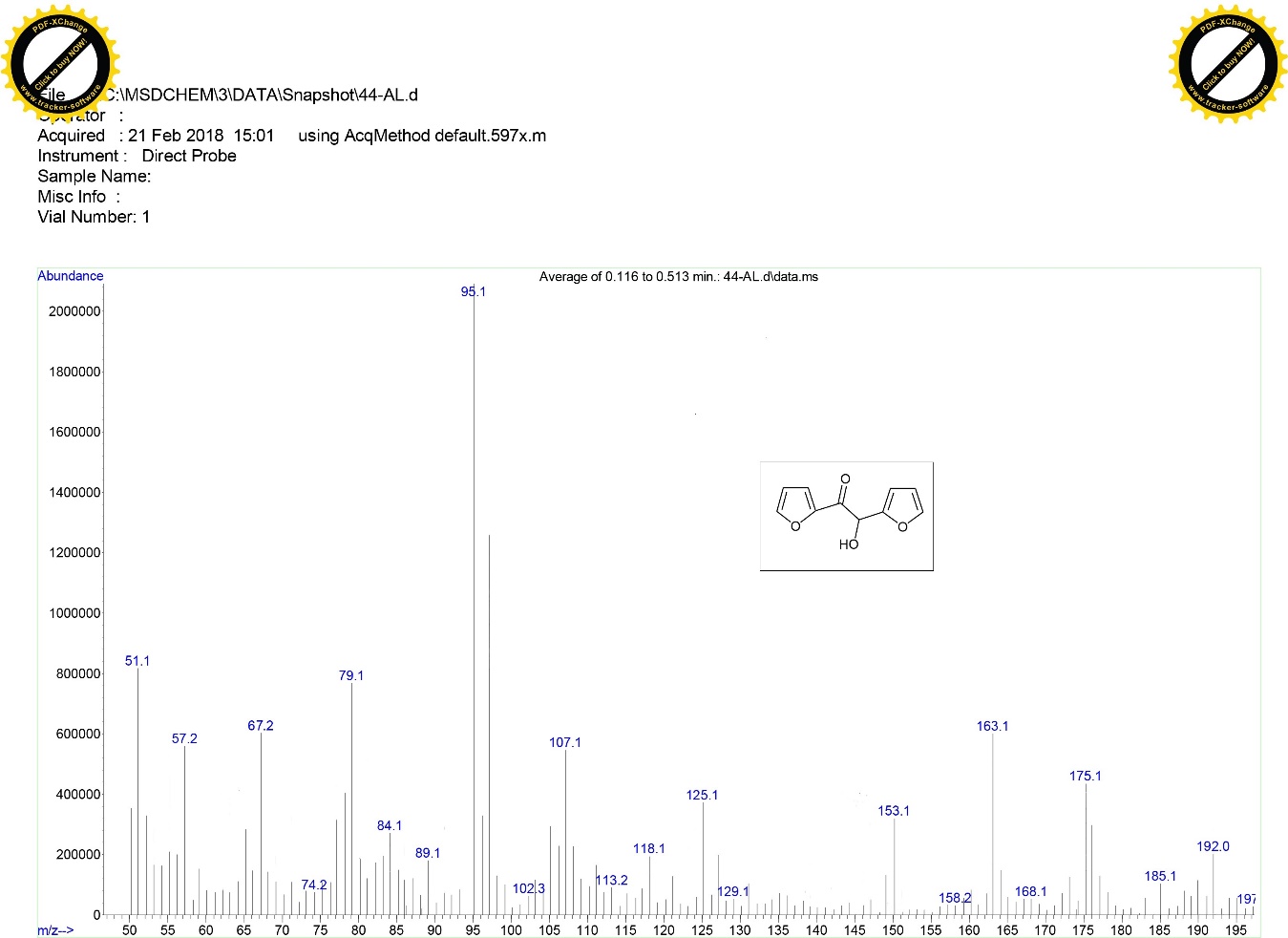


**FigureS_45_** Mass spectrum of 4f


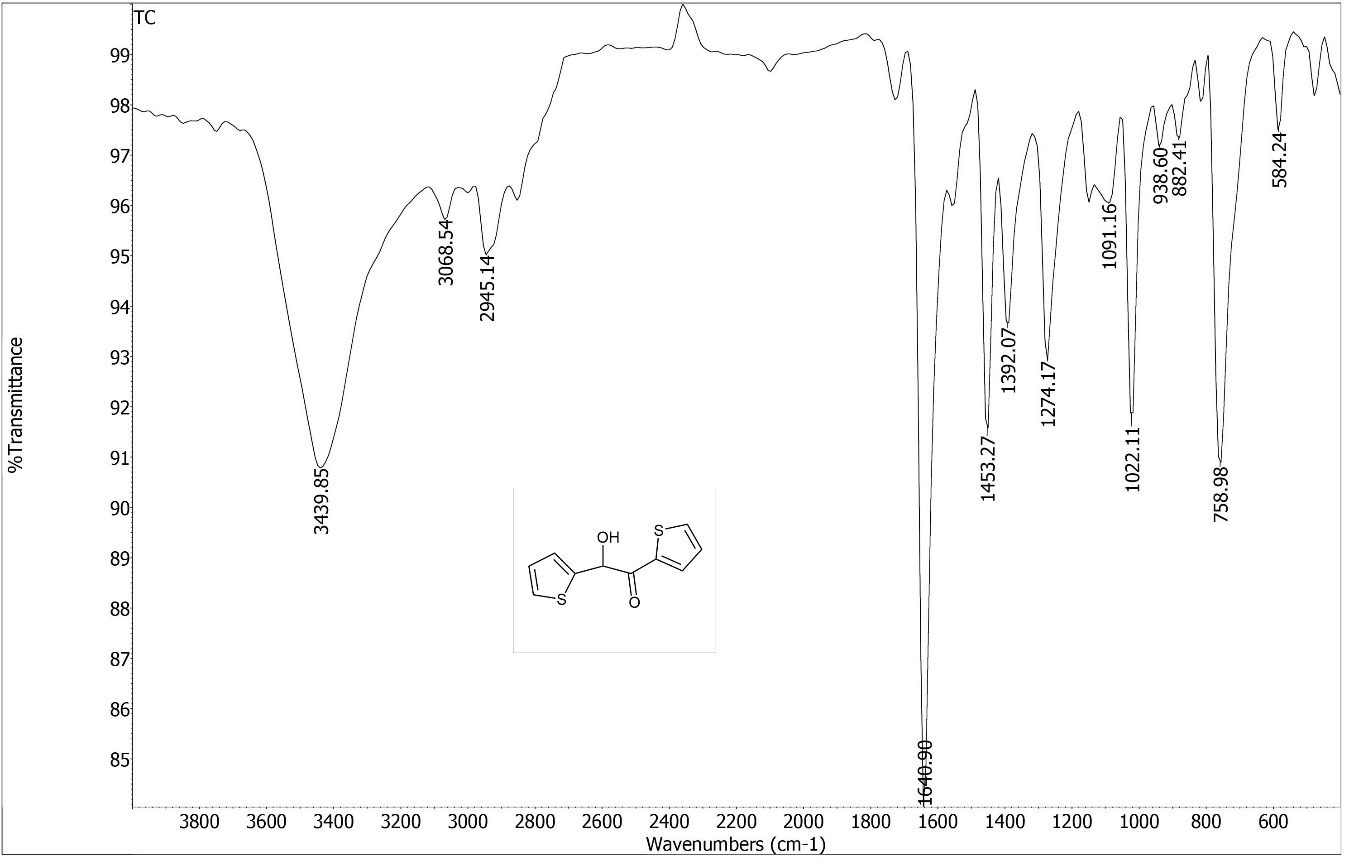


**FigureS_46_** the the FT-IR spectrum 4g


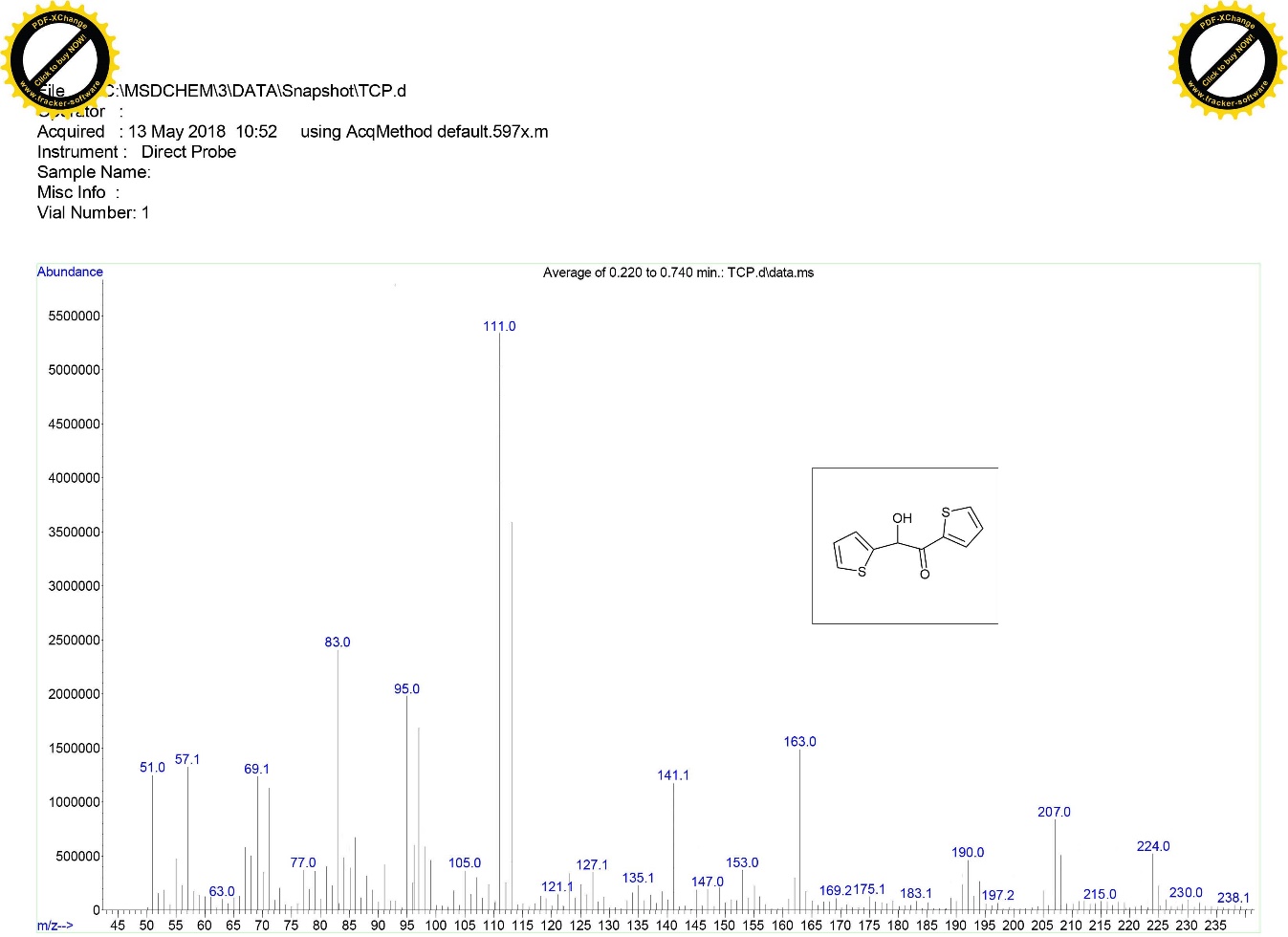


**FigureS_47_** Mass spectrum of 4g


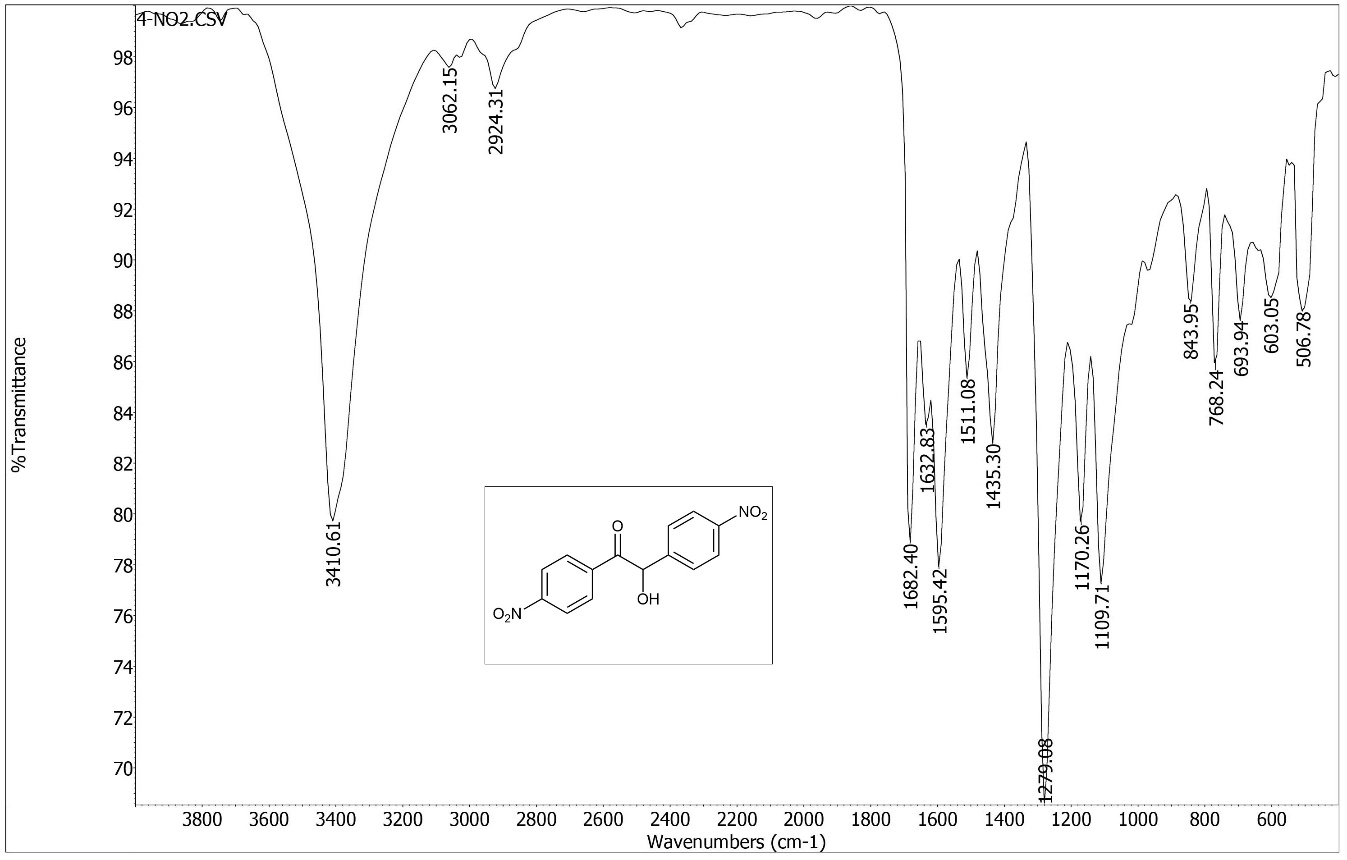


**FigureS_48_** the the FT-IR spectrum 4h

7


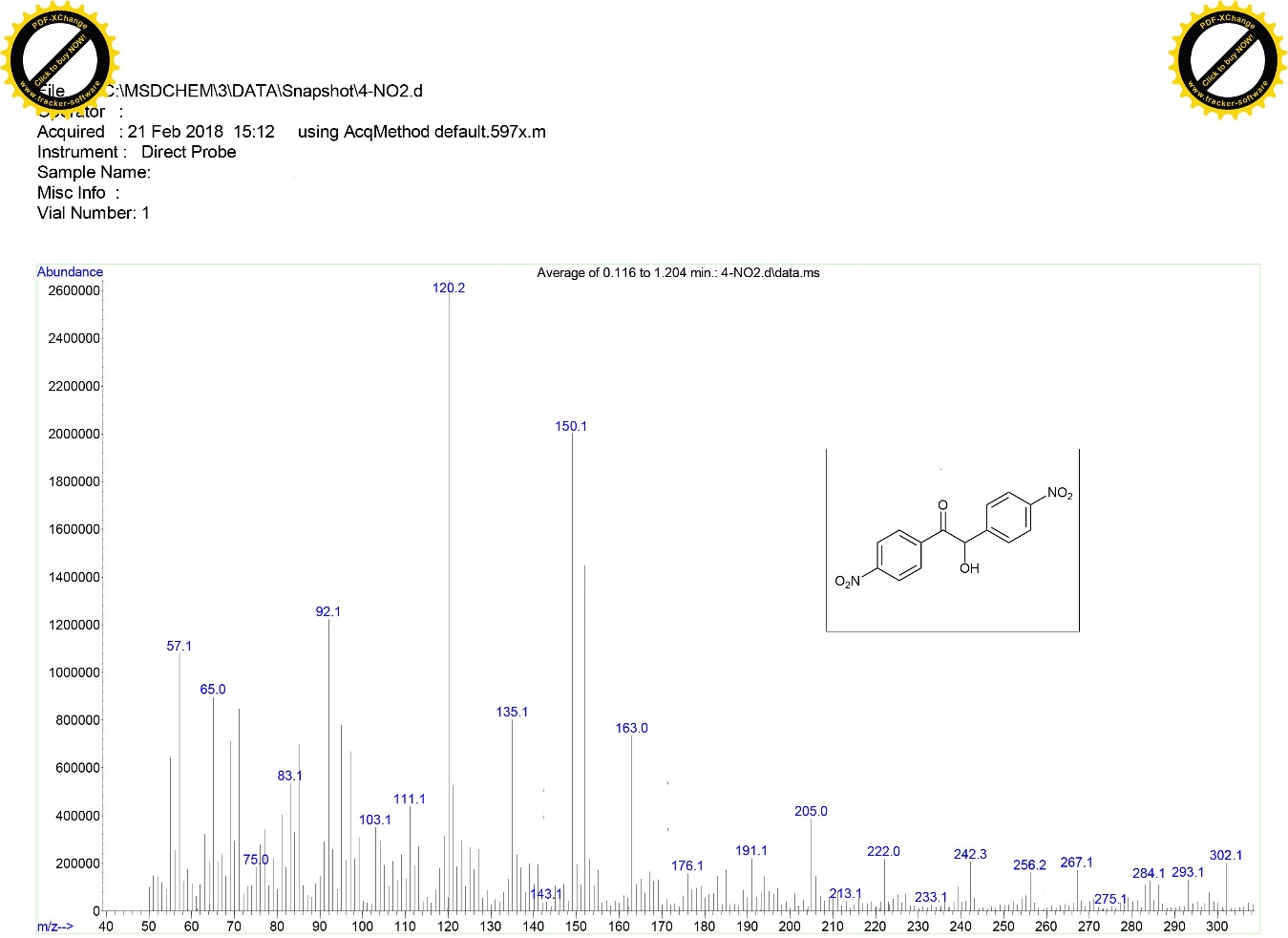


**FigureS_49_** Mass spectrum of 4h

_
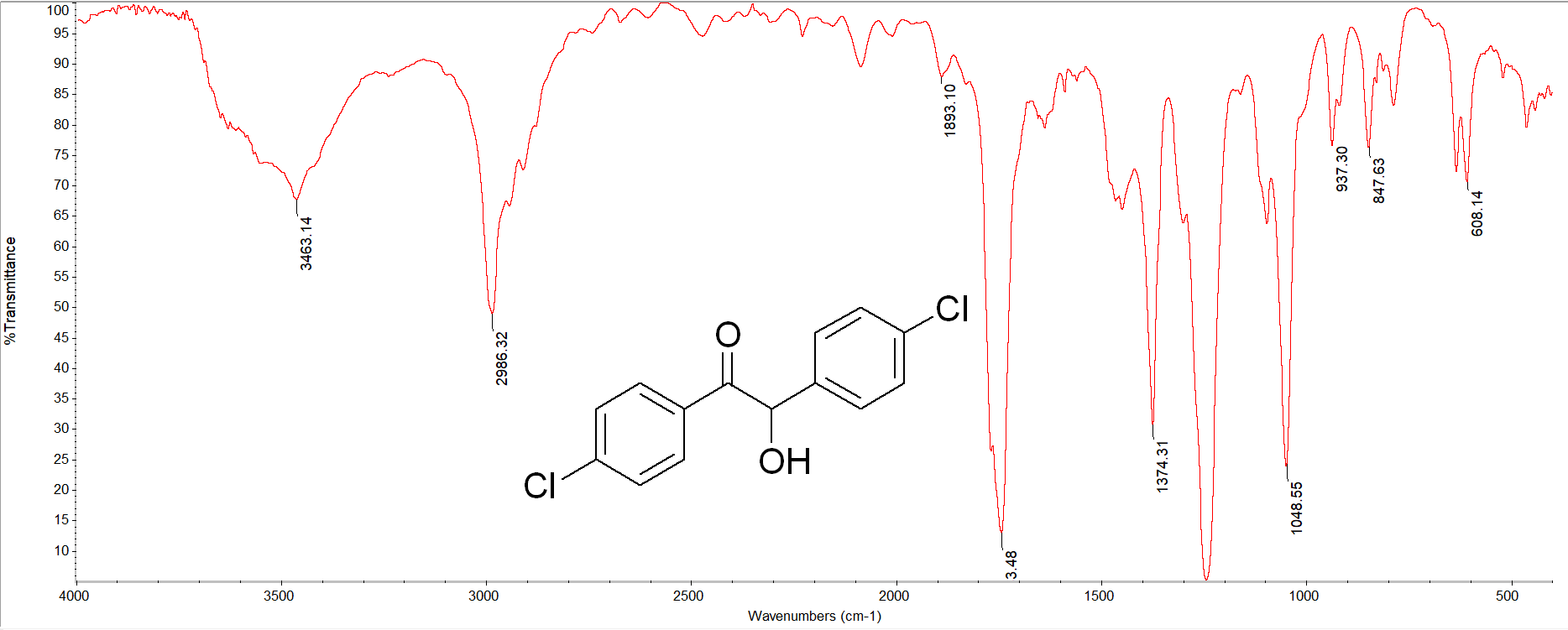
_

**FigureS_50_** the the FT-IR spectrum 4i


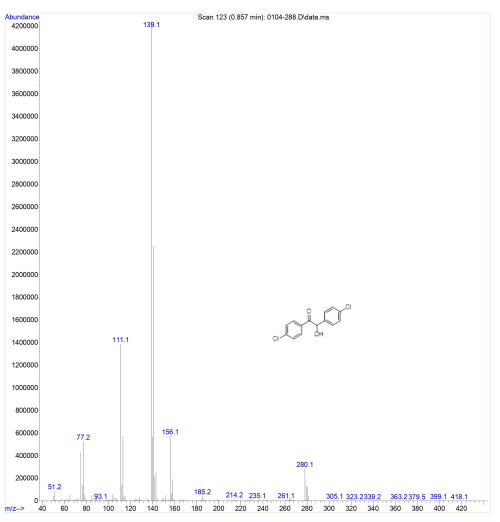


**FigureS_51_** Mass spectrum of 4i

_
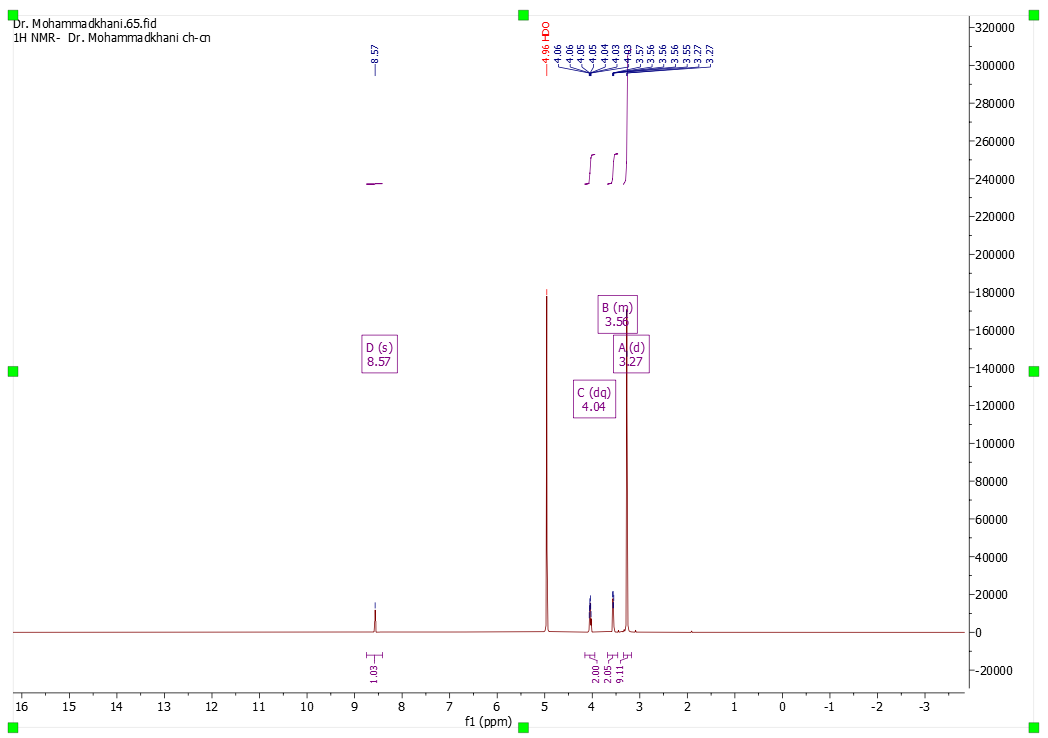
_

**FigureS_52_** the ^1^H NMR spectrum of 5a in MeOD


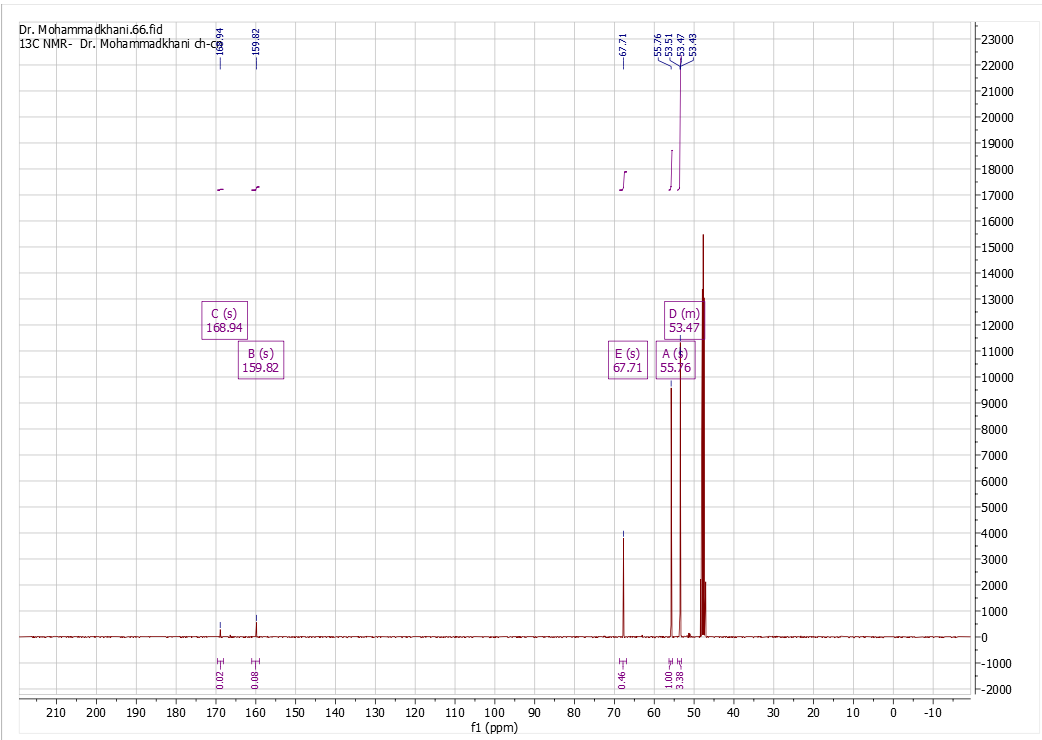


**FigureS_53_** the ^13^C NMR spectrum of 5a in MeOD


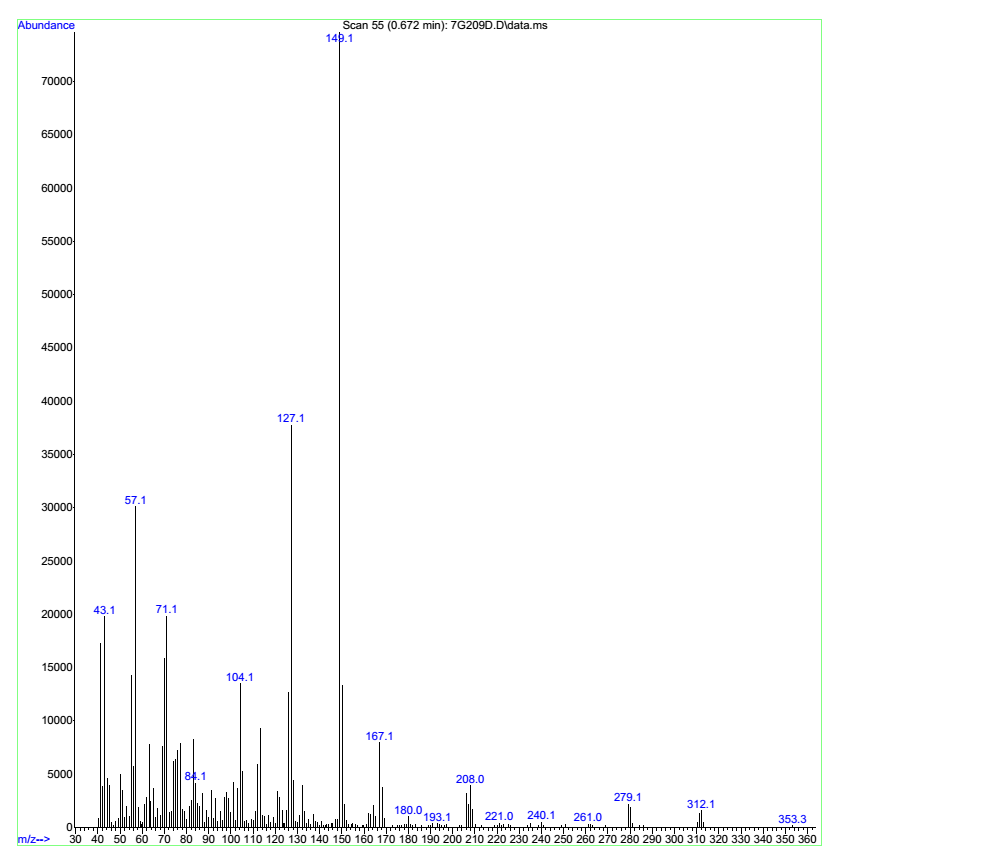


**FigureS_54_** the Mas^s^ spectrum of 5a


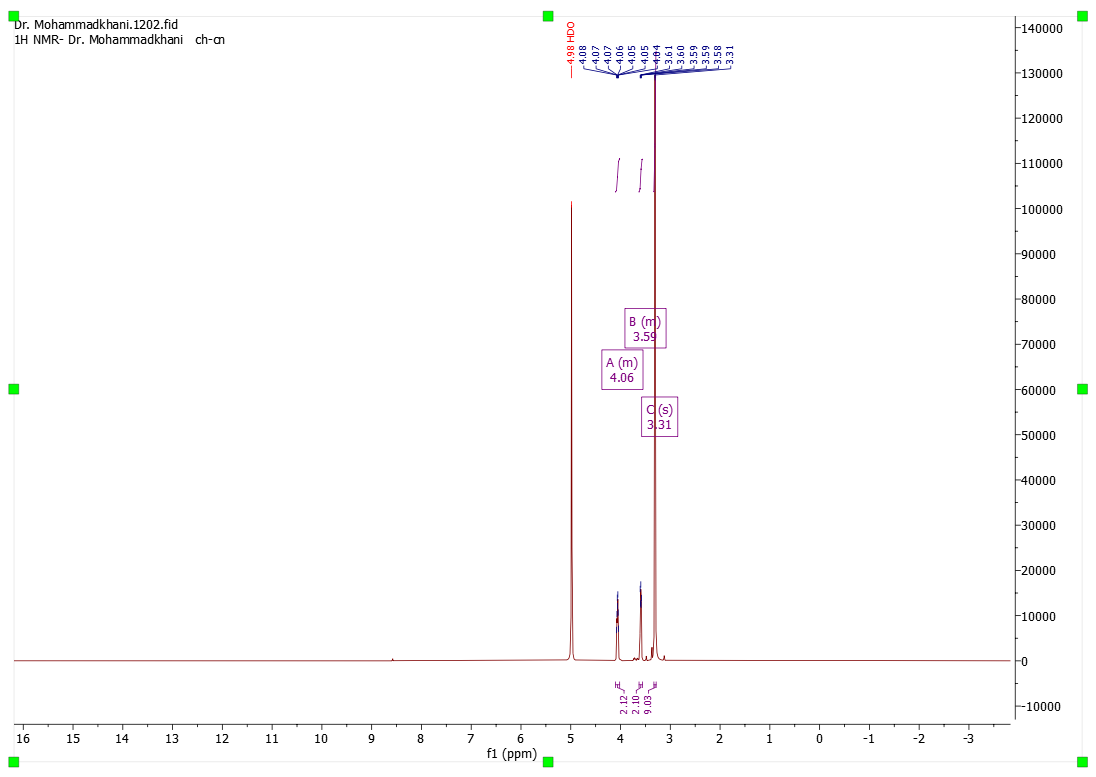


**FigureS_55_** the ^1^H NMR spectrum of 5b in MeOD


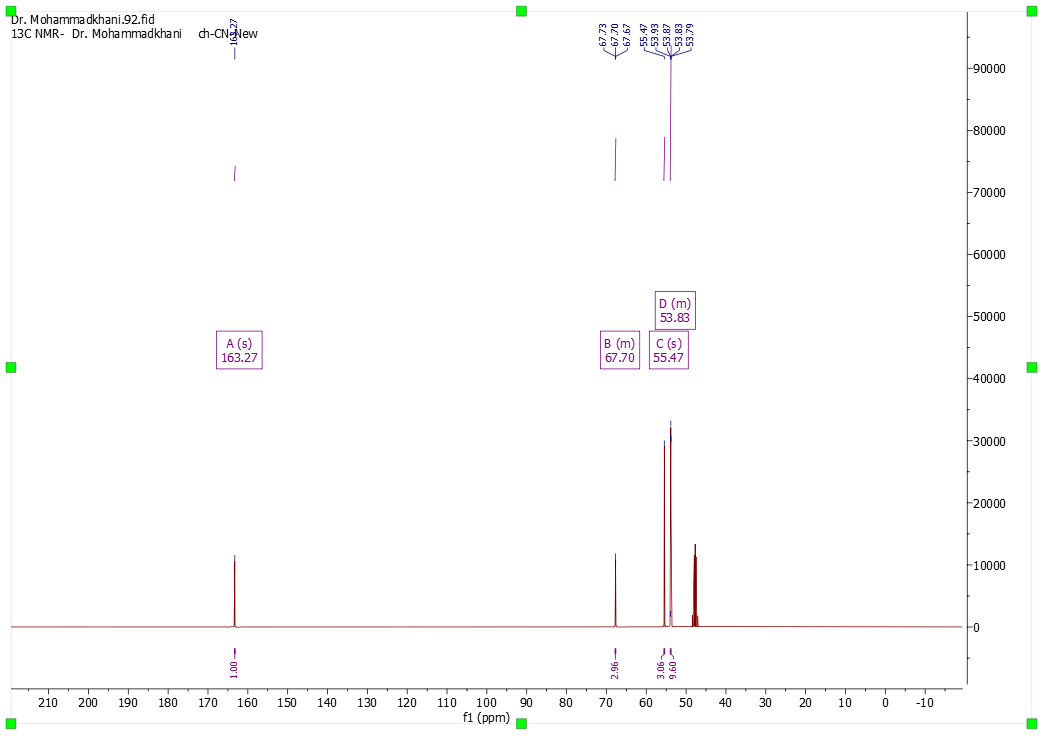


**FigureS_56_**  the ^13^C NMR spectrum of 5b in MeOD


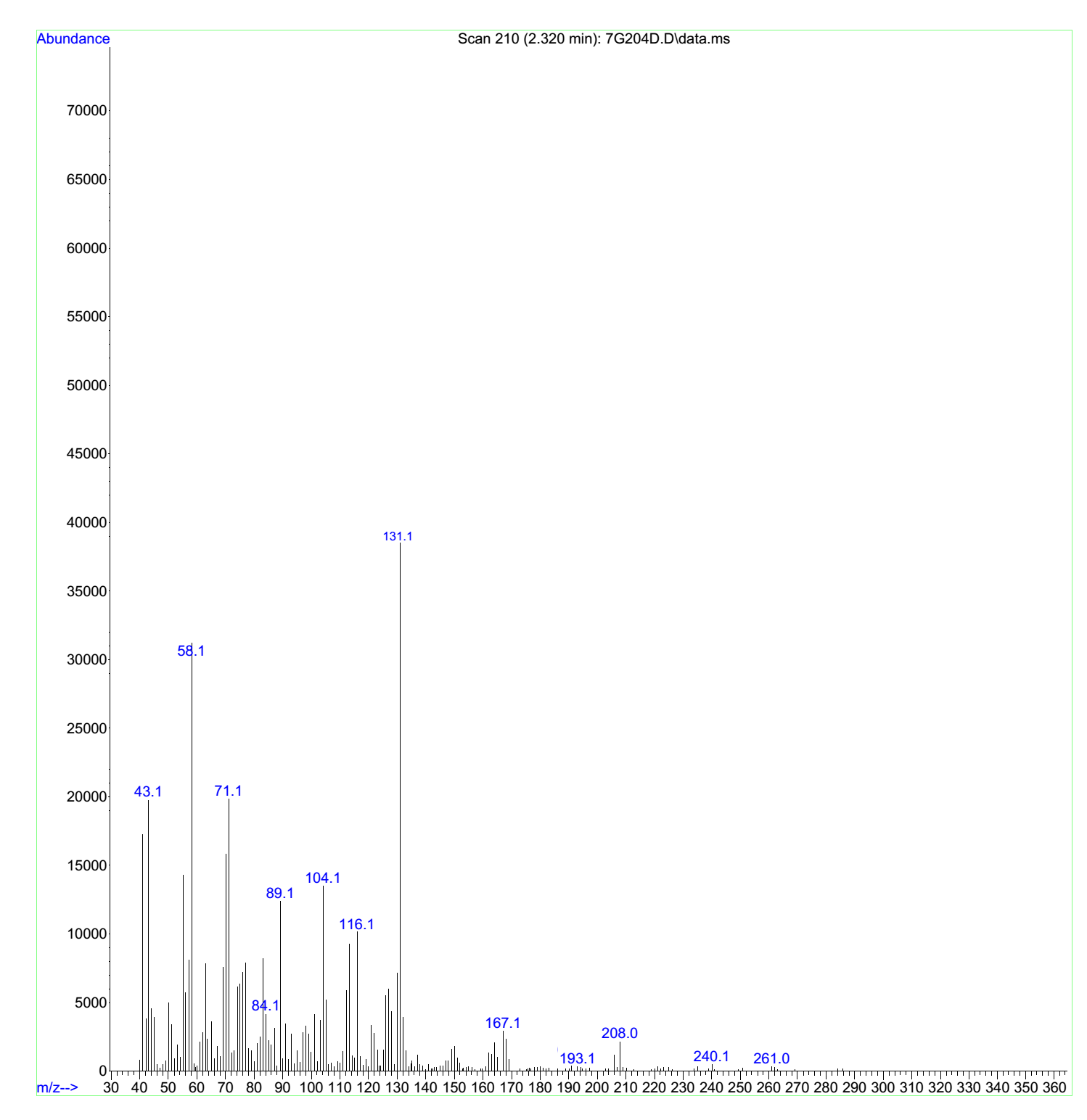


**FigureS_57_** the Mas^s^ spectrum of 5b

**Reference**

[1]. Yang H, Dong H, Zhang T, Zhang Q, Zhang G, Wang P, Liu Q (2019) Calcined dolomite: an efficient and recyclable catalyst for synthesis of α, β-unsaturated carbonyl compounds. Catal Lett 149(3):778-787.

[2]. Klikar M, Kityk I, Kulwas D, Mikysek T, Pytela O, Bureš F (2017) Multipodal arrangement of push–pull chromophores: a fundamental parameter affecting their electronic and optical properties New J Chem 41(4):1459-1472.

[3]. Zhang J, Jiang T, Han B, Zhu A, Ma X (2006) Knoevenagel condensation catalyzed by 1, 1, 3, 3‐tetramethylguanidium lactate. Synth Commun 36(22):3305-3317.

[4]. Jimenez DE, Ferreira IM, Birolli WG, Fonseca LP, Porto AL (2016) Synthesis and biocatalytic ene-reduction of Knoevenagel condensation compounds by the marine-derived fungus Penicillium citrinum CBMAI 1186. Tetrahedron 72(46):7317-7322.

[5]. Taduri AK, Devi BR (2014) Alum-Cs2CO3 as a new recyclable solid base catalyst for the efficient syntheses of arylidenemalononitriles, esters and arylcinnamic acids in water Asian J Chem 26(7):1938-1942.

[6]. Junek H, Sterk H (1968) Synthesen mit nitrilen, 19. mitt.: die partielle retro-michael-addition von tetracyanäthylen an indandion-1, 3.Tetrahedron Lett 9(40):4309-4310.

[7]. Dandia A, Jain AK, Bhati DS (2011) Direct construction of novel dispiro heterocycles through 1, 3-dipolar cycloaddition of azomethine ylides. Tetrahedron lett 52(41):5333-5337.

[8]. Ahadi S, Yasaei Z, Bazgir A (2010) A clean and one‐pot synthesis of spiroindoline‐pyranopyrazoles. J Heterocycl Chem 47(5):1090-1094.

[9]. Tan Z-Y, Wu K-X, Huang L-S, Wu R-S, Du Z-Y, Xu D-Z (2020) Iron-catalyzed cross-dehydrogenative coupling of indolin-2-ones with active methylenes for direct carbon–carbon double bond formation. Green Chem 22(2):332-335.

[10]. Shen G, Liu H, Chen J, He Z, Zhou Y, Wang L, Luo Y, Su Z, Fan B (2021) Zinc salt-catalyzed reduction of α-aryl imino esters, diketones and phenylacetylenes with water as hydrogen source. Org Biomol Chem 19(16):3601-3610.

[11]. Demir AS, Şeşenoglu Ö, Eren E, Hosrik B, Pohl M, Janzen E, Kolter D, Feldmann R, Dünkelmann P, Müller M (2002) Enantioselective synthesis of α‐hydroxy ketones via benzaldehyde lyase‐catalyzed C− C bond formation reaction. Adv Synth Catal 344(1):96-103.

[12]. Hartman W, Dickey J (1933) The preparation of furil. J Am Chem Soc 55(3):1228-1229.

[13]. Fragnelli MC, Hoyos P, Romano D, Gandolfi R, Alcántara AR, Molinari F (2012) Molinari F: Enantioselective reduction and deracemisation using the non-conventional yeast Pichia glucozyma in water/organic solvent biphasic systems: preparation of (S)-1, 2-diaryl-2-hydroxyethanones (benzoins).Tetrahedron 68(2):523-528.

[14]. Banas T, Z S (1972) CONDENSATION REACTIONS OF SOME PARA NITROBENZALDEHYDES. Roczniki Chemii 46(2):179-&.

[15]. Bläsing K, Harloff J, Schulz A, Stoffers A, Stoer P, Villinger A (2020) Salts of HCN-Cyanide Aggregates: [CN(HCN)2]− and [CN(HCN)3]−.Angew Chem Int Ed 59(26):10508-10513.
